# Supplementary material for: Toward scalable biocatalytic conversion of 5-hydroxymethylfurfural by galactose oxidase using coordinated reaction and enzyme engineering
Source: Nat Commun. 2021 Aug 16;12:4946. doi: 10.1038/s41467-021-25034-3 (PMC8367993; doi:10.1038/s41467-021-25034-3)
Supplement: Supplementary file 1 — Supplementary Information [file 41467_2021_25034_MOESM1_ESM.docx]

Supplementary Information

**Toward scalable biocatalytic conversion of 5-hydroxymethylfurfural by galactose oxidase using coordinated reaction and enzyme engineering**

William R. Birmingham^1^, Asbjørn Toftgaard Pedersen^2,4^, Mafalda Dias Gomes^2^, Mathias Bøje Madsen^2^, Michael Breuer^3^, John M. Woodley^2^, Nicholas J. Turner^1^*

^1^School of Chemistry, The University of Manchester, Manchester Institute of Biotechnology, Manchester, UK

^2^Department of Chemical and Biochemical Engineering, Technical University of Denmark, Lyngby, Denmark

^3^BASF SE, White Biotechnology Research, Ludwigshafen, Germany

^4^Present Address: Novozymes A/S, Krogshoejvej 36, Bagsvaerd, Denmark

*Corresponding author. E-mail: [nicholas.turner@manchester.ac.uk](mailto:nicholas.turner@manchester.ac.uk)

**Table of Contents Page**

[Supplementary Notes/Discussion S3](#_Toc75962445)

[Supplementary Figures S6](#_Toc75962446)

[Supplementary Tables S12](#_Toc75962447)

[Sequence Information S18](#_Toc75962448)

[Representative Chromatograms S20](#_Toc75962449)

[Supplementary References S55](#_Toc75962450)

# Supplementary Notes/Discussion

**First Round of GOase Evolution**

The GOase M_3-5_ variant previously evolved for *R*-selective oxidation of secondary benzylic alcohols^1^ was selected as a starting point for engineering via previously described active site CASTing libraries A-G^2^ (Figure 2). We anticipated that screening against a generic substrate of known low activity could lead to an overall more active enzyme, and therefore chose 1‑hexanol as a model substrate for use in our previously reported colorimetric solid phase screen^1,2^. Several apparent hits with improved activity over the M_3-5_ progenitor were identified, with variant M_4_ (M_3-5_ + Y329L and M330F mutations) demonstrating the highest activity toward HMF. Kinetic characterization of M_3-5_ and M_4_ revealed very little difference between the two enzymes, both having high k_cat,app_ values as well as very low K_M,app_ values (Table 2), however M_4_ gave much better performance in initial trials (see Reaction Engineering section of main text, and Note below) and therefore became the template for subsequent engineering.

**Screen at Low O_2_ Atmosphere**

The O_2_ monitors in the glovebox read ~0.2%, however due to the nature of the assay setup it would likely have been slightly higher during the experiment. Water, equilibrated overnight in the glovebox, was removed to make the agarose solution in the microwave at normal atmosphere, and while this was performed as quickly as possible, the agar solution would have begun to re-equilibrate to normal oxygen saturation levels. Once returned and aliquoted in the glovebox (see Methods for protocol details), the agarose solution would have then begun a rapid re-equilibration to the low oxygen content. The low level of oxygen in the glovebox is expected to be critical for this last re-equilibration to quickly reduce soluble oxygen content to a level that provides a selection pressure in the assay. Early attempts at ~3% oxygen atmosphere in the glovebox during assay development lead to too much activity in the screen (not significantly different than on the benchtop), indicating that re-equilibration in the glovebox after making the agarose solution was much too slow.

**DFF Over-Oxidation**

With our recent characterization of aldehyde oxidase activity in GOase M_3-5_ ^3^, over-oxidation of the desired DFF product to 5-formyl-2-furan carboxylic acid (FFCA) was an obvious concern. Therefore, initially biocatalytic reactions were performed in varying buffer strength with the expectation that a lower buffering capacity might reduce the formation of the acidic over-oxidation product because of reaction quenching via pH change. Interestingly, in all cases we found the level of over-oxidation to be relatively modest, while buffer strength indeed had a large impact on the rate of HMF oxidation and final conversion (Supplementary Table 2) as we also previously reported^4^. For each pair of reactions, the M_4_ variant proved to be more effective for HMF oxidation, with the greatest activity observed at 100 mM sodium phosphate (NaPi) reaching 93% conversion of 100 mM HMF to DFF after 8 h with only 4% over-oxidation to FFCA.

**Initial Solvent Screen**

Reactions containing either 10% DMSO or no co-solvent at all (Supplementary Table 3) led to dramatic increases in activity compared to those with 5% acetonitrile (Supplementary Table 2), highlighting the influence of co-solvent selection. Complete conversion of 100 mM HMF was achieved within 1 h and 2 h using 10% DMSO or no co-solvent, respectively, with both reactions reaching approximately 20% over-oxidation to FFCA after 8 h (Supplementary Table 3). Because the increased formation of FFCA coincided with an increase in rate and level of conversion, we reasoned that this would serve as a convenient internal indicator that reaction intensification could be pushed further, i.e. decrease biocatalyst loading and/or increase substrate loading, to maximize productivity. Indeed, reducing the biocatalyst loading by half still allowed high conversions to DFF at both 100 mM (94% in 3 h) and 250 mM (71% after 8 h) substrate loading. However, both reactions with 500 mM HMF stalled after ~30% conversion to DFF (Supplementary Table 4), showing that the biocatalyst is unsurprisingly sensitive to deactivation (or inhibition) at high substrate loadings.

**Identification solvents with lower vapour pressures**

Alternative solvents to the initially well performing ethyl acetate were required to improve performance at larger scale where aeration is needed to supply the required oxygen. At a typical aeration rate of 1 volume gas/ volume reactor / minute (vvm) it is estimated that up to 23 g solvent/L reactor/h is lost when using ethyl acetate as co-solvent. This means that almost all the co-solvent will be lost with the off-gas during the course of a typical reaction (24 hours). Cooling the off-gas to 5°C will only limit the loss to 10 g/L/h due to the large volumes of air and therefore low concentration of solvent in the off-gas.

Biocatalytic reactions will at large scale be conducted in large stirred reactors with good mixing to facilitate mass transfer of oxygen from the air to the aqueous phase. The residence time of the gas phase will therefore be sufficiently long to assume that the off-gas will be saturated with solvent. Furthermore, neat solvent will always be present in the reactor since the water miscibility of the organic solvent will be low meaning that the loss of solvent can be estimated by knowing the saturated vapour pressure (p_SAT_) and the volume of air going through the reactor. The vapour pressure is estimated using the Antoine equation:

$${log(p}_{SAT})=A-\frac{B}{C+T}$$

Where *A*, *B* and *C* is empirically determined coefficient specific to the substance and *T* is the temperature in Kelvin.

The parameters of the Antoine equation can for most common solvents be found in the scientific literature.

The evaporation rate is then given from the ideal gas law

$$F=\frac{\frac{p_{SAT}}{P}\cdot V_{T}\cdot P}{R\cdot T}$$

Where *F* is the rate of solvent loss (mol solvent/L reactor/min), *P* is the pressure in the reactor (typically 1 atm), *V_T_* is the volumetric air flow rate (L air/ L reactor/ min), *R* is the gas constant and *T* is the temperature.

Supplementary Figure 2 show calculated evaporation rates for water and three of the experimentally investigated solvents. Diethyl carbonate and butyl acetate show significantly lower evaporation rates than ethyl acetate.

**Unfavorable Co-Solvents**

Cyclohexanone and ethyl acetoacetate were examined as potential replacements, although the former led to only 15% conversion of 250 mM HMF and the latter resulted in no conversion to DFF (as well as the formation of other unknown species in the HPLC chromatogram). Viewing these results together with the reduced conversion in the presence of acetone (Supplementary Table 7, Entry 5), it appears that co-solvents containing a ketone moiety are not well tolerated by GOase. This reinforces the suggestion that the aldehyde products of alcohol oxidation (or dialdehyde in the case of DFF) are particularly harmful to GOase activity, potentially through modification of solvent accessible lysine residues.

**Semi-Crude HMF**

BASF provided a new preparation of semi-crude HMF that had undergone a partial purification step via treatment with activated charcoal prior to solubilisation in DEC. Since this semi-crude HMF in DEC is a more relevant and representative form of the substrate to be used for industrial implementation, it was used for all subsequent biocatalytic reactions to characterize GOase performance.

**Initial Assessment of 0.2 L Reaction Conditions**

At a 0.2 L scale, initial reaction conditions used to provide high oxygen transfer to the aqueous phase (1 vvm air (volume/volume/minute), 1000 rpm stirring and/or high catalase loading) resulted in excessive foaming, even in the presence of antifoam. Subsequent reactions employed lower aeration and stirring rates with reduced catalase loading, and no antifoam (in-house evidence suggested that polyethers reduce GOase activity, possibly through copper ion chelation), which resulted in conditions suitable for preliminary comparison of the three GOase variants.

# Supplementary Figures


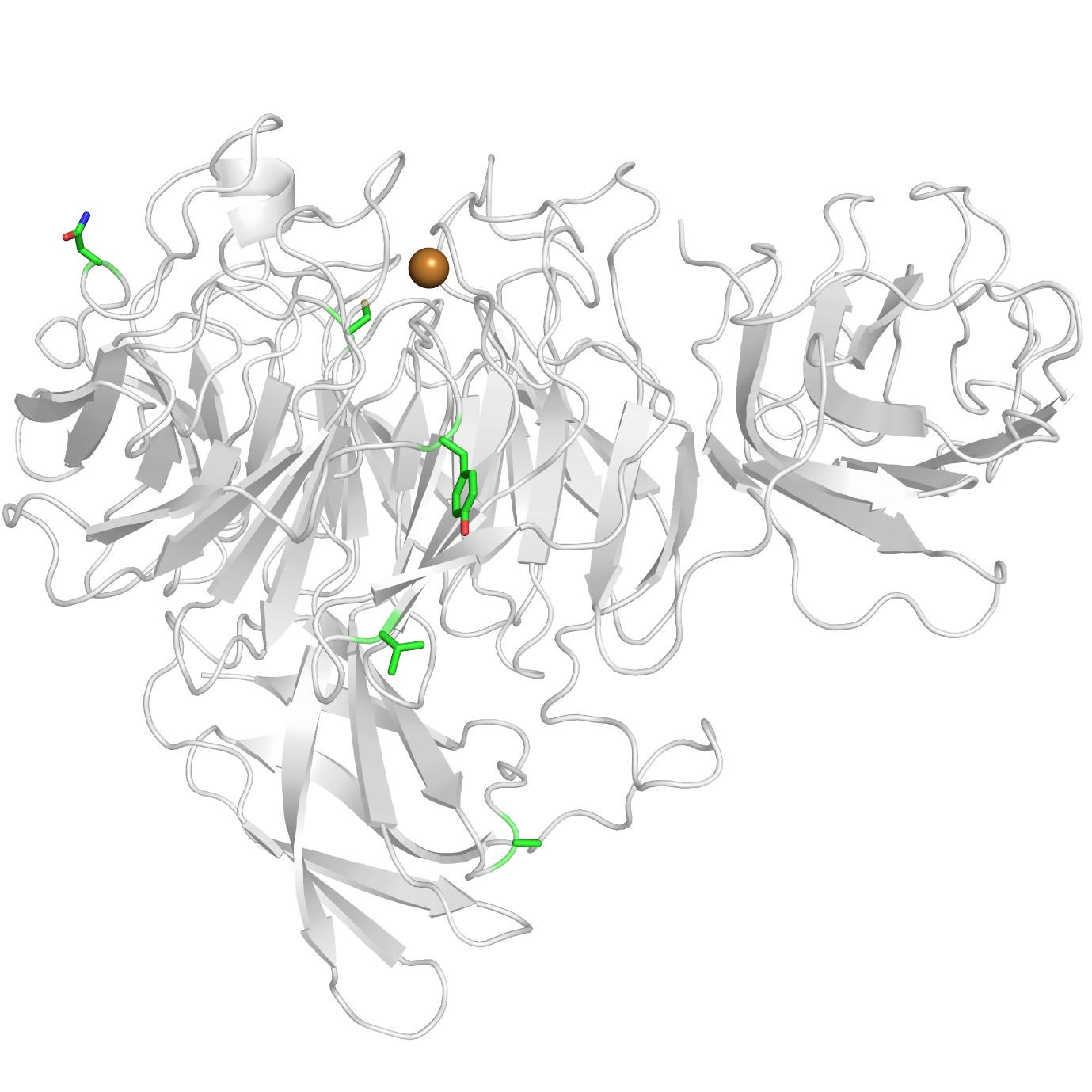


**Tyr436**

**Ala626**

**Val477**

**Cys383**

**Asn318**

**Supplementary Figure 1.** Sites targeted in the random recombination library. The positions of residues outside of the active site that were targeted in the random recombination library screen (Round 2a), highlighting their distance from the active site. Residues are shown on wildtype GOase crystal structure (PDBID: 1GOG). Mutations were N318D, C383S/T, Y436H, V477D and A626S^5-7^.

**Supplementary Figure 2.** Solvent evaporation rates. Solvent evaporation rates were calculated for initial overlay solvents used in this study. Rates were calculated using an aeration rate of 1 vvm at a reaction temperature of 20°C and the corresponding evaporation rate if the off-gas was condensed at 5°C. Abbreviation: BP, boiling point.


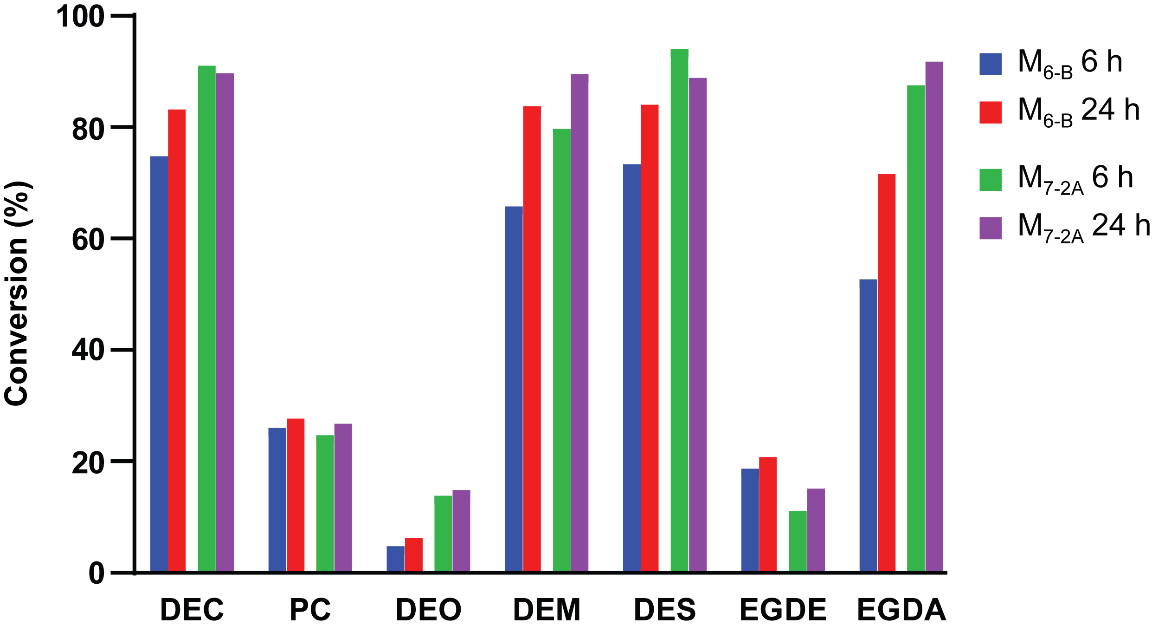


**Supplementary Figure 3.** Initial screen of new solvents for the biphasic reaction. Conversion by GOase variants using a new solvent overlay were compared at timepoints to identify which solvents were compatible with the desired reaction. Analysis performed on organic phase only after extraction (see Methods). Conditions: 0.05 g/L pure enzyme, 0.0064 g/L HRP, 880 U/mL Catalase, 100 mM NaPi, 100 g/L Crude HMF in solvent (BASF), 20°C, quenched at indicated time points. Abbreviations: DEC, diethyl carbonate. PC, propylene carbonate. DEO, diethyl oxalate. DEM, diethyl malonate. DES, diethyl succinate. EGDE, ethylene glycol diethyl ether. EGDA, ethylene glycol diacetate. Each bar represents a single experiment.

**a.**

**b.**

**Supplementary Figure 4.** Time courses measuring conversion in 0.2 L scale biotransformations. **a.** Comparison of conversion by GOase M_3-5_, M_6-A_ and M_7-2A_ reactions (Supplementary Table 17, Entries 1-3). **b.** Comparison of conversion by GOase M_7-2A_ with different catalase loadings (Supplementary Table 17, Entries 3-5). Recreated with permission from BSc thesis of M. B. M.^8^. Each datapoint represents a single analysis.

**Supplementary Figure 5.** TiTR calibration curves for HMF and DFF. Recreated with permission from PhD thesis of A. T. P.^9^.

**Supplementary Figure 6.** HPLC calibration curves for HMF, DFF, FFCA and FDCA.

**Supplementary Figure 7.** GC calibration curves for HMF and DFF.


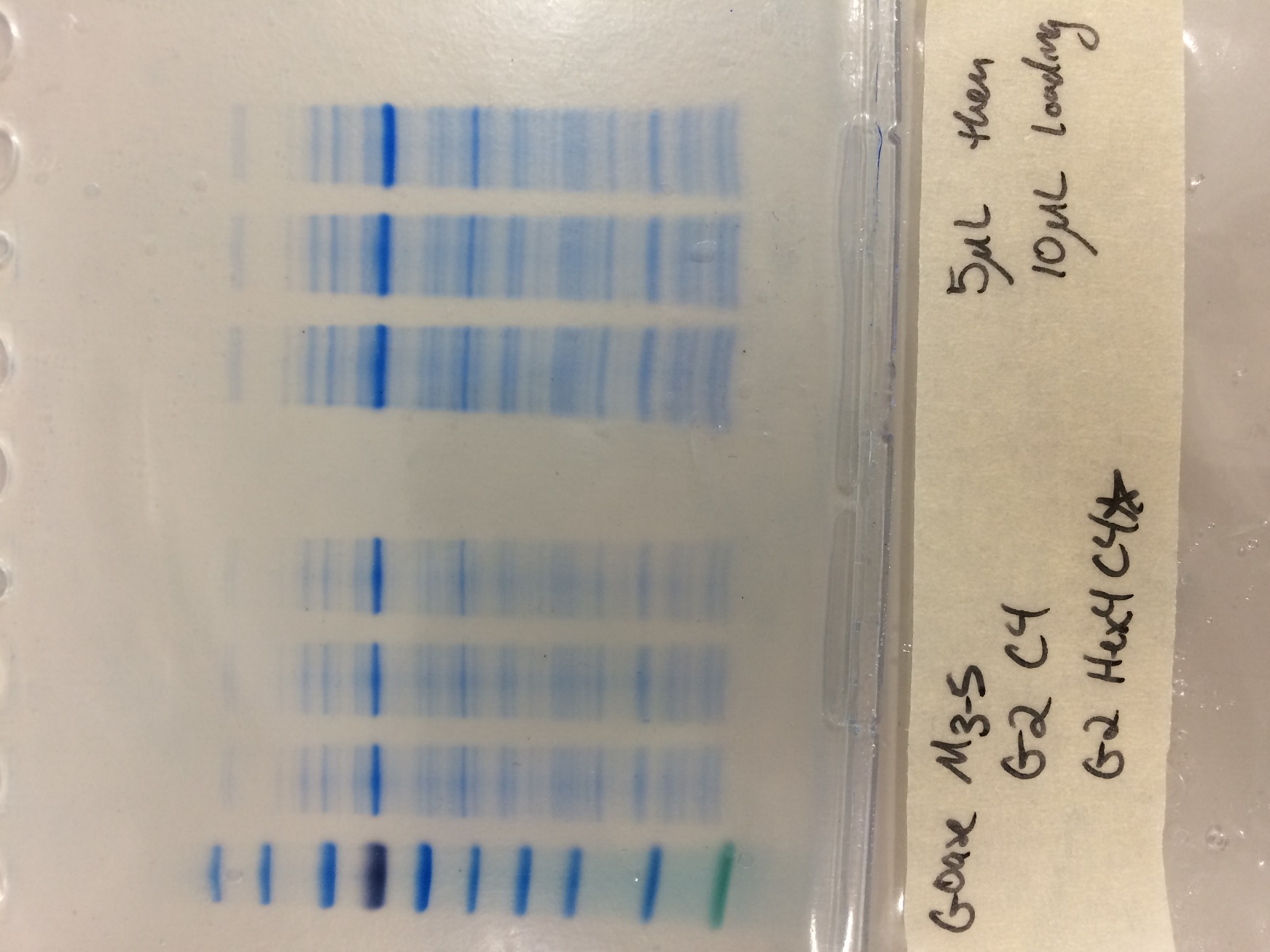


**55**

**95 kDa**

**72**

**GOase**

**Supplementary Figure 8.** SDS gel of the GOase variant CFE powder preparations used in 0.2 L scale reactions. Samples were prepared in parallel at equal stock concentrations of redissolved CFE powder and were of equal loading on the gel. Lane 1: Marker; Lane 2: GOase M_3-5_; Lane 3: GOase M_6-A_; Lane 4: Codon Optimized GOase M_7-2A_ (M_7-2A_*). CFE was prepared once for each variant, so all experiments were used with the samples shown on this gel.

# Supplementary Tables

**Supplementary Table 1.** Lineage of GOase variants

| Round | Variant Name | Mutations | Ref |
| --- | --- | --- | --- |
| Base Variant | M_1_ | S10P, M70V, P136P, G195E, V494A, N535D | Ref (^10^) |
| Base Variant | M_3_ | M_1_ + W290F, R330K, Q406T | Ref (^11^) |
| Progenitor | M_3-5_ | M_3_ + K330M | Ref (^1^) |
| R1 | M_4_ | M_3-5_ + Y329L, M330F | This work |
| R2a (Published Mutations) | M_5-1_ | M_4_ + V477D, A626S | This work |
|  | M_5-2_ | M_4_+ N318D, C383T, Y436H, V477D | This work |
| R2b (Low O_2_) | M_6-A_ | M_4_+ F290W, S291S | This work |
|  | M_6-B_ | M_4_+ F290W, S291R | This work |
| R3 (Combination) | M_7-1A_ | M_4_+ V477D, A626S + F290W, S291S | This work |
|  | M_7-2A_ | M_4_+ N318D, C383T, Y436H, V477D + F290W, S291S | This work |
|  | M_7-1B_ | M_4_+ V477D, A626S + F290W, S291R | This work |
|  | M_7-2B_ | M_4_+ N318D, C383T, Y436H, V477D + F290W, S291R | This work |

**Supplementary Table 2.** Conversion of HMF to DFF by GOase variants M_3-5_ and M_4_ under different buffer strength. Conditions listed in the table highlight the main differences between samples, while those listed below the table apply to all entries unless otherwise stated.

| Entry | GOase Variant | [NaPi] (mM) | Conversion (%) | Over-oxidation (%) |
| --- | --- | --- | --- | --- |
| 1 | M_3-5_ | 10 | 36 | 0.1 |
| 2 | M_4_ | 10 | 78 | 1.2 |
| 3 | M_3-5_ | 25 | 84 | 1.1 |
| 4 | M_4_ | 25 | 91 | 3.0 |
| 5 | M_3-5_ | 50 | 78 | 0.8 |
| 6 | M_4_ | 50 | 90 | 2.7 |
| 7 | M_3-5_ | 100 | 88 | 1.4 |
| 8 | M_4_ | 100 | 93 | 4.3 |
| 9 | M_3-5_ | 400 | 85 | 1.3 |
| 10 | M_4_ | 400 | 86 | 2.2 |

Conditions: 0.1 g/L purified enzyme, 0.0128 g/L HRP, 440 U/mL Catalase, 5% ACN, 100 mM Pure HMF, NaPi pH 7.4, 250 rpm, 25°C, 8h.

**Supplementary Table 3.** Conversion of HMF to DFF by GOase variant M_4_ in the presence of different cosolvents. Conditions listed in the table highlight the main differences between samples, while those listed below the table apply to all entries unless otherwise stated.

| **Entry** | **GOase Variant** | **Co-Solvent** | **Time (h)** | **Conversion (%)** | **Over-oxidation (%)** |
| --- | --- | --- | --- | --- | --- |
| 1 | M_4_ | None | 2 | 93 | 24.2 (8 h) |
| 2 | M_4_ | 10% DMSO | 1 | 94 | 19.1 (8 h) |

Conditions: 0.1 g/L purified enzyme, 0.0128 g/L HRP, 440 U/mL Catalase, 100 mM NaPi pH 7.4, 100 mM Pure HMF, 250 rpm, 25°C.

**Supplementary Table 4.** Conversion of HMF to DFF by GOase variant M_4_ under different enzyme and substrate loadings. Conditions listed in the table highlight the main differences between samples, while those listed below the table apply to all entries unless otherwise stated.

| **Entry** | **GOase Variant** | **GOase (g/L)** | **HRP (g/L)** | **Catalase (U/mL)** | **HMF (mM)** | **Time (h)** | **Conversion (%)** | **Over-oxidation (%)** |
| --- | --- | --- | --- | --- | --- | --- | --- | --- |
| 1 | M_4_ | 0.05 | 0.0064 | 440 | 100 | 3 | 94 | 5.1 (8 h) |
| 2 | M_4_ | 0.1 | 0.0128 | 440 | 100 | 1 | 94 | 19.1 (8 h) |
| 3 | M_4_ | 0.05 | 0.0064 | 880 | 250 | 8 | 71 | 1.0 (8 h) |
| 4 | M_4_ | 0.1 | 0.0128 | 880 | 250 | 8 | 93 | 2.2 (8 h) |
| 5 | M_4_ | 0.05 | 0.0064 | 880 | 500 | 8 | 30 | 0.4 (8 h) |
| 6 | M_4_ | 0.1 | 0.0128 | 880 | 500 | 8 | 33 | 0.7 (8 h) |

Conditions: Purified GOase M_4_, 100 mM NaPi, 10% DMSO, Pure HMF, 25°C.

**Supplementary Table 5.** Conversion of HMF to DFF by GOase variant M_4_ using crude HMF preparation. Conditions listed in the table highlight the main differences between samples, while those listed below the table apply to all entries unless otherwise stated.

| Entry | GOase Variant | GOase (g/L) | HRP (g/L) | [HMF] | HMF Form | Conversion (%) | Over-oxidation (%) |
| --- | --- | --- | --- | --- | --- | --- | --- |
| 1 | M_4_ | 0.05 | 0.0064 | 25 g/L | Crude | 71 | 1.0 |
| 2 | M_4_ | 0.1 | 0.0128 | 25 g/L | Crude | 86 | 3.3 |
| 3 | M_4_ | 0.05 | 0.0064 | 50 g/L | Crude | 29 | 0.0 |
| 4 | M_4_ | 0.1 | 0.0128 | 50 g/L | Crude | 44 | 0.5 |

Conditions: Purified GOase M_4_, 880 U/mL Catalase, 100 mM NaPi, 10% DMSO, Crude HMF, 25°C, 8 h.

**Supplementary Table 6.** Conversion of HMF to DFF by GOase variant M_4_ in purified and CFE formulations. Conditions listed in the table highlight the main differences between samples, while those listed below the table apply to all entries unless otherwise stated.

| Entry | GOase Variant | GOase (g/L) | GOase Form | HRP (g/L) | [HMF] | HMF Form | Conversion (%) | Over-oxidation (%) 8h |
| --- | --- | --- | --- | --- | --- | --- | --- | --- |
| 1 | M_4_ | 0.05 | Purified | 0.0064 | 250 mM | Pure | 50 | 0.7 |
| 2 | M_4_ | 0.625 | CFE | 0.0128 | 250 mM | Pure | 66 | 1.4 |
| 3 | M_4_ | 0.05 | Purified | 0.0064 | 500 mM | Pure | 28 | 0.2 |
| 4 | M_4_ | 0.625 | CFE | 0.0128 | 500 mM | Pure | 30 | 0.6 |

Conditions: 0.05 mM CuSO_4_ in GOase M_4_ CFE reactions, 880 U/mL Catalase, 100 mM NaPi, No Cosolvent, Pure HMF, 25°C, 8 h.

**Supplementary Table 7.** Conversion of HMF to DFF by GOase variant M_4_ in the presence of different cosolvents. Conditions listed in the table highlight the main differences between samples, while those listed below the table apply to all entries unless otherwise stated.

| Entry | GOase Variant | Co-Solvent | Time (h) | Conversion (%) | Over-oxidation (%) |
| --- | --- | --- | --- | --- | --- |
| 1 | M_4_ | 10% DMSO | 8 | 71 | 1.0 |
| 2 | M_4_ | None | 8 | 50 | 0.7 |
| 3 | M_4_ | 10% THF | 6 | 24 | 0.1 |
| 4 | M_4_ | 10% Me-THF | 6 | 28 | 0.2 |
| 5 | M_4_ | 10% Acetone | 8 | 54 | 0.7 |
| 6 | M_4_ | 10% Isopropanol | 8 | 49 | 1.3 |
| 7 | M_4_ | 10% Butanone | 8 | 33 | 0.5 |
| 8 | M_4_ | 40 + 40% tBME^a^ | 8 | 62 | 1.2 |
| 9 | M_4_ | 40 + 40% EtOAc^a^ | 8 | 75 | 1.0 |
| 10 | M_4_ | 40 + 40% Toluene^a^ | 8 | 52 | 0.7 |

Conditions: 0.05 g/L Purified GOase M_4_, 0.0064 g/L HRP, 880 U/mL Catalase, 100 mM NaPi, 250 mM Pure HMF, 25°C. **^a^**40 + 40% is 100 µL solvent added at start and 100 µL added after 4 h.

**Supplementary Table 8.** Conversion of HMF to DFF by GOase variant M_4_ as purified enzyme and CFE using EtOAc overlay. Conditions listed in the table highlight the main differences between samples, while those listed below the table apply to all entries unless otherwise stated.

| Entry | GOase Variant | GOase (g/L) | GOase Form | HRP (g/L) | [HMF] | HMF Form | Conversion (%) | Over-oxidation (%) 6 h |
| --- | --- | --- | --- | --- | --- | --- | --- | --- |
| 1 | M_4_ | 0.05 | Purified | 0.0064 | 250 mM | Pure | 88 | 0.9 |
| 2 | M_4_ | 0.625 | CFE | 0.0128 | 250 mM | Pure | 96 | 1.6 |
| 3 | M_4_ | 0.05 | Purified | 0.0064 | 25 g/L | Crude | 87 | 1.3 |
| 4 | M_4_ | 0.625 | CFE | 0.0128 | 25 g/L | Crude | 90 | 4.5 |

Conditions: 0.05 mM CuSO_4_ in GOase M_4_ CFE reactions, 880 U/mL Catalase, 100 mM NaPi, 200 µL (80% of aqueous volume) EtOAc, 25°C, 6 h.

**Supplementary Table 9.** Conversion of HMF to DFF by GOase variants M_4_ at different reaction temperatures. Conditions listed in the table highlight the main differences between samples, while those listed below the table apply to all entries unless otherwise stated.

| Entry | GOase Variant | Temp (°C) | Time (h) | Conversion (%) | Over-oxidation (%) |
| --- | --- | --- | --- | --- | --- |
| 1 | M_4_ | 25 | 8 | 50 | 0.7 |
| 2 | M_4_ | 20 | 6 | 53 | 0.7 |
| 3 | M_4_ | 30 | 8 | 49 | 0.5 |
| 4 | M_4_ | 40 | 8 | 28 | 0.7 |

Conditions: 0.05 g/L Purified enzyme, 0.0064 g/L HRP, 880 U/mL Catalase, 100 mM NaPi, No Cosolvent, 250 mM Pure HMF.

**Supplementary Table 10.** Conversion of HMF to DFF by GOase variant M_4_ as purified enzyme and CFE using EtOAc overlay at reduced incubation temperature. Conditions listed in the table highlight the main differences between samples, while those listed below the table apply to all entries unless otherwise stated.

| Entry | GOase Variant | GOase (g/L) | GOase Form | HRP (g/L) | [HMF] | HMF Form | Conversion (%) | Over-oxidation (%) |
| --- | --- | --- | --- | --- | --- | --- | --- | --- |
| 1 | M_4_ | 0.05 | Purified | 0.0064 | 250 mM | Pure | 95 | 1.5 |
| 2 | M_4_ | 0.625 | CFE | 0.0128 | 250 mM | Pure | 96 | 2.5 |
| 3 | M_4_ | 0.05 | Purified | 0.0064 | 25 g/L | Crude | 91 | 2.1 |
| 4 | M_4_ | 0.625 | CFE | 0.0128 | 25 g/L | Crude | 88 | 6.4 |
| 5 | M_4_ | 0.05 | Purified | 0.0064 | 500 mM | Pure | 56 | 0.1 |
| 6 | M_4_ | 0.625 | CFE | 0.0128 | 500 mM | Pure | 71 | 0.4 |
| 7 | M_4_ | 0.05 | Purified | 0.0064 | 50 g/L | Crude | 64 | 0.3 |
| 8 | M_4_ | 0.625 | CFE | 0.0128 | 50 g/L | Crude | 82 | 0.8 |

Conditions: 0.05 mM CuSO_4_ in GOase M_4_ CFE reactions, 880 U/mL Catalase, 100 mM NaPi, 200 µL (80% of aqueous volume) EtOAc, 20°C, 6 h.

**Supplementary Table 11.** Conversion of HMF to DFF by GOase variant M_4_ as purified enzyme and CFE at high HMF loading in the presence of different cosolvents. Conditions listed in the table highlight the main differences between samples, while those listed below the table apply to all entries unless otherwise stated.

| Entry | GOase Variant | GOase (g/L) | GOase Form | HRP (g/L) | Co-Solvent | [HMF] | HMF Form | Conversion (%) | Over-oxidation (%) |
| --- | --- | --- | --- | --- | --- | --- | --- | --- | --- |
| 1 | M_4_ | 0.05 | Purified | 0.0064 | EtOAc | 250 mM | Pure | 95 | 1.5 |
| 2 | M_4_ | 0.05 | Purified | 0.0064 | DEC | 250 mM | Pure | 97 | 0.8 |
| 3 | M_4_ | 0.05 | Purified | 0.0064 | BuOAc | 250 mM | Pure | 93 | 0.6 |
| 4 | M_4_ | 0.05 | Purified | 0.0064 | EtOAc | 500 mM | Pure | 56 | 0.1 |
| 5 | M_4_ | 0.05 | Purified | 0.0064 | DEC | 500 mM | Pure | 54 | 0.2 |
| 6 | M_4_ | 0.05 | Purified | 0.0064 | BuOAc | 500 mM | Pure | 50 | 0.2 |
| 7 | M_4_ | 0.625 | CFE | 0.0128 | EtOAc | 50 g/L | Crude | 82 | 0.8 |
| 8 | M_4_ | 0.625 | CFE | 0.0128 | DEC | 50 g/L | Crude | 82 | 0.8 |
| 9 | M_4_ | 0.625 | CFE | 0.0128 | BuOAc | 50 g/L | Crude | 76 | 0.7 |

Conditions: 0.05 mM CuSO_4_ in GOase M_4_ CFE reactions, 880 U/mL Catalase, 100 mM NaPi, 200 µL (80% of aqueous volume) solvent overlay, 20°C, 6 h.

**Supplementary Table 12.** Phase partitioning of HMF and DFF in biphasic systems with equal volumes of solvent and 100 mM NaPi pH 7.4.

| Solvent System | HMF | DFF |
| --- | --- | --- |
| Log P (EtOAc/H_2_O) | 0.08 | 0.58 |
| Log P (DEC/H_2_O) | -0.27 | 0.47 |
| Log P (BuOAc/H_2_O) | -0.22 | 0.37 |

Log P (Oct/H_2_O) of the solvents: EtOAc: 0.73, DEC: 1.21, BuOAc: 1.82. Abbreviations: EtOAc, ethyl acetate. BuOAc, butyl acetate. DEC, diethyl carbonate.

**Supplementary Table 13.** Conversion of 50 g/L semi-crude HMF to DFF by GOase variants. Conditions listed in the table highlight the main differences between samples, while those listed below the table apply to all entries unless otherwise stated.

| Entry | GOase Variant | Time (h) | Conversion (%) | Over-oxidation (%) |
| --- | --- | --- | --- | --- |
| 1 | M_4_ | 2 | 43 | 0.1 |
| 2 | M_5-1_ | 2 | 56 | 0.2 |
| 3 | M_5-2_ | 2 | 57 | 0.2 |
| 4 | M_6-A_ | 2 | 67 | 0.0 |
| 5 | M_6-B_ | 2 | 56 | 0.1 |
|  |  |  |  |  |
| 6 | M_4_ | 6 | 68 | 0.4 |
| 7 | M_5-1_ | 6 | 78 | 0.8 |
| 8 | M_5-2_ | 6 | 85 | 1.6 |
| 9 | M_6-A_ | 6 | 89 | 0.6 |
| 10 | M_6-B_ | 6 | 86 | 2.2 |

Conditions: 0.05 g/L pure enzyme, 0.0064 g/L HRP, 880 U/mL Catalase, 100 mM NaPi, 50 g/L Crude HMF in DEC (BASF), 200 µL (80% of aqueous volume) DEC, 20°C, 2 and 6 h.

**Supplementary Table 14.** Physical properties of key water immiscible solvents used within this work.

|  | EtOAc | BuOAc | DEC | PC | DEO | DEM | DES | EGDE | EGDA |
| --- | --- | --- | --- | --- | --- | --- | --- | --- | --- |
| Log P | 0.71 | 1.82 | 1.21 | -0.4^b^ | 0.73 | 0.7^b^ | 1.26^b^ | 0.6^b^ | -0.05^b^ |
| Density (g/mL) | 0.9 | 0.88 | 0.98 | 1.21 | 1.08 | 1.05 | 1.05 | 0.84 | 1.13 |
| H_2_O Solubility (g/L) | 83 | 6.8 | Insol | 240 | Insol/Decomp | Neg | 20 | 34 | 160 |
| Boiling Point (°C) | 77 | 126 | 126 | 242 | 185 | 199 | 216 | 119 | 186 |

Abbreviations: EtOAc, ethyl acetate. BuOAc, butyl acetate. DEC, diethyl carbonate. PC, propylene carbonate. DEO, diethyl oxalate. DEM, diethyl malonate. DES, diethyl succinate. EGDE, ethylene glycol diethyl ether. EGDA, ethylene glycol diacetate. Insol, insoluble. Decomp, possible decomposition. Neg, negligible. ^b^LogP predicted by ACD Labs via ChemSpider.

**Supplementary Table 15.** Conversion of 100 and 150 g/L semi-crude HMF to DFF by GOase variant M_7-2A_. Conditions listed in the table highlight the main differences between samples, while those listed below the table apply to all entries unless otherwise stated.

| Entry | GOase Variant | GOase (g/L) | Co-Solvent | [HMF] | HMF Form | Time (h) | Conversion (%) |
| --- | --- | --- | --- | --- | --- | --- | --- |
| 1 | M_7-2A_ | 0.1 | DEC | 100 g/L | Crude | 6 | 96 |
| 2 | M_7-2A_ | 0.1 | DEC | 150 g/L | Crude | 6 | 62 |
| 3 | M_7-2A_ | 0.1 | DEC | 100 g/L | Crude | 24 | 96 |
| 4 | M_7-2A_ | 0.1 | DEC | 150 g/L | Crude | 24 | 66 |

Conditions: 0.1 g/L pure enzyme, 0.0128 g/L HRP, 880 U/mL Catalase, 100 mM NaPi, 100 or 150 g/L Crude HMF in DEC (BASF), 200 µL (80% of aqueous volume) DEC, 20°C, 6 or 24 h. Less than 1% over-oxidation, if any, was observed in these samples.

**Supplementary Table 16.** Conditions and conversions for 10 mL scale biotransformations. Conditions listed in the table highlight the main differences between samples, while those listed below the table apply to all entries unless otherwise stated.

| **Entry** | **GOase Variant** | **GOase (g/L)** | **GOase Form** | **HRP (g/L)** | **Co-Solvent** | **[HMF]**  **(g/L)** | **HMF Form** | **Temp (°C)** | **Time (h)** | **Conversion (%)** | **Over-oxidation (%)** |
| --- | --- | --- | --- | --- | --- | --- | --- | --- | --- | --- | --- |
| 1 | M_4_ | 0.1 | Purified | 0.0128 | 10% DMSO | 25 | Pure | 25 | 8 | 94 | 2.5 |
| 2 | M_4_ | 0.1 | Purified | 0.0128 | 10% DMSO | 25 | Crude | 25 | 8 | 80 | 1.5 |
| 3 | M_4_ | 0.05 | Purified | 0.0064 | 80% EtOAc | 25 | Pure | 20 | 6 | 96 | 1.7 |
| 4 | M_4_ | 0.625 | CFE | 0.0128 | 80% EtOAc | 25 | Pure | 20 | 6 | 97 | 2.4 |
| 5 | M_4_ | 0.05 | Purified | 0.0064 | 80% EtOAc | 25 | Crude | 20 | 6 | 88 | 1.6 |
| 6 | M_4_ | 0.625 | CFE | 0.0128 | 80% EtOAc | 25 | Crude | 20 | 6 | 90 | 4.1 |
| 7 | M_4_ | 0.625 | CFE | 0.0128 | 80% EtOAc | 50 | Pure | 20 | 24 | 78 | 0.6 |
| 8 | M_4_ | 0.625 | CFE | 0.0128 | 80% EtOAc | 50 | Crude | 20 | 24 | 80 | 0.8 |

Conditions: All reactions were performed with 880 U/mL catalase and were in 100 mM NaPi. All reactions with CFE contained 0.05 mM CuSO_4_.

**Supplementary Table 17.** Conditions and conversions for 0.2 L scale reactions. Conditions listed in the table highlight the main differences between samples, while those listed below the table apply to all entries unless otherwise stated.

| **Entry** | **GOase Variant** | **GOase (g/L)** | **GOase Form** | **Catalase (U/mL)** | **[HMF]**  **(g/L)** | **Aeration (vvm air)** | **Conversion (%)** | **Volumetric Productivity (g_DFF_/L•h)** | **Biocatalyst Productivity (g_DFF_/g_biocatalyst_) (g_DFF_/g_pure enz eq_)** |
| --- | --- | --- | --- | --- | --- | --- | --- | --- | --- |
| 1 | M_3-5_ | 0.9 | CFE | 48 | 50 | 0.09 | 24 | 1.2 | 12.9 (232) |
| 2 | M_6-A_ | 1.57 | CFE | 48 | 50 | 0.09 | 37 | 1.7 | 10.6 (333) |
| 3 | M_7-2A_ | 0.95 | CFE | 51 | 50 | 0.09 | 36 | 1.7 | 17.7 (336) |
| 4 | M_7-2A_ | 0.96 | CFE | 162 | 50 | 0.09 | 49 | 2.2 | 23.5 (451) |
| 5 | M_7-2A_ | 0.95 | CFE | 305 | 50 | 0.09 | 59 | 2.6 | 27.3 (519) |

Conditions: Pure HMF, 0.05 mM CuSO_4_, 4 U/mL HRP, 100 mM NaPi pH 7.4, 20°C, 6 h, stirring speed 500 rpm. 110 mL aqueous volume with 90 mL DEC overlay. The amount of CFE added was adjusted to load equivalent unit activity compared to the analytical scale reactions with purified enzyme. Calculation of biocatalyst productivity with respect to this pure enzyme equivalent is given in parentheses in the final column for more direct comparison to the analytical scale reactions. Data previously published in the BSc thesis of M. B. M.^8^.

**Supplementary Table 18.** Specific activities of GOase CFE preps used for 0.2 L reactions. Activity is normalized to mg CFE. *Denotes codon optimized gene used for CFE preparation.

| GOase Variant | Specific Activity  (µmol/(min•mg CFE)) |
| --- | --- |
| M_3-5_ | 5.14 ± 0.10 |
| M_6-A_ | 4.33 ± 0.16 |
| M_7-2A_* | 6.01 ± 0.39 |

# Sequence Information

**Codon Optimized GOase M_7-2A_**

ATGGCGTCTGCACCTATTGGTAGTGCGATCCCACGCAACAATTGGGCAGTAACATGCGATTCCGCTCAATCAGGCAATGAGTGCAACAAGGCTATTGACGGGAATAAAGACACTTTTTGGCATACATTCTATGGGGCAAATGGCGATCCGAAGCCACCTCATACCTATACCATTGATATGAAGACCACGCAGAATGTGAATGGACTGTCGGTGTTACCTCGTCAGGACGGTAATCAAAATGGCTGGATCGGCCGCCATGAAGTGTACTTGTCCTCAGATGGAACCAACTGGGGTAGCCCAGTCGCGAGCGGCAGCTGGTTTGCAGATTCGACAACGAAGTACTCTAACTTTGAAACCCGTCCGGCACGTTACGTTCGTCTTGTAGCCATTACGGAGGCTAATGGCCAACCTTGGACCTCTATTGCCGAAATTAATGTCTTCCAAGCTAGCTCTTACACCGCCCCACAGCCGGGATTGGGTCGTTGGGGCCCGACAATCGACCTTCCAATCGTCCCCGCGGCCGCAGCAATCGAGCCCACATCCGGCCGTGTTTTAATGTGGTCGAGCTACCGTAATGACGCATTTGAGGGTTCCCCTGGGGGGATCACCCTGACGTCTTCATGGGACCCGTCAACTGGAATCGTCTCAGATCGTACAGTGACAGTTACTAAGCATGACATGTTCTGCCCAGGTATCAGCATGGACGGGAATGGGCAGATCGTGGTTACGGGCGGTAACGATGCTAAGAAGACGAGTCTGTATGACAGTTCATCAGACAGTTGGATCCCTGGCCCCGACATGCAAGTAGCACGCGGTTACCAGTCCTCTGCGACTATGTCCGACGGCCGTGTCTTTACAATCGGGGGGTCATGGAGTGGGGGAGTATTTGAGAAAAACGGAGAGGTTTATTCGCCTTCCTCCAAGACCTGGACTTCTTTACCGAATGCAAAAGTAGACCCGATGTTGACCGCCGATAAACAAGGCTTACTTTTTTCCGATAATCATGCCTGGTTATTTGGTTGGAAGAAGGGATCTGTATTTCAAGCAGGTCCCAGCACGGCGATGAACTGGTACTATACGTCAGGATCGGGAGACGTCAAATCTGCAGGAAAACGCCAATCAAACCGCGGGGTTGCACCTGACGCAATGACCGGAAATGCAGTGATGTATGACGCAGTGAAGGGCAAAATCCTGACCTTTGGTGGTTCGCCCGACTACACAGACAGCGATGCGACAACAAATGCCCACATTATTACACTTGGCGAACCCGGTACATCCCCCAATACGGTATTTGCTTCAAATGGGTTGCACTTCGCGCGTACCTTCCATACTTCGGTGGTCTTACCAGATGGCAGTACCTTTATTACCGGTGGGCAGCGTCGTGGGATTCCGTTTGAAGATTCGACACCCGTTTTTACACCGGAGATTTATGACCCGGAGCAGGACACCTTTTACAAGCAAAACCCAAATAGCATCGTCCGCGCTTATCATAGCATCAGCTTGTTATTGCCAGATGGGCGTGTGTTTAATGGCGGCGGAGGTTTGTGCGGAGATTGCACGACGAACCACTTCGACGCACAAATCTTTACTCCGAACTACCTGTATGACAGCAATGGGAACCTGGCAACCCGTCCCAAGATTACGCGCACTTCTACACAATCGGTCAAGGTCGGAGGGCGTATTACCATTTCTACTGATTCAAGTATTTCTAAAGCGAGTTTAATTCGTTACGGGACCGCTACGCATACCGTCAATACGGATCAACGCCGCATTCCACTTACGCTTACAAACAACGGTGGAAACAGTTATTCTTTTCAAGTGCCTAGCGACTCTGGGGTTGCTCTGCCCGGGTACTGGATGCTTTTCGTGATGAACAGTGCGGGCGTTCCTTCAGTTGCCAGTACTATCCGCGTTACCCAGGGCGGGGGAGGATCTTGGTCGCATCCTCAGTTTGAGAAATAA

**Supplementary Table 19.** Mutagenesis primers used in this work. Sites of mutation are indicated in red.

|  | Primer Sequence |
| --- | --- |
| Low O_2_ Libraries |  |
| Lib C 290/291 for | ccattggaggctccNNKNNKggtggcgtatttgagaagaatgg |
| Lib C 290/291 rev | ccattcttctcaaatacgccaccMNNMNNggagcctccaatgg |
| Lib D 194/195 for | gtcttcatatcgcaatgatgcaNNKNNKggatcccctggtgg |
| Lib D 194/195 rev | ccaccaggggatccMNNMNNtgcatcattgcgatatgaagac |
| Lib E 463/464 for | ggccaacgacgtggaattNNKNNKgaggattcaaccccgg |
| Lib E 463/464 rev | ccggggttgaatcctcMNNMNNaattccacgtcgttggcc |
|  |  |
| MultiSite Mutagenesis |  |
| Asn318Asp for | cccaatgccaaggtcgacccaatgttgacgg |
| Cys383Ser for | ccctgatgccatgagcggaaacgctgtc |
| Cys383Thr for | ccctgatgccatgaccggaaacgctgtcatgtacg |
| Tyr436His for | gctagcaatgggttgcactttgcccgaacg |
| Val477Asp for | cacctgagatctacgaccctgaacaagacac |
| Ala626Ser for | cgtgatgaactcgtccggtgttcctagtgtg |
|  |  |
| Combining Variants |  |
| Add M6-A Mut for | ggaggctcctggagtggtggcgtatttgag |
| Add M6-A Mut rev | ctcaaatacgccaccactccaggagcctcc |
| Add M6-B Mut for | gaggctcctggaggggtggcgtatttgag |
| Add M6-B Mut rev | ctcaaatacgccacccctccaggagcctc |

# Representative Chromatograms

The following data is presented in Table 6:


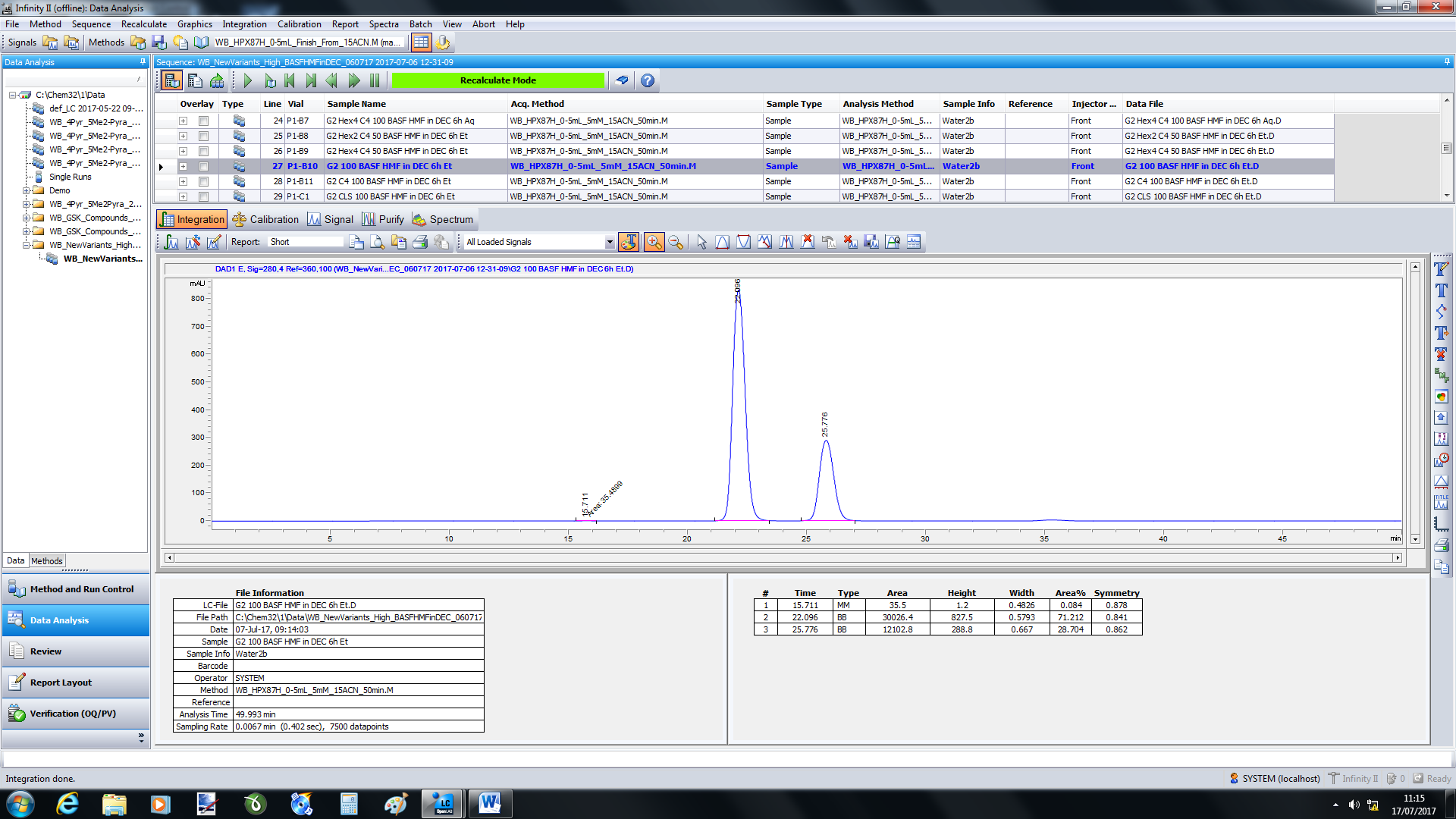


**FFCA**

**HMF**

**DFF**

M_4_ 100 BASF HMF in DEC 6h (EtOAc phase)

Table 6 Entry 1


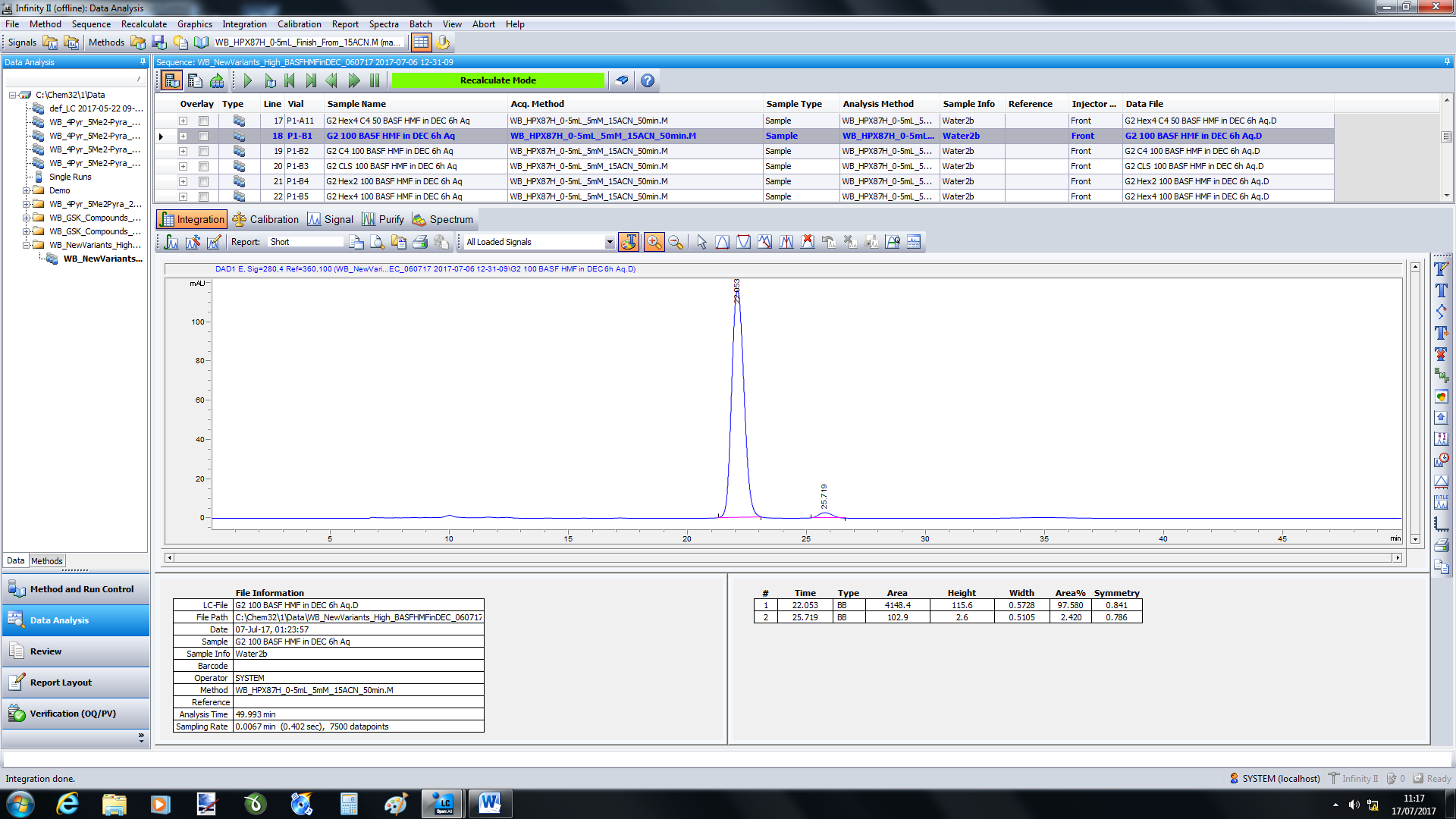


M_4_ 100 BASF HMF in DEC 6h (Aqueous phase)

Table 6 Entry 1


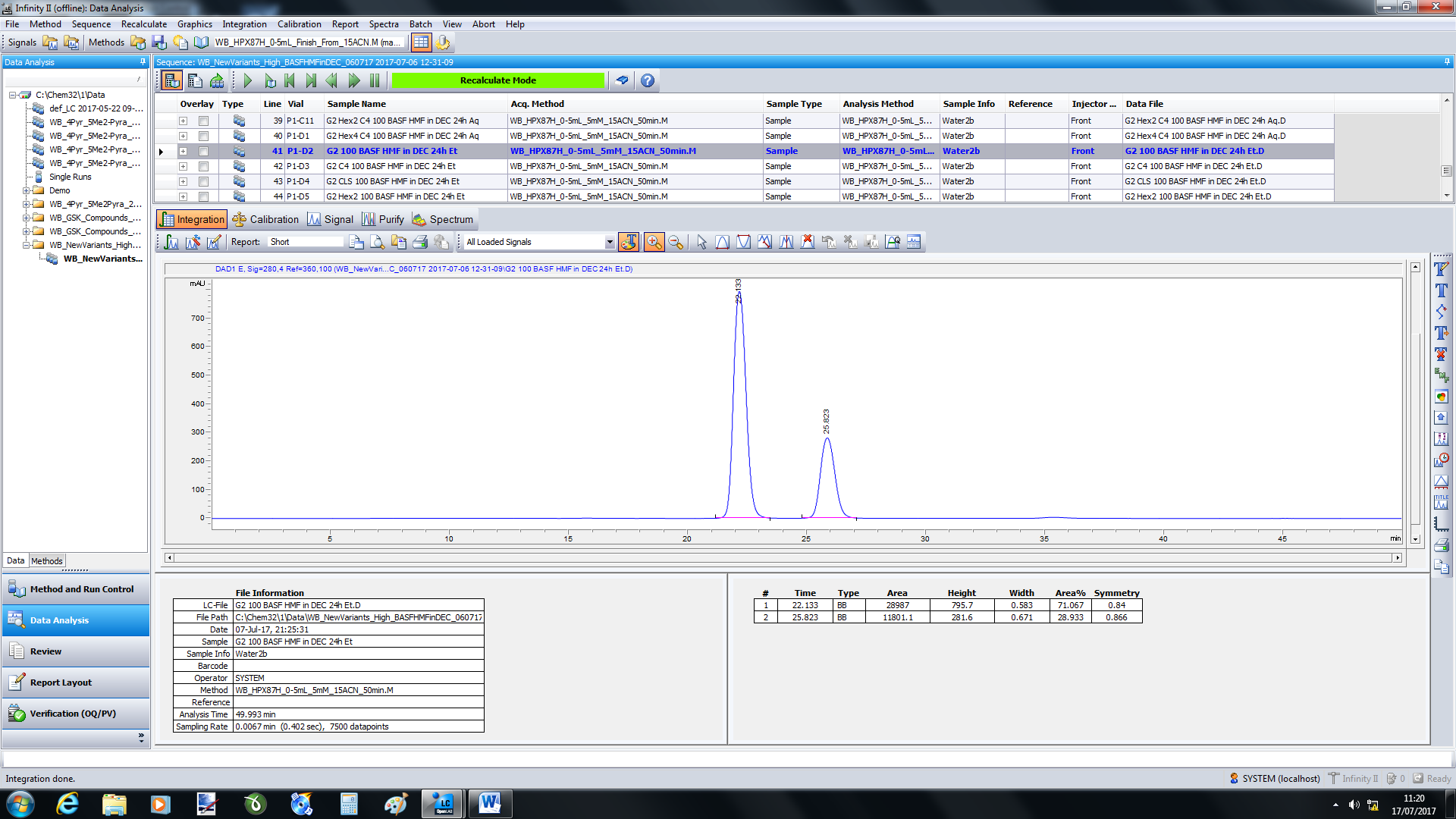


M_4_ 100 BASF HMF in DEC 24h (EtOAc phase)

Table 6 Entry 2


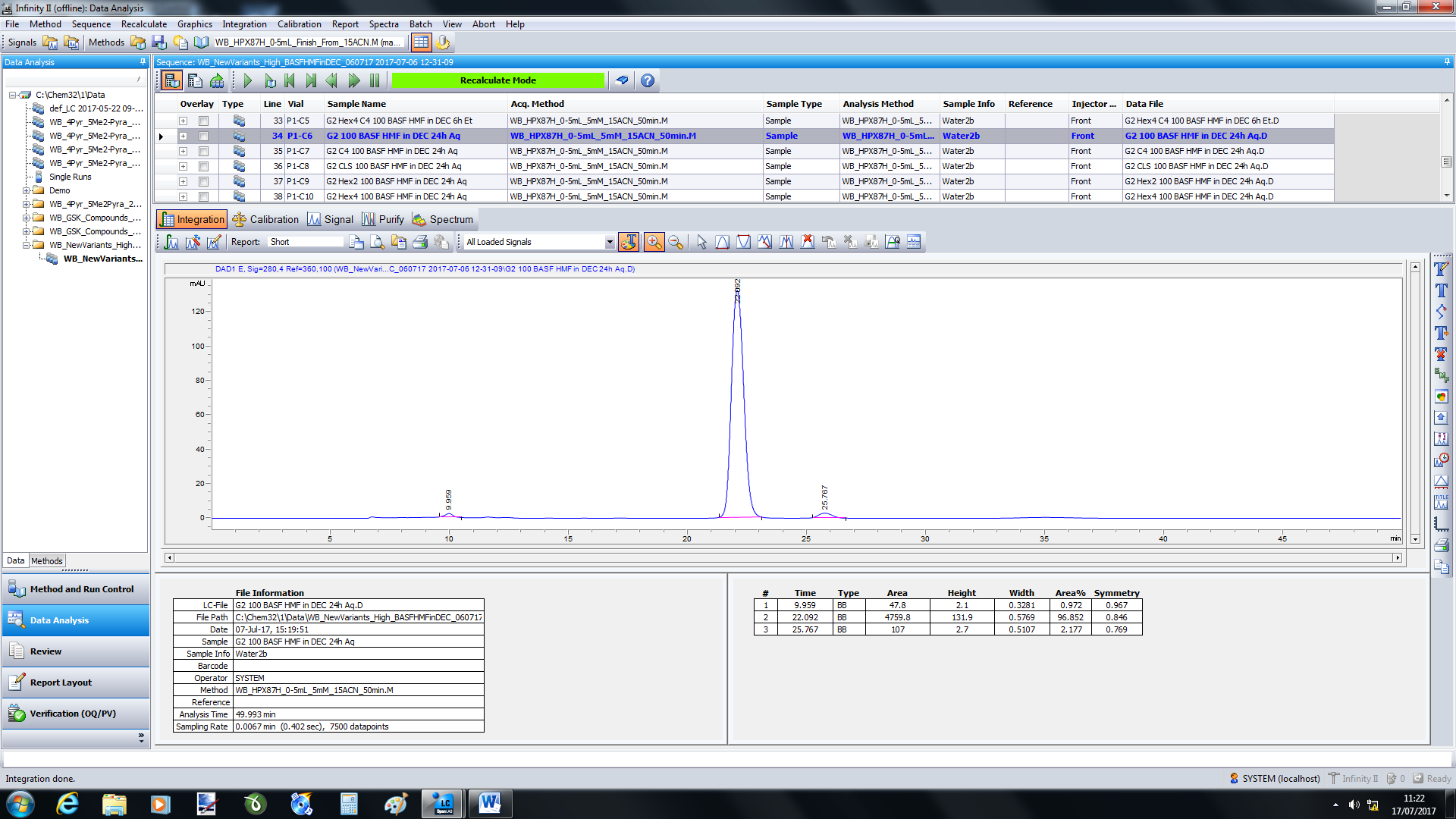


M_4_ 100 BASF HMF in DEC 24h (Aqueous phase)

Table 6 Entry 2


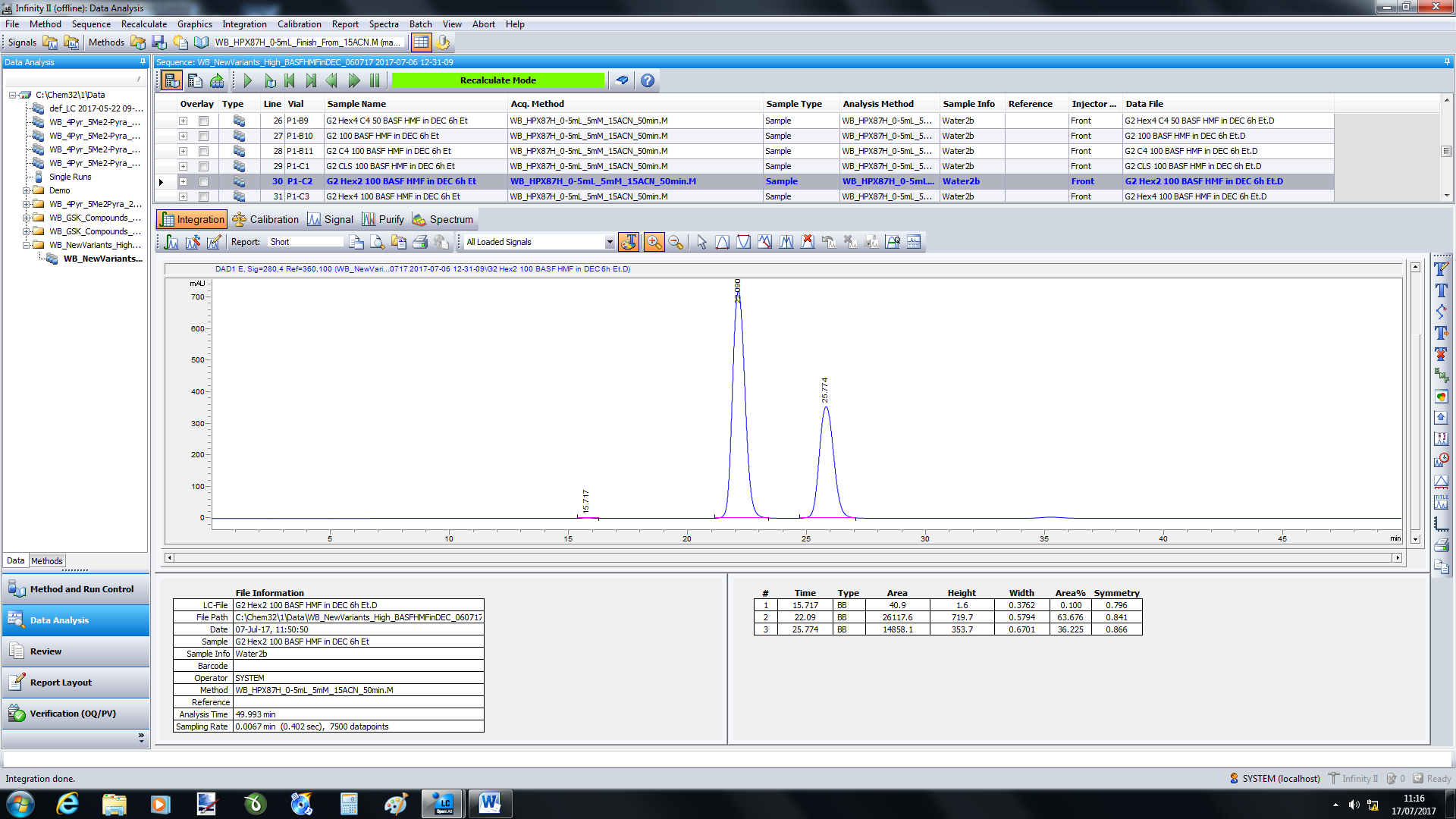


M_5-1_ 100 BASF HMF in DEC 6h (EtOAc phase)

Table 6 Entry 3


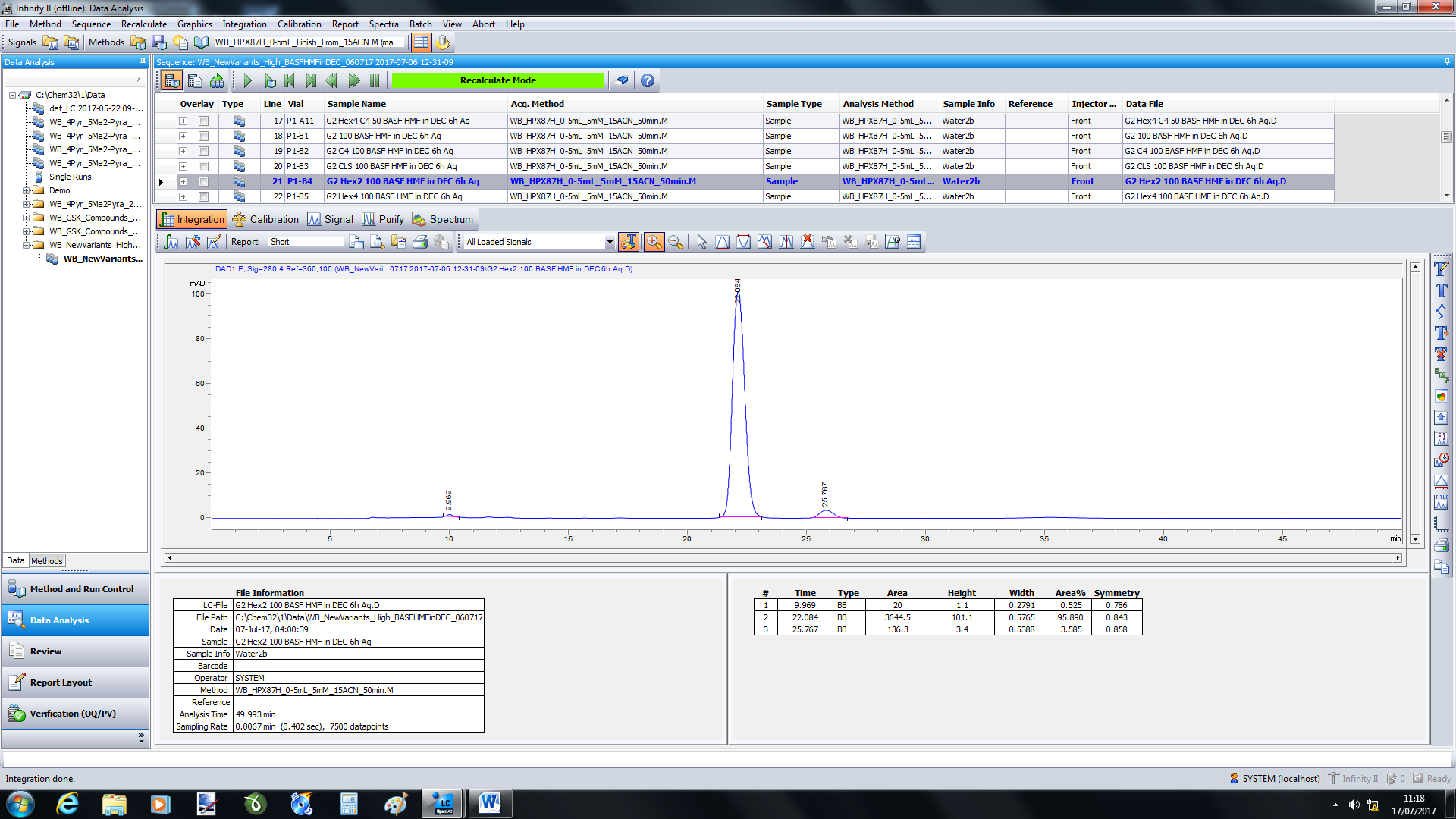


M_5-1_ 100 BASF HMF in DEC 6h (Aqueous phase)

Table 6 Entry 3


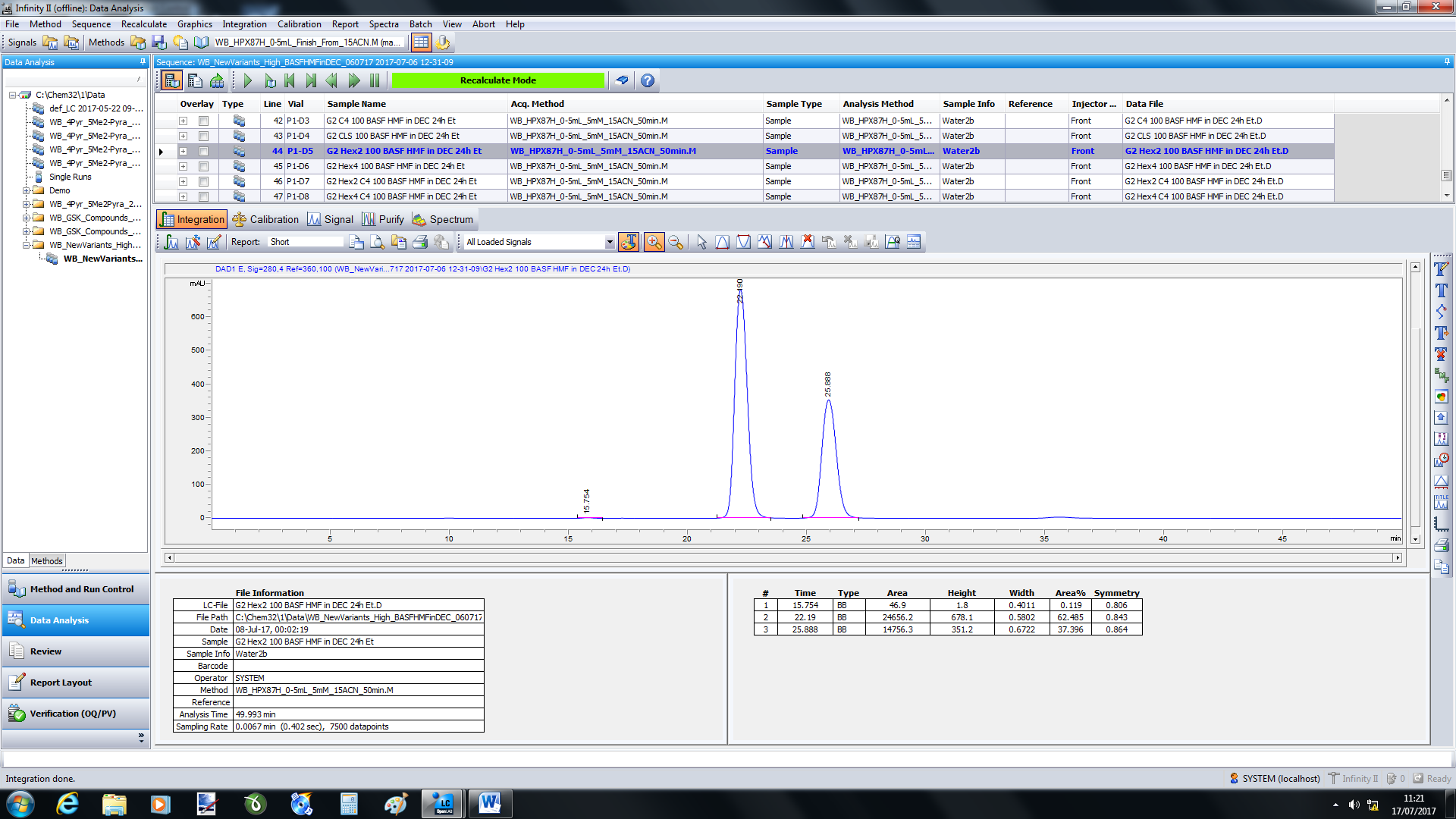


M_5-1_ 100 BASF HMF in DEC 24h (EtOAc phase)

Table 6 Entry 4


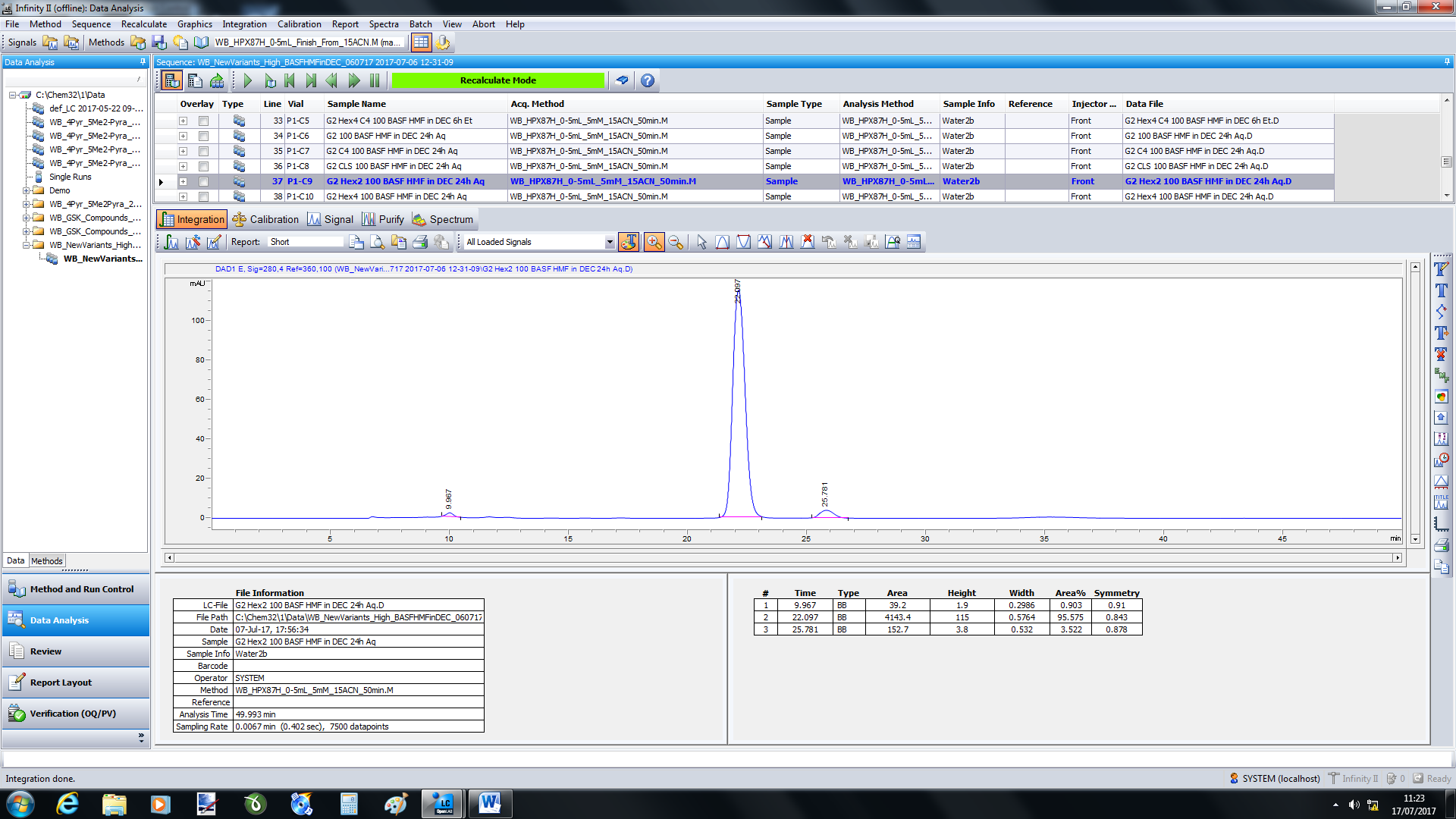


M_5-1_ 100 BASF HMF in DEC 24h (Aqueous phase)

Table 6 Entry 4


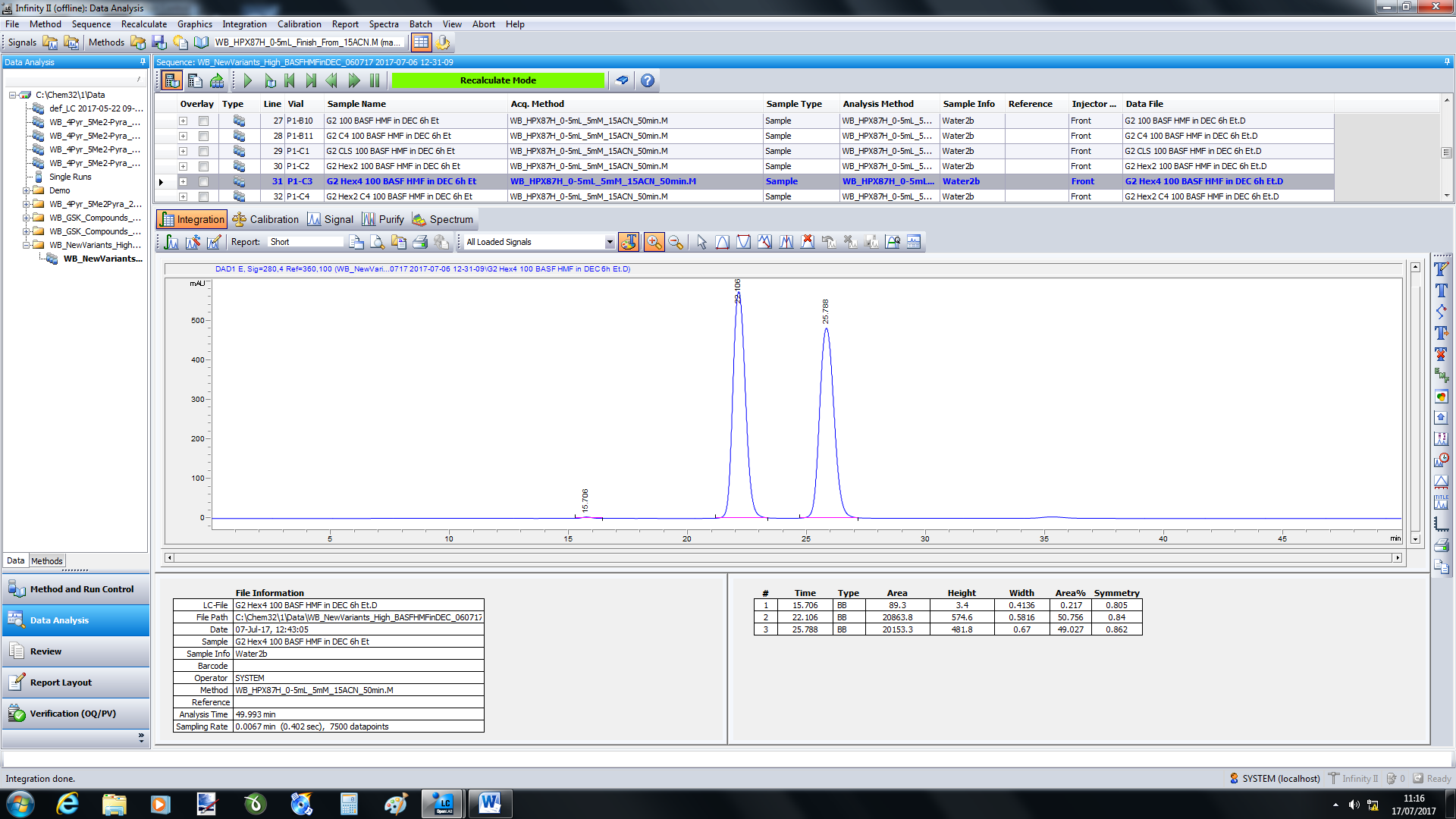


M_5-2_ 100 BASF HMF in DEC 6h (EtOAc phase)

Table 6 Entry 5


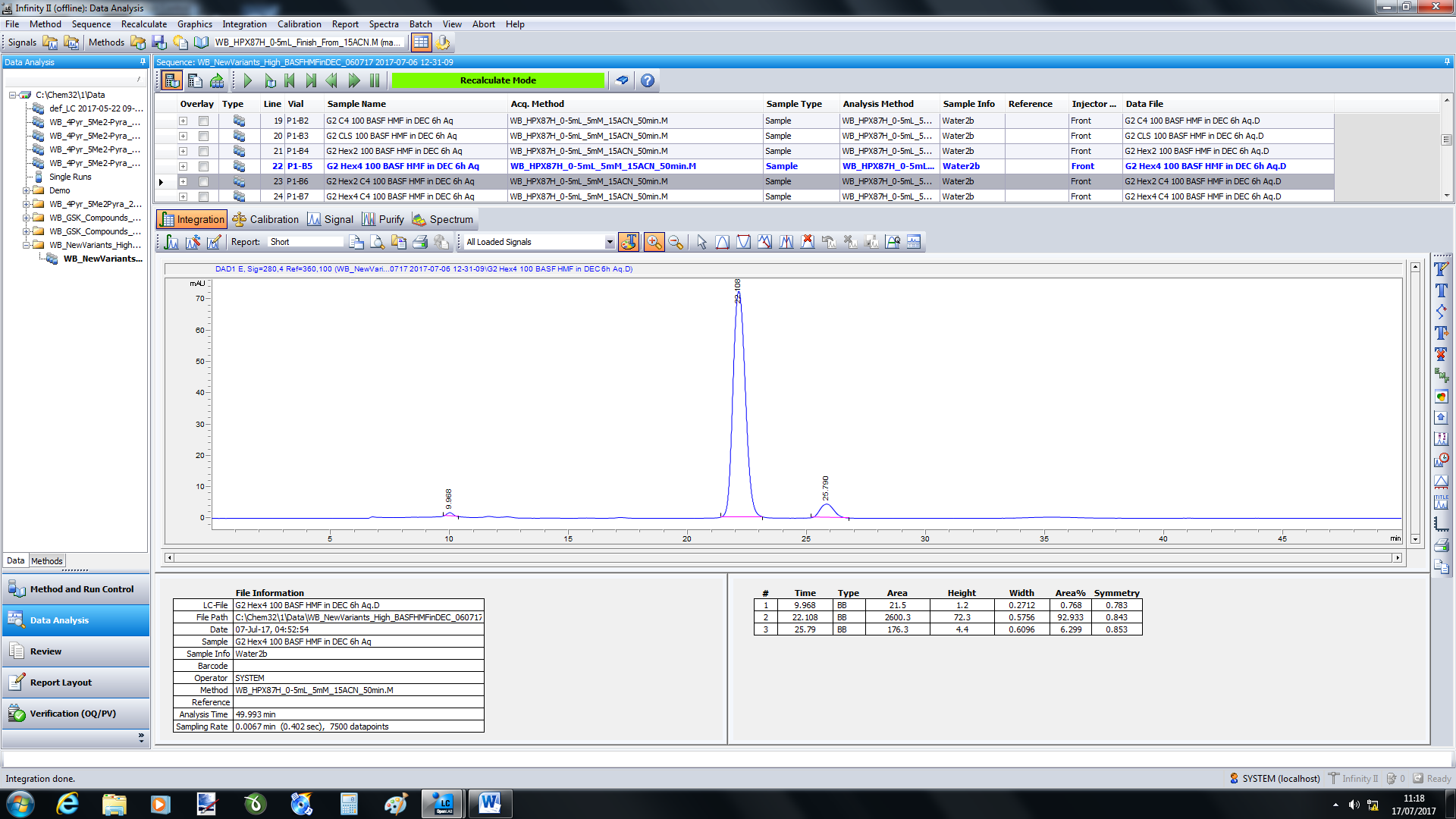


M_5-2_ 100 BASF HMF in DEC 6h (Aqueous phase)

Table 6 Entry 5


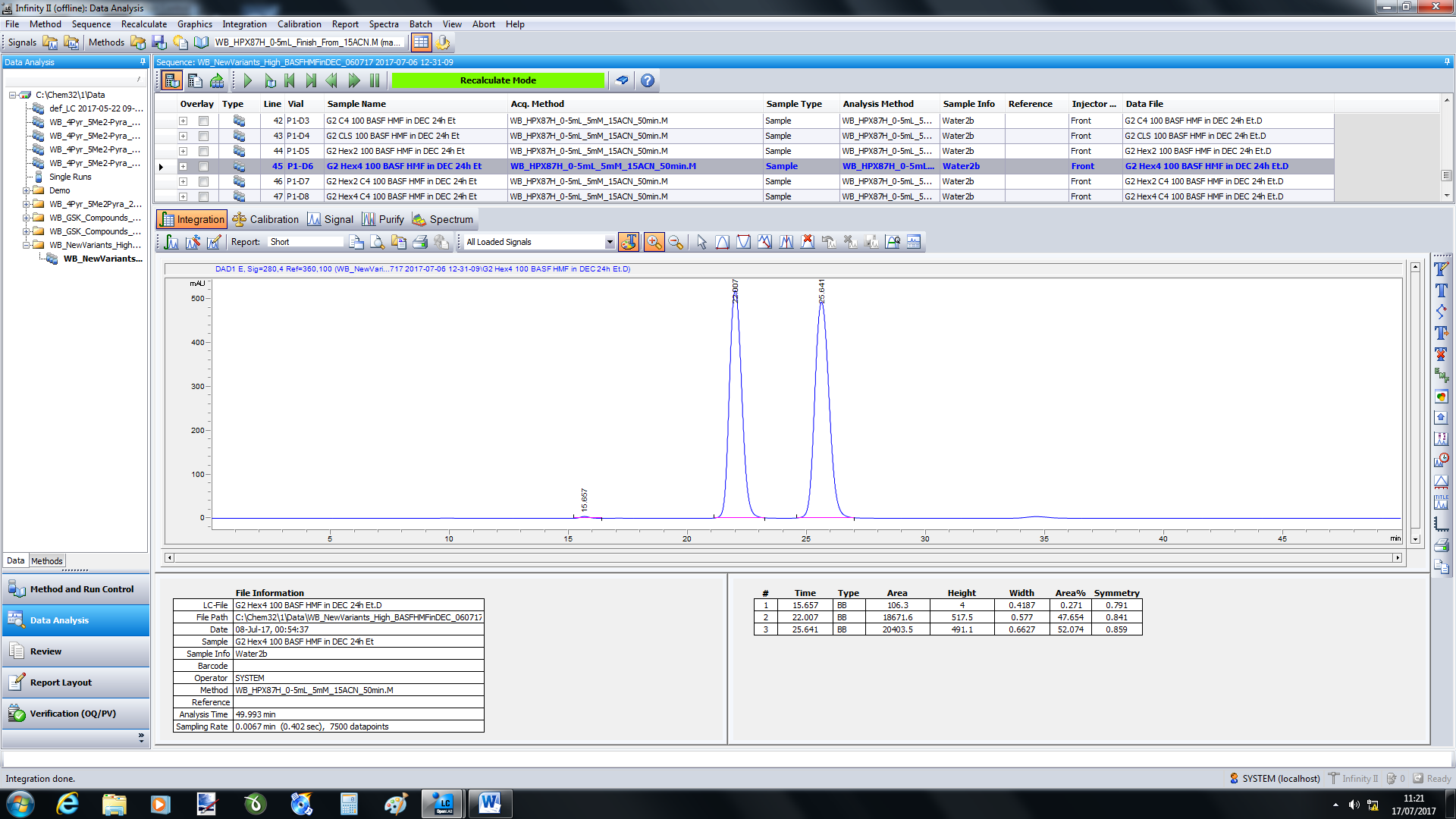


M_5-2_ 100 BASF HMF in DEC 24h (EtOAc phase)

Table 6 Entry 6


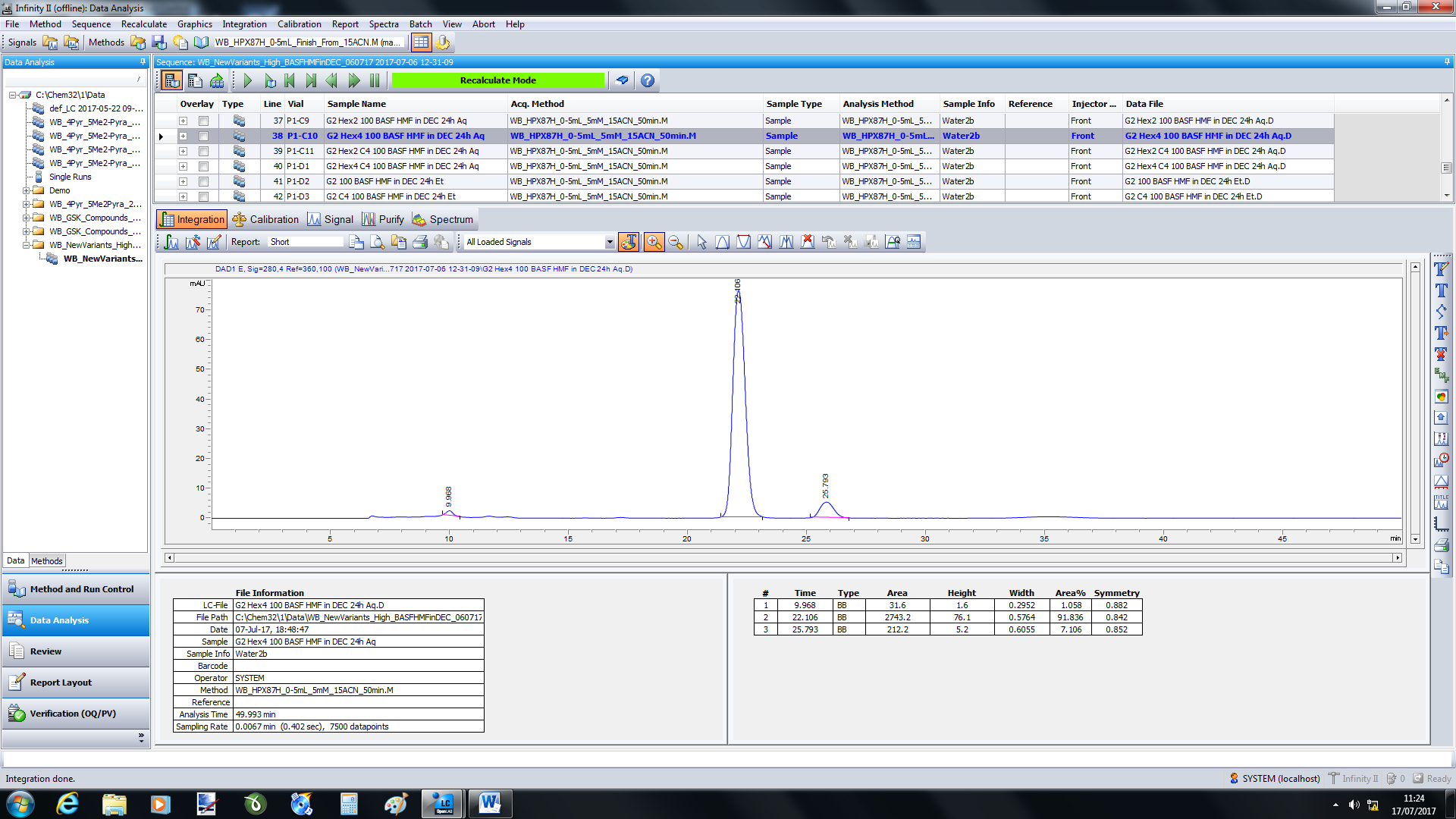


M_5-2_ 100 BASF HMF in DEC 24h (Aqueous phase)

Table 6 Entry 6


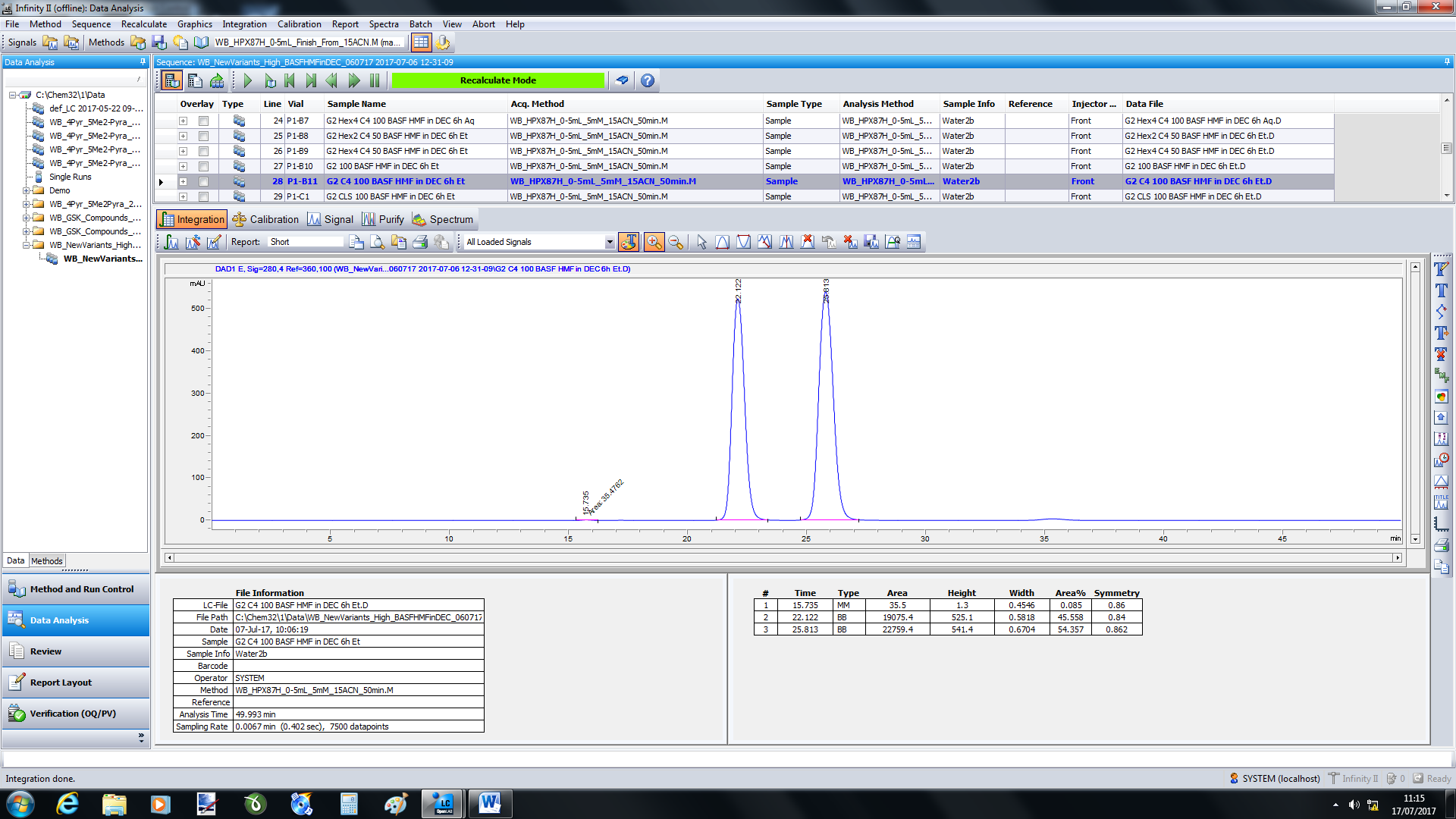


M_6-A_ 100 BASF HMF in DEC 6h (EtOAc phase)

Table 6 Entry 7


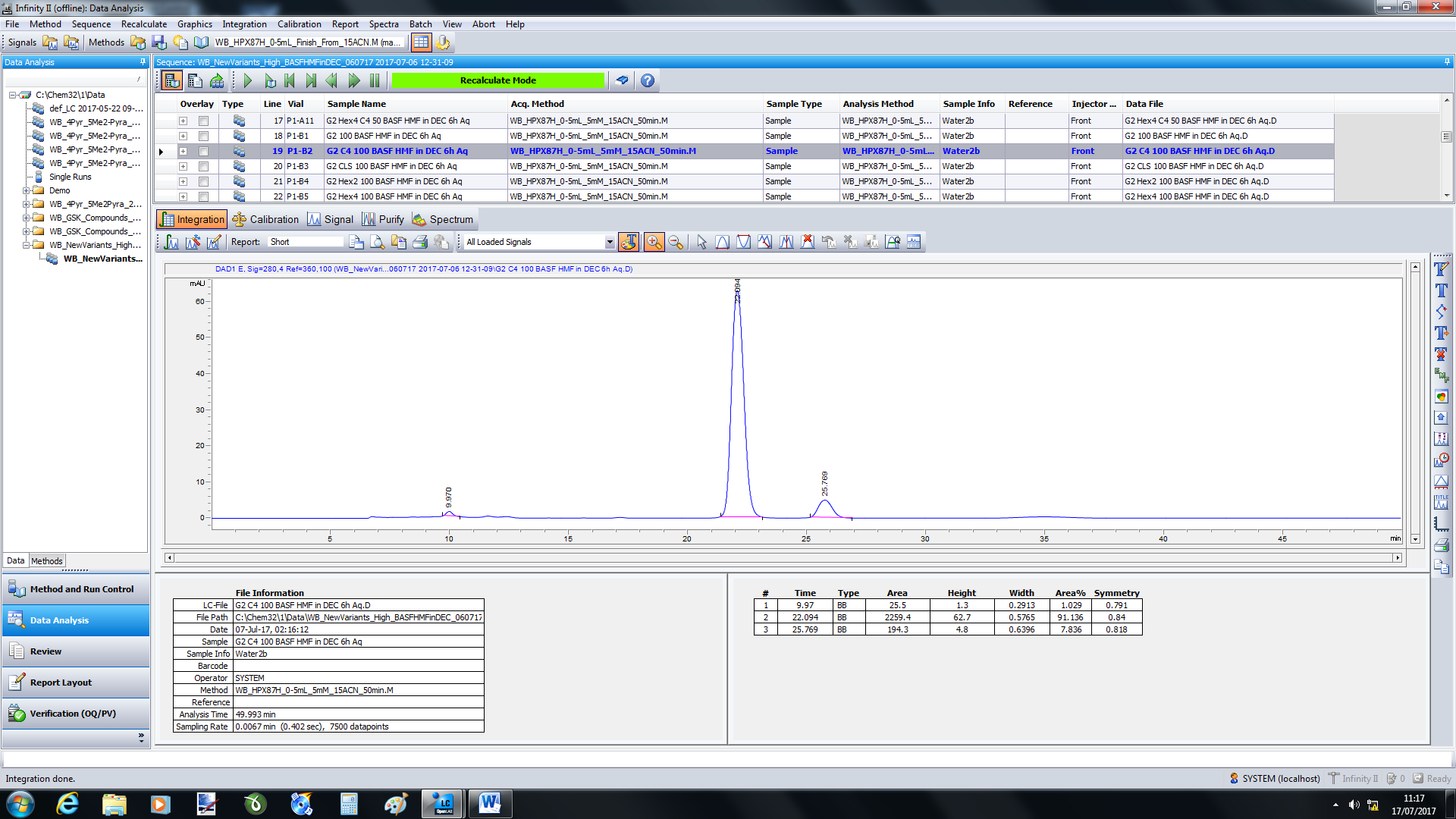


M_6-A_ 100 BASF HMF in DEC 6h (Aqueous phase)

Table 6 Entry 7


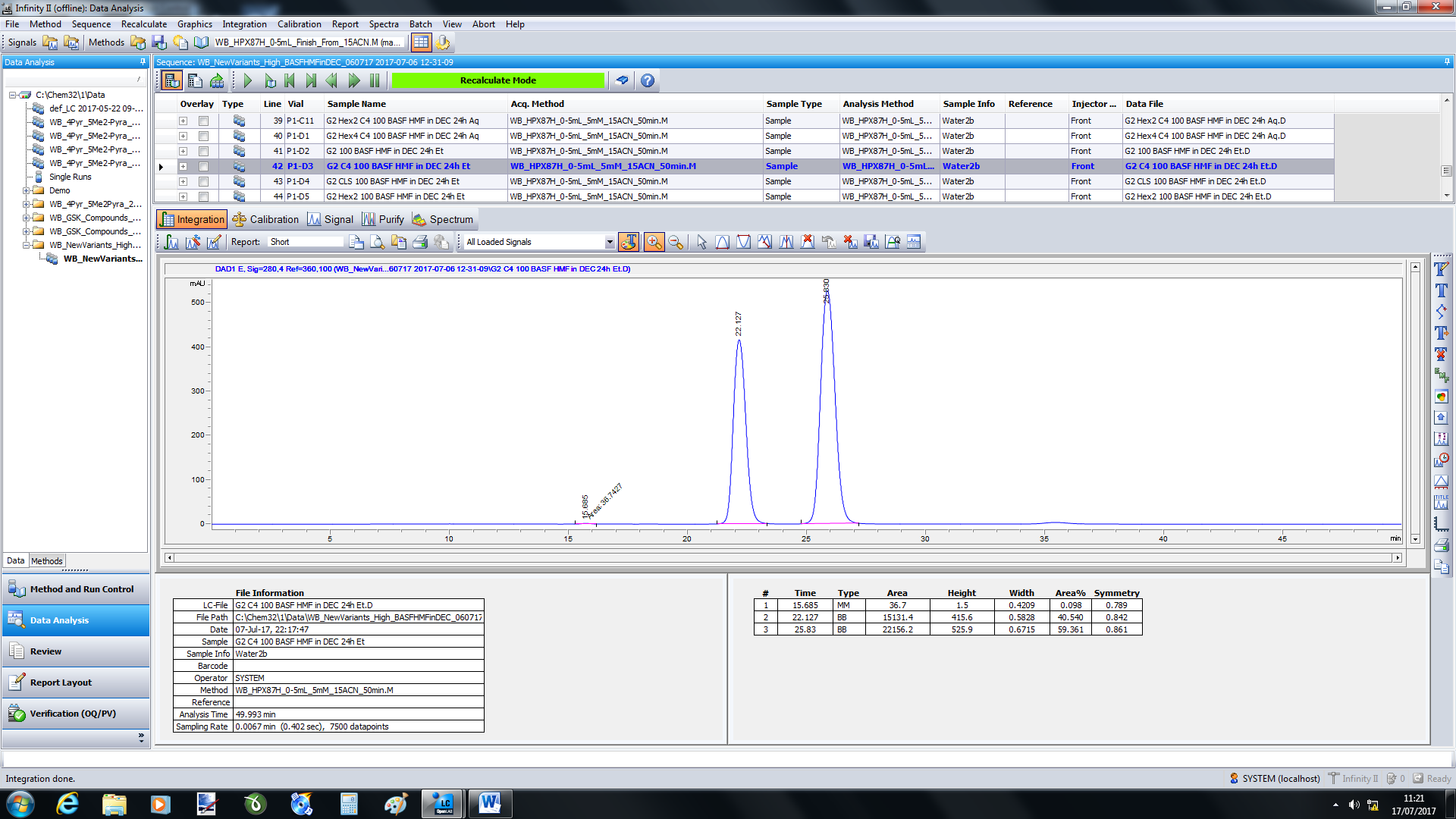


M_6-A_ 100 BASF HMF in DEC 24h (EtOAc phase)

Table 6 Entry 8


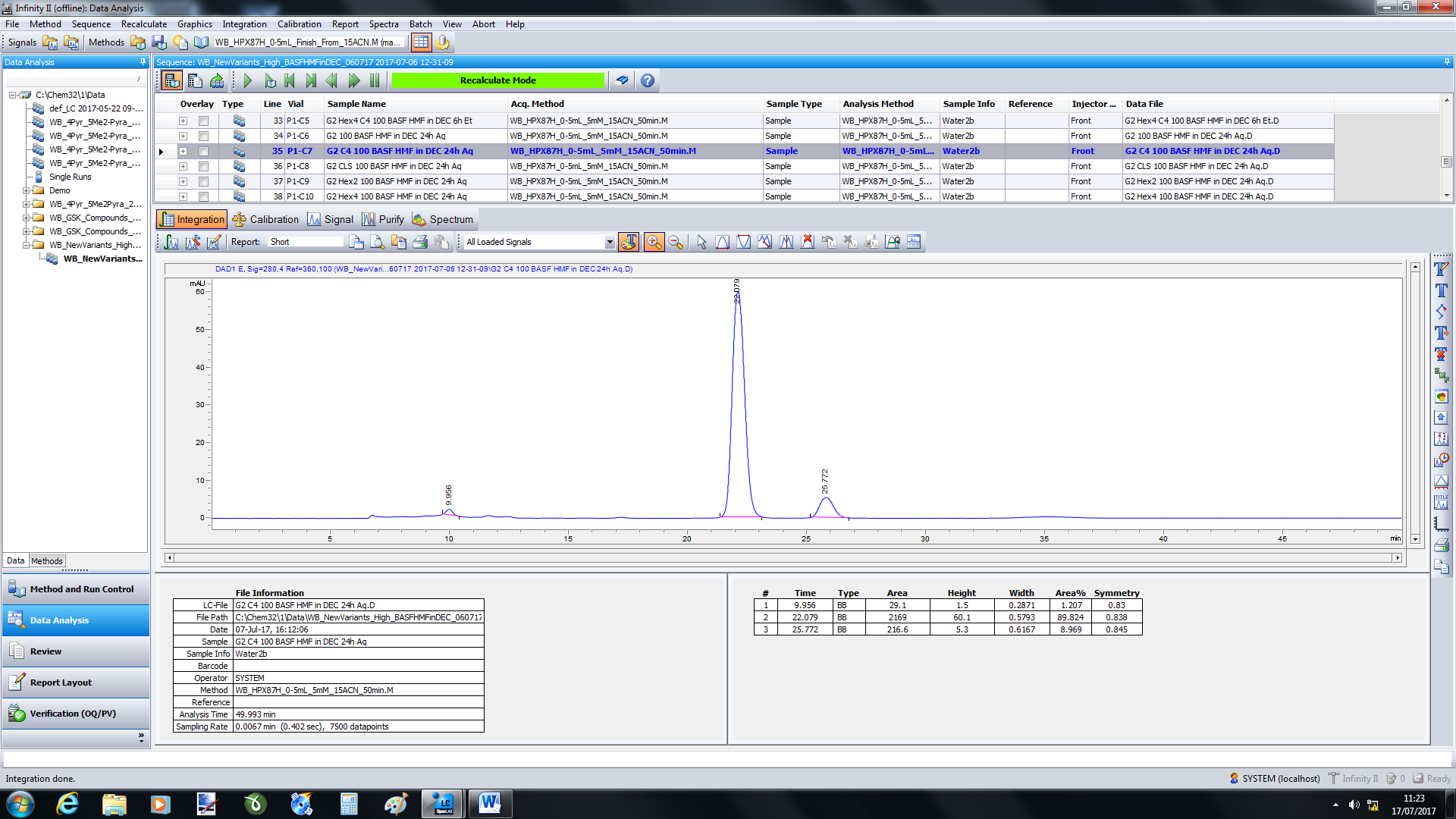


M_6-A_ 100 BASF HMF in DEC 24h (Aqueous phase)

Table 6 Entry 8


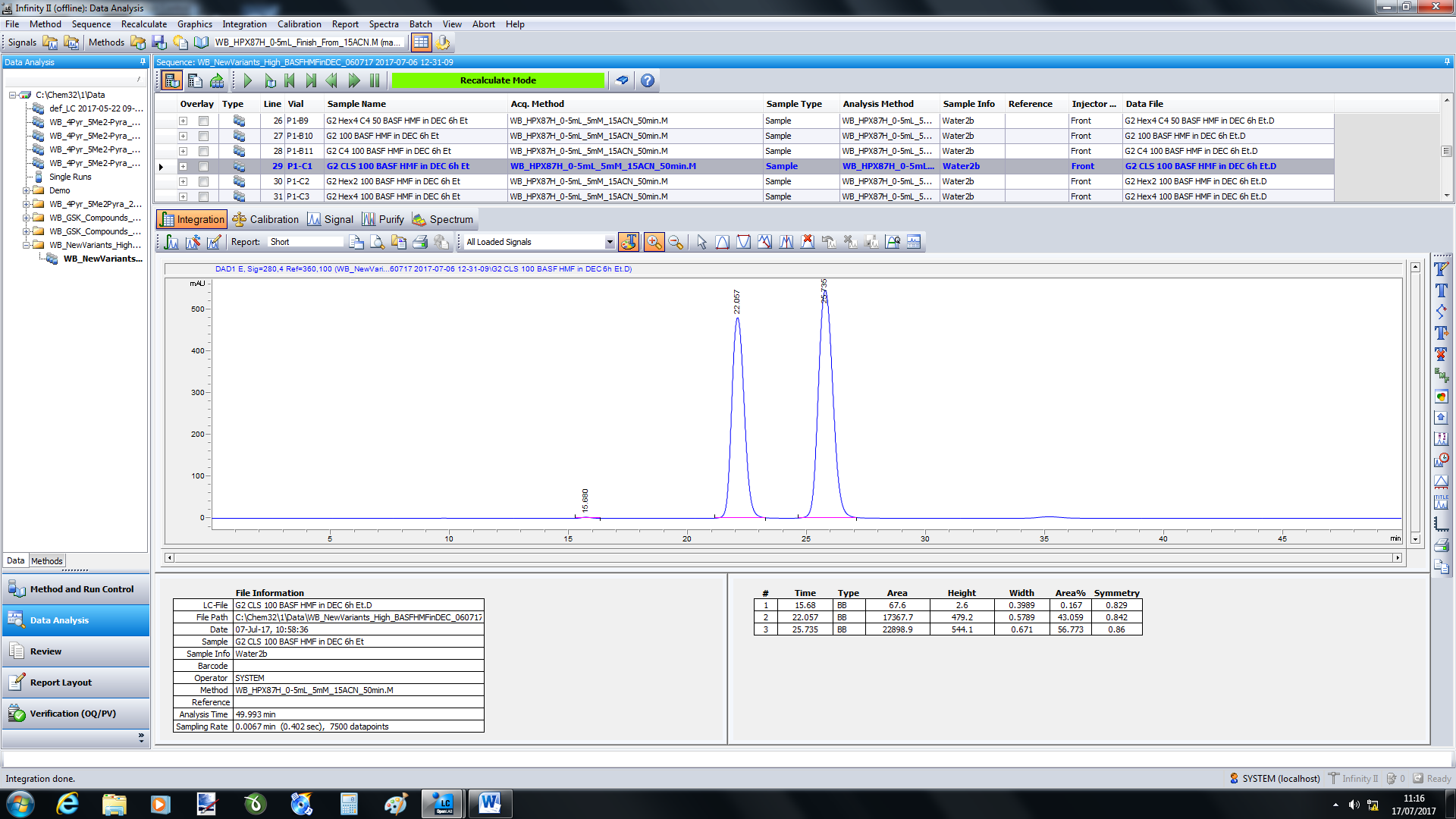


M_6-B_ 100 BASF HMF in DEC 6h (EtOAc phase)

Table 6 Entry 9


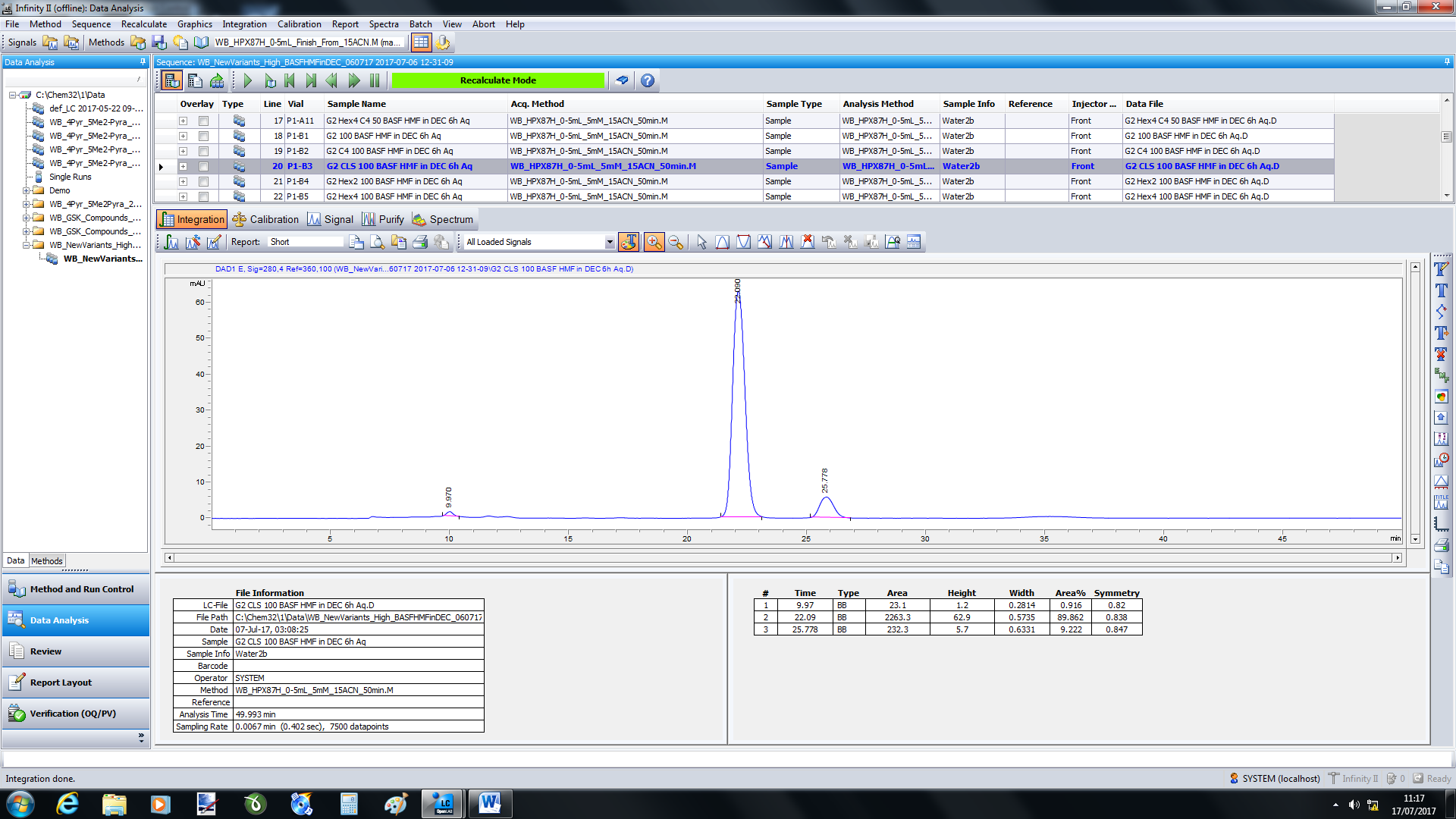


M_6-B_ 100 BASF HMF in DEC 6h (Aqueous phase)

Table 6 Entry 9


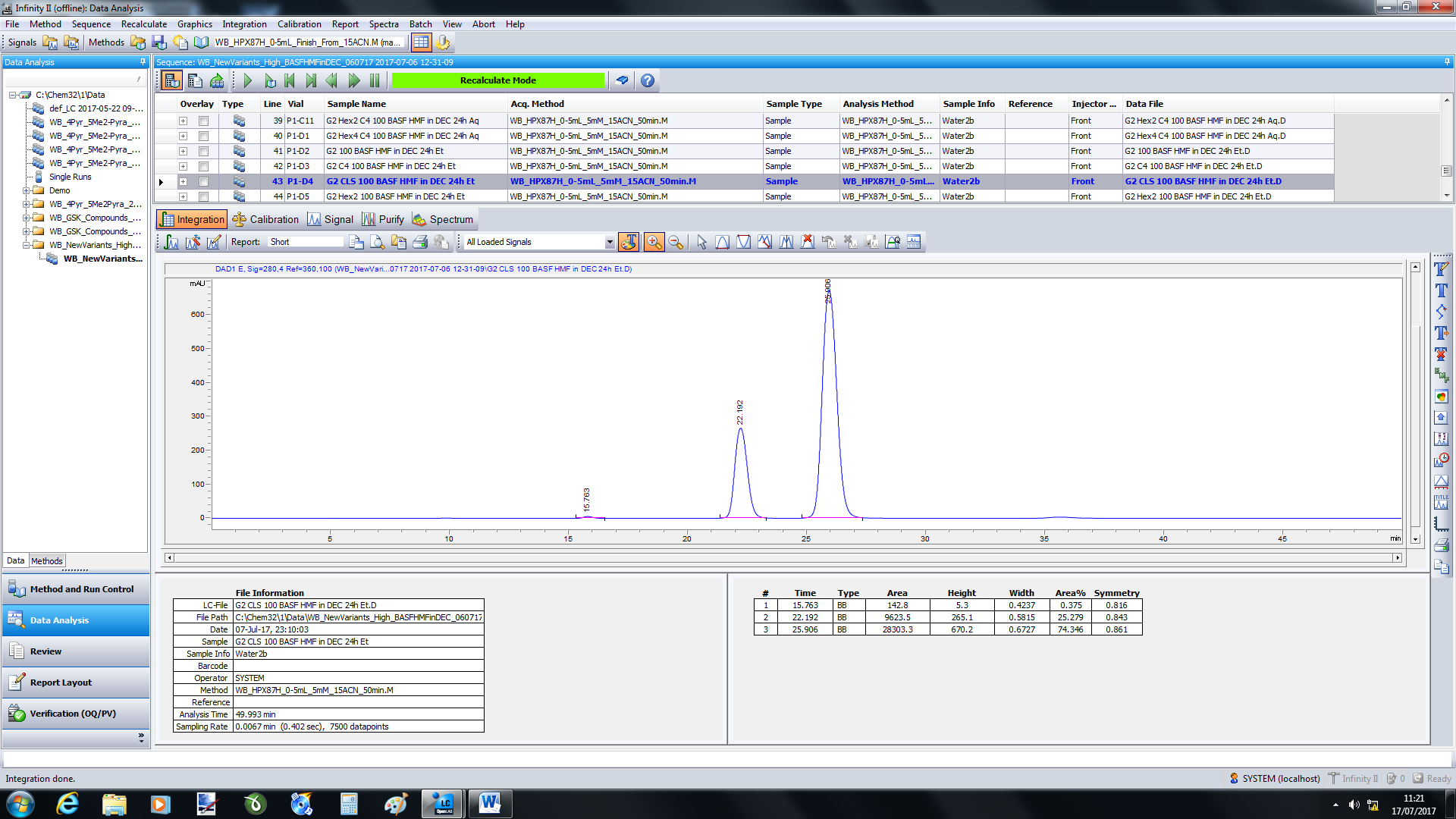


M_6-B_ 100 BASF HMF in DEC 24h (EtOAc phase)

Table 6 Entry 10


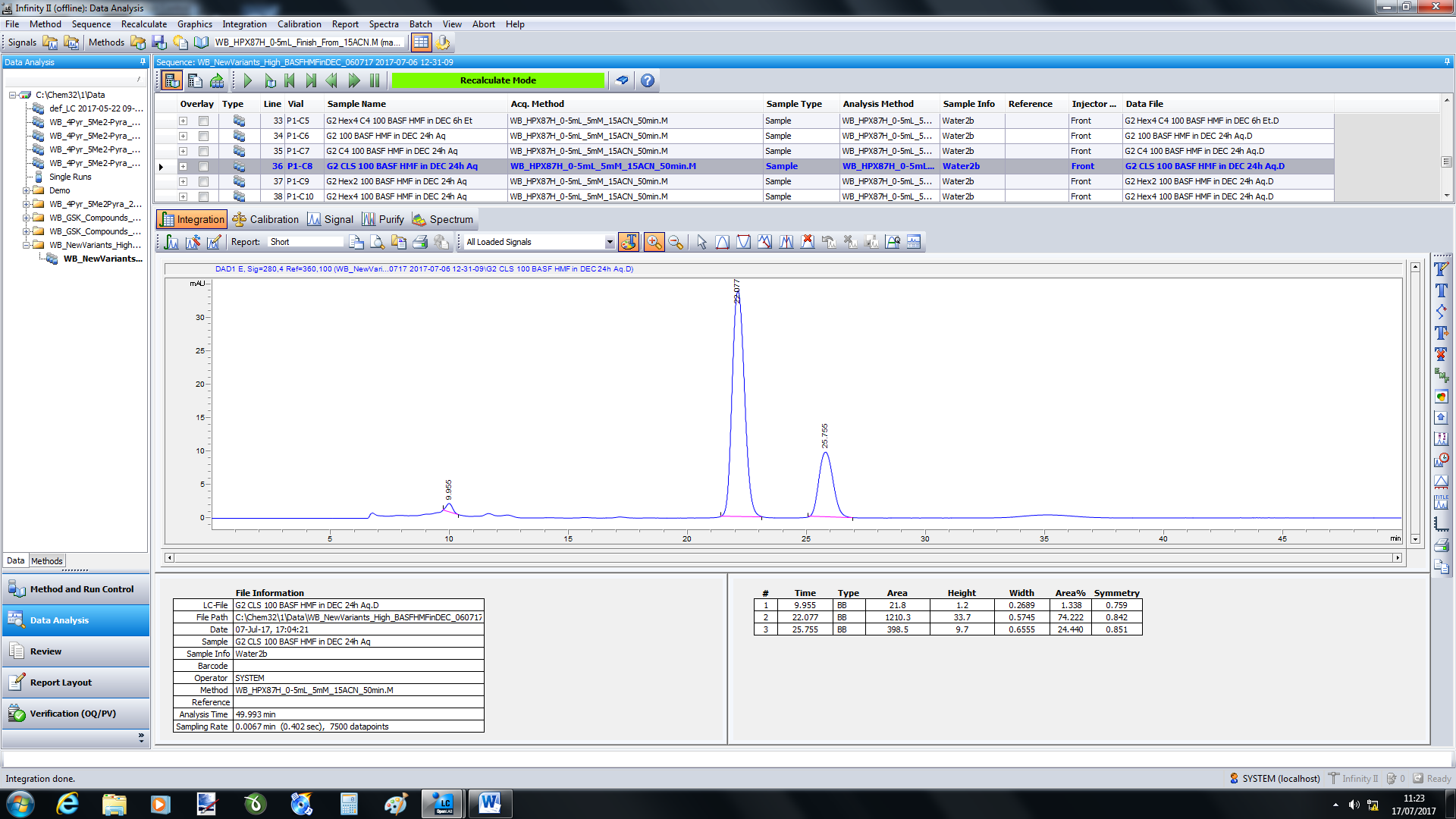


M_6-B_ 100 BASF HMF in DEC 24h (Aqueous phase)

Table 6 Entry 10


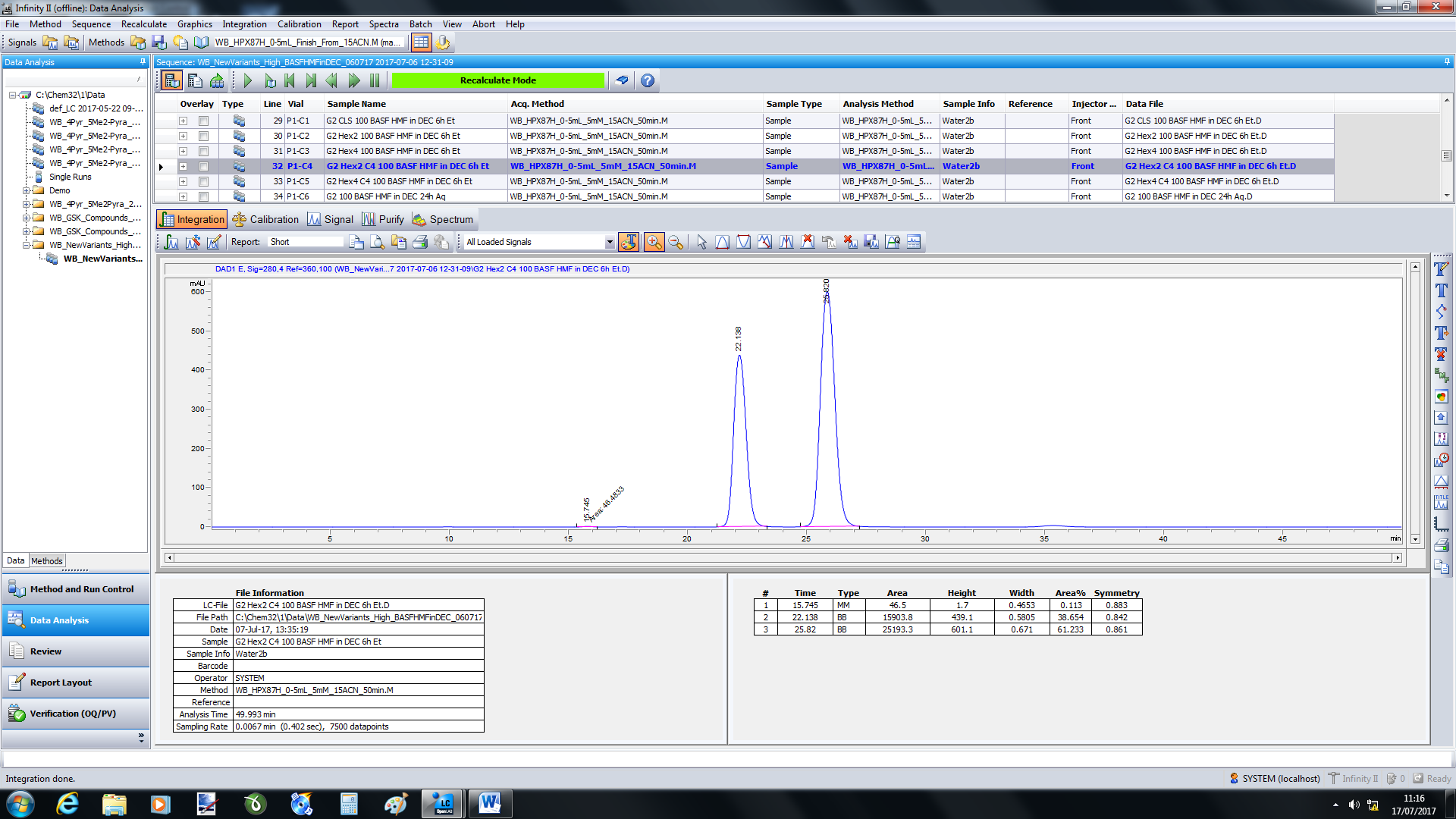


M_7-1A_ 100 BASF HMF in DEC 6h (EtOAc phase)

Table 6 Entry 11


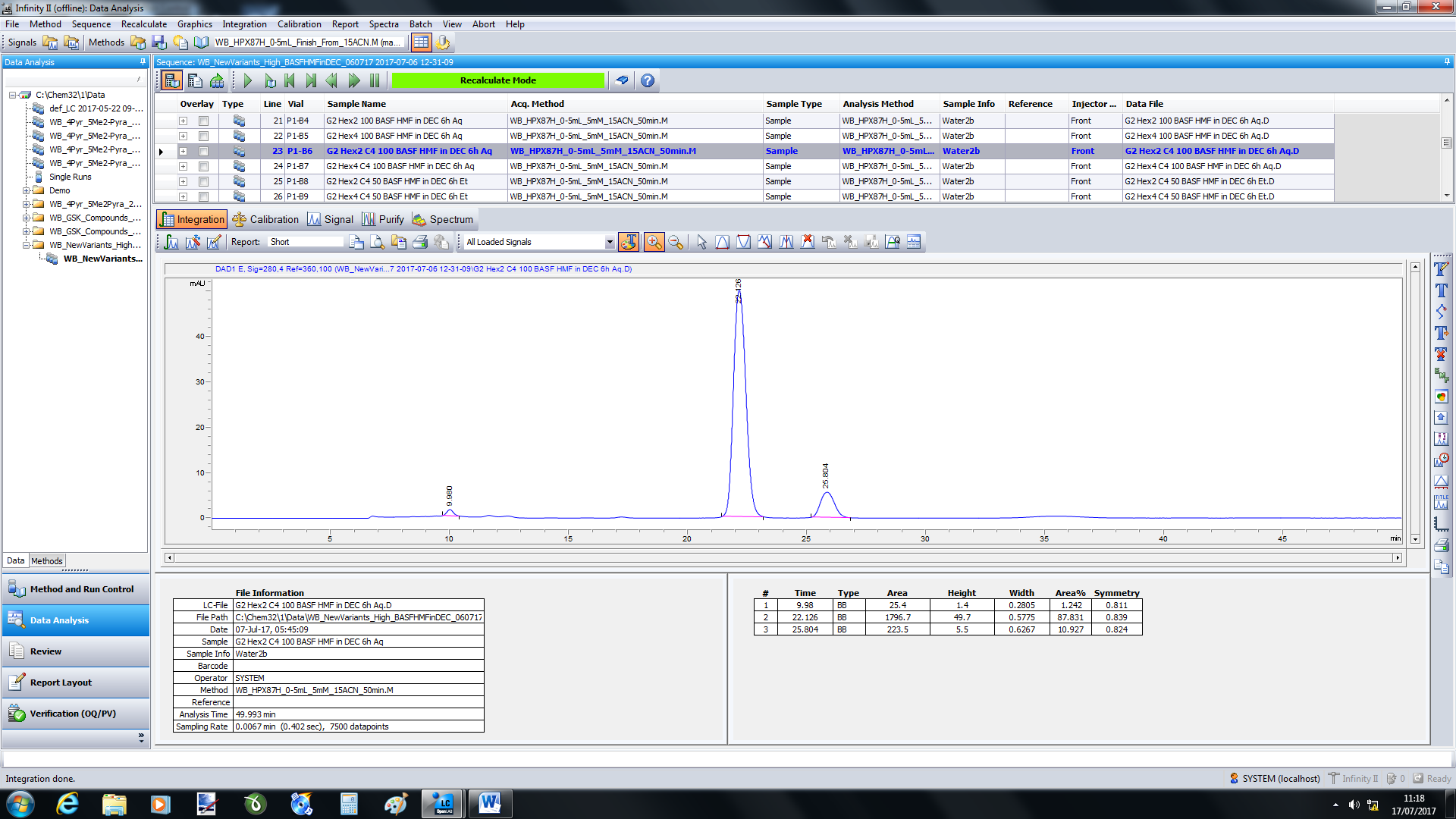


M_7-1A_ 100 BASF HMF in DEC 6h (Aqueous phase)

Table 6 Entry 11


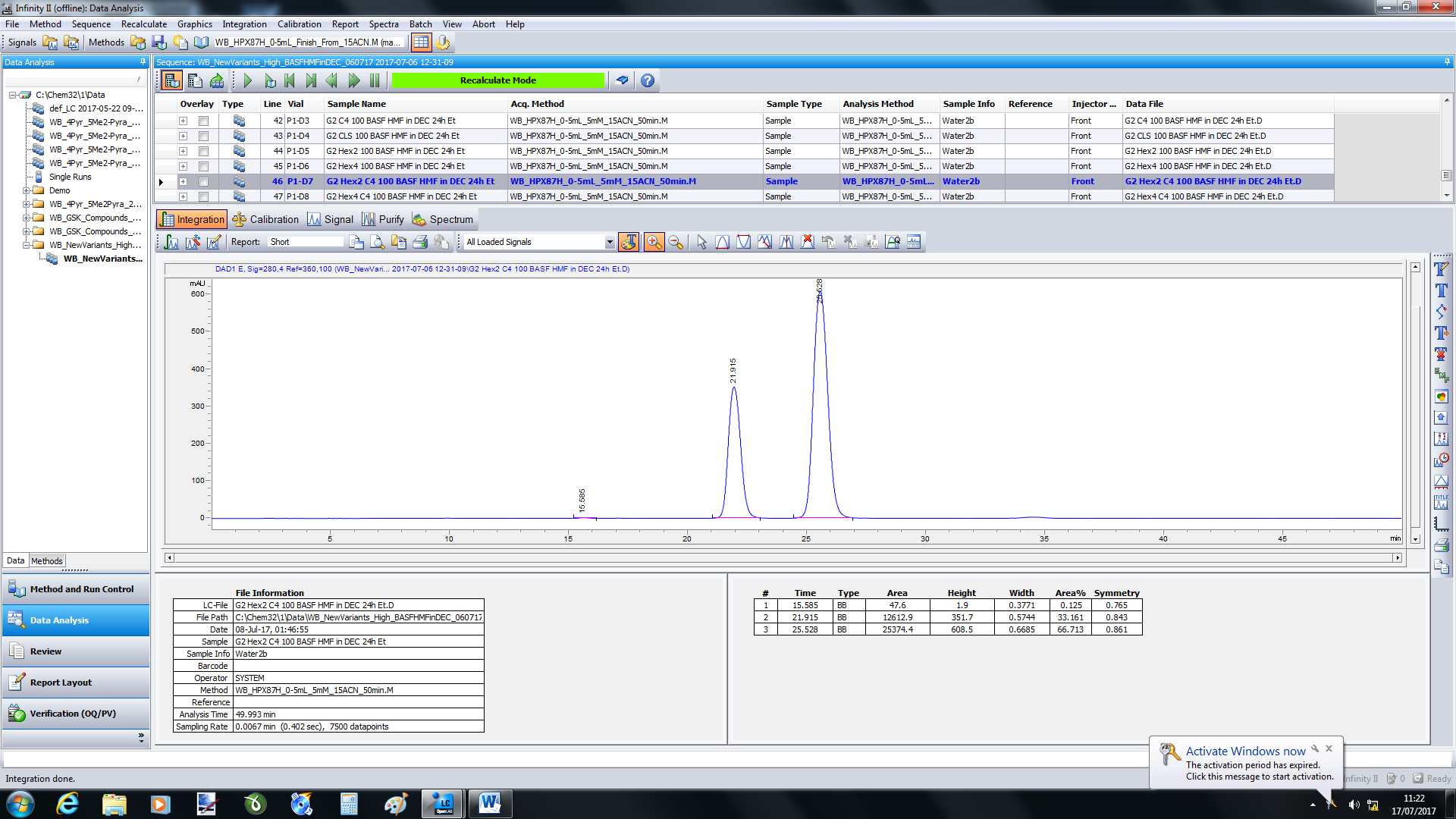


M_7-1A_ 100 BASF HMF in DEC 24h (EtOAc phase)

Table 6 Entry 12


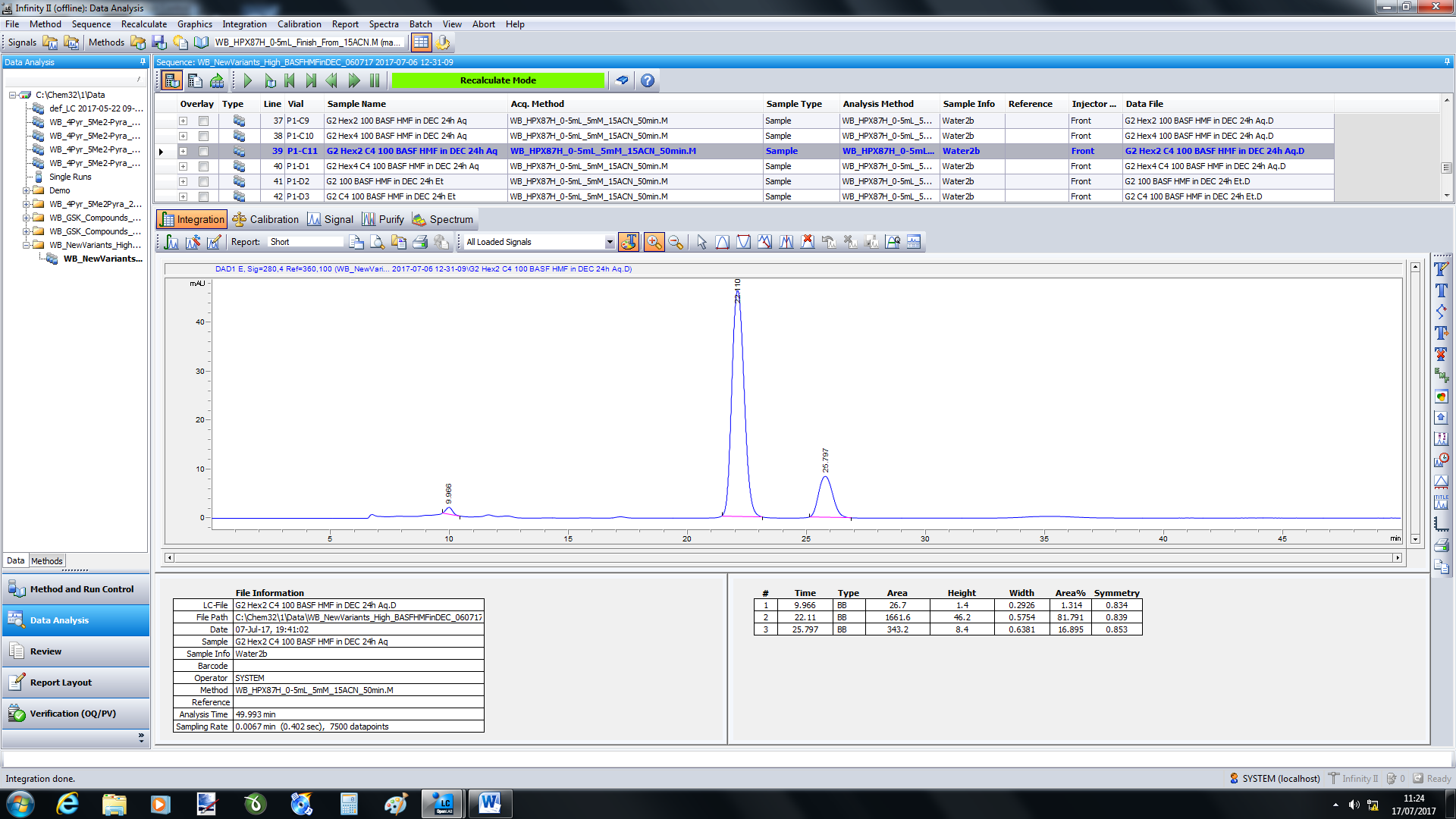


M_7-1A_ 100 BASF HMF in DEC 24h (Aqueous phase)

Table 6 Entry 12


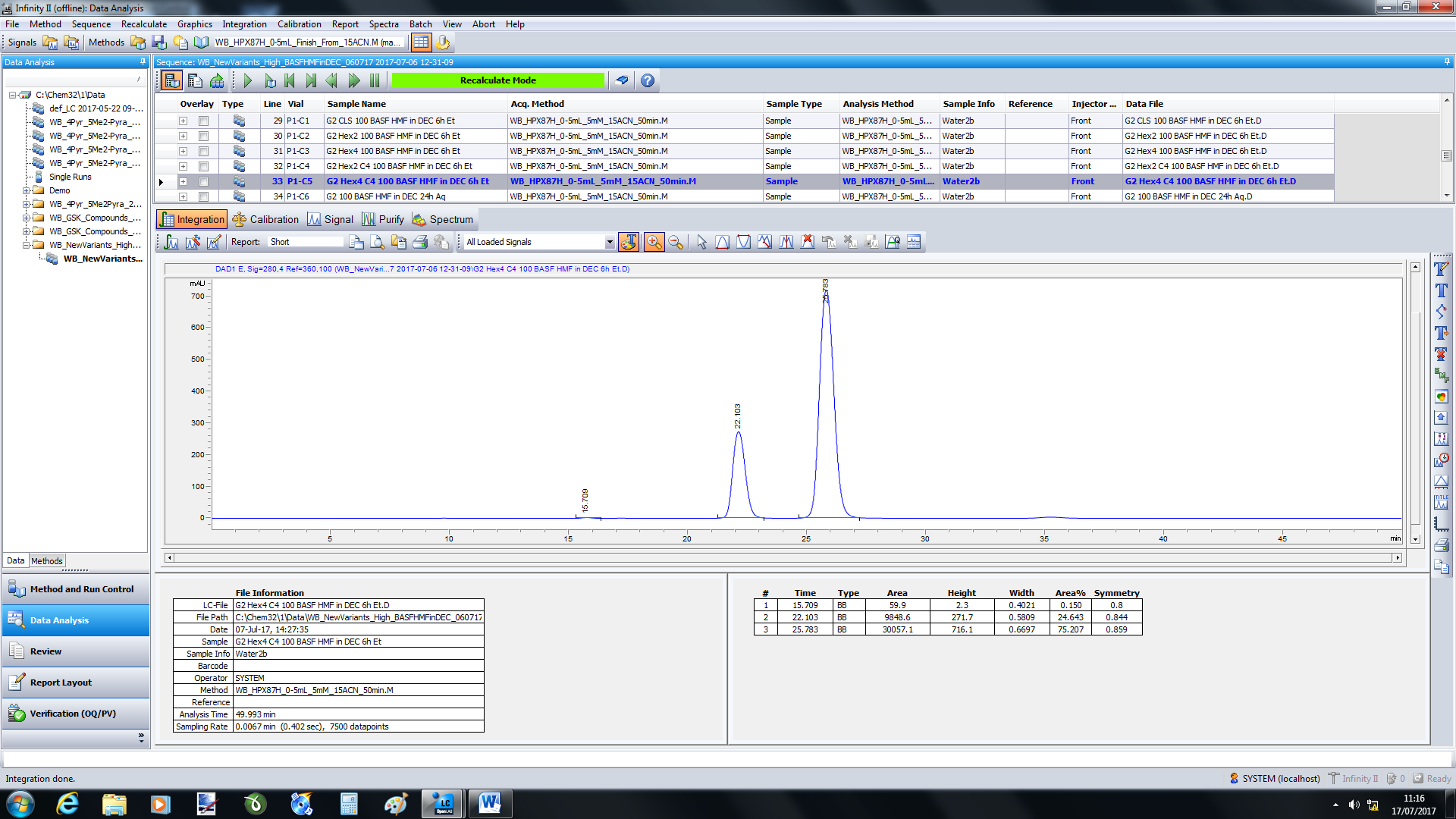


M_7-2A_ 100 BASF HMF in DEC 6h (EtOAc phase)

Table 6 Entry 13


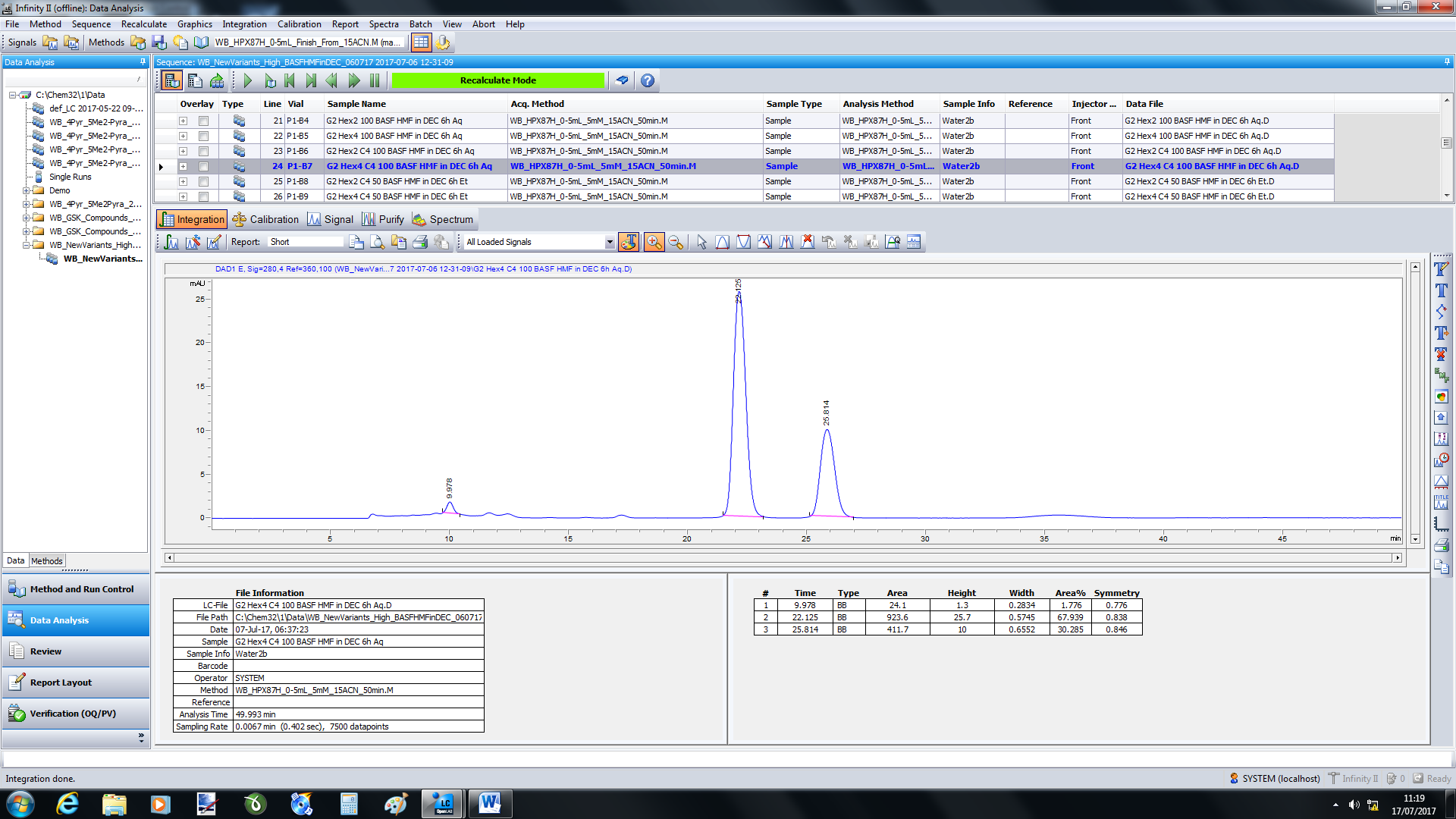


M_7-2A_ 100 BASF HMF in DEC 6h (Aqueous phase)

Table 6 Entry 13


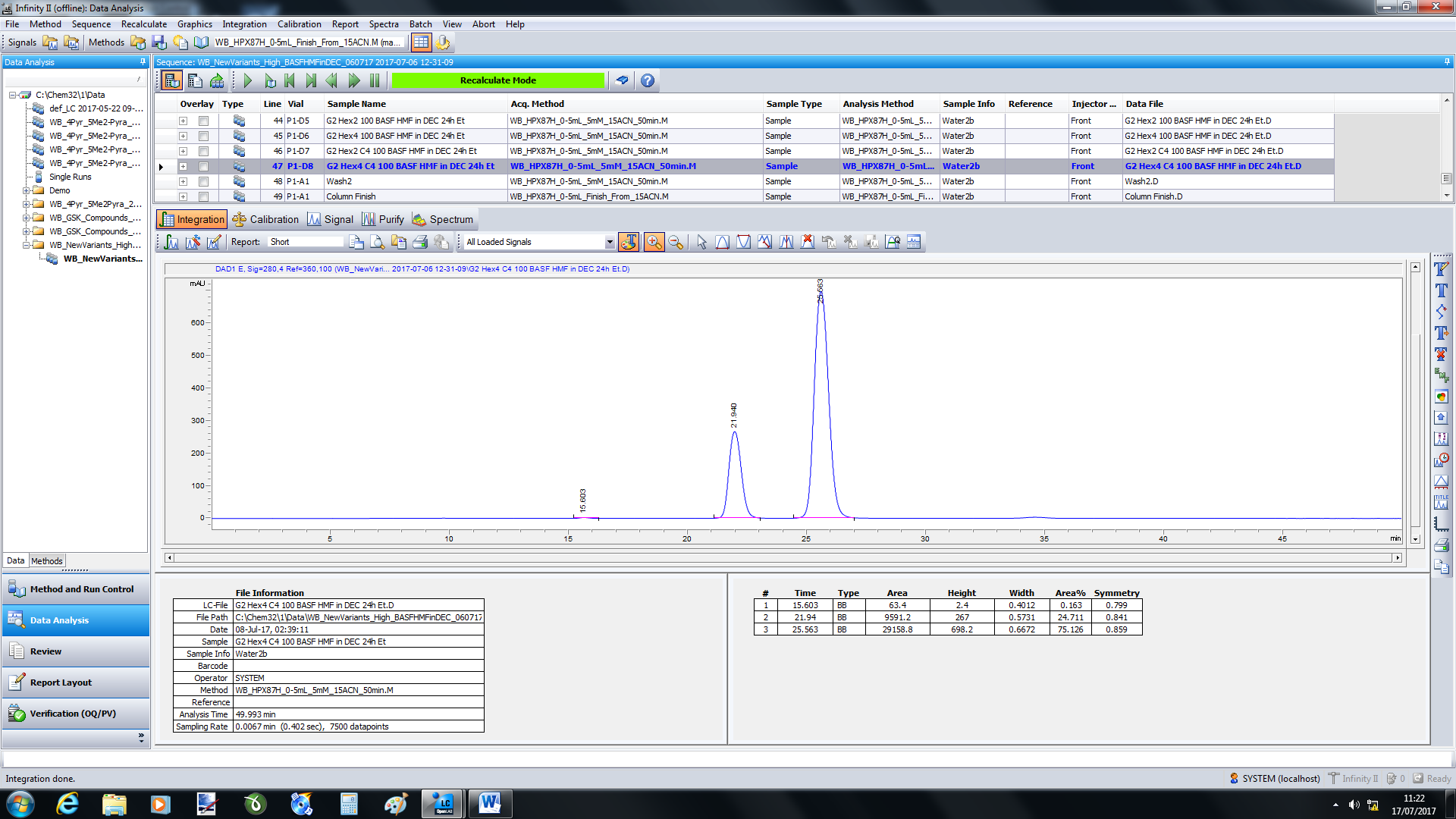


M_7-2A_ 100 BASF HMF in DEC 24h (EtOAc phase)

Table 6 Entry 14


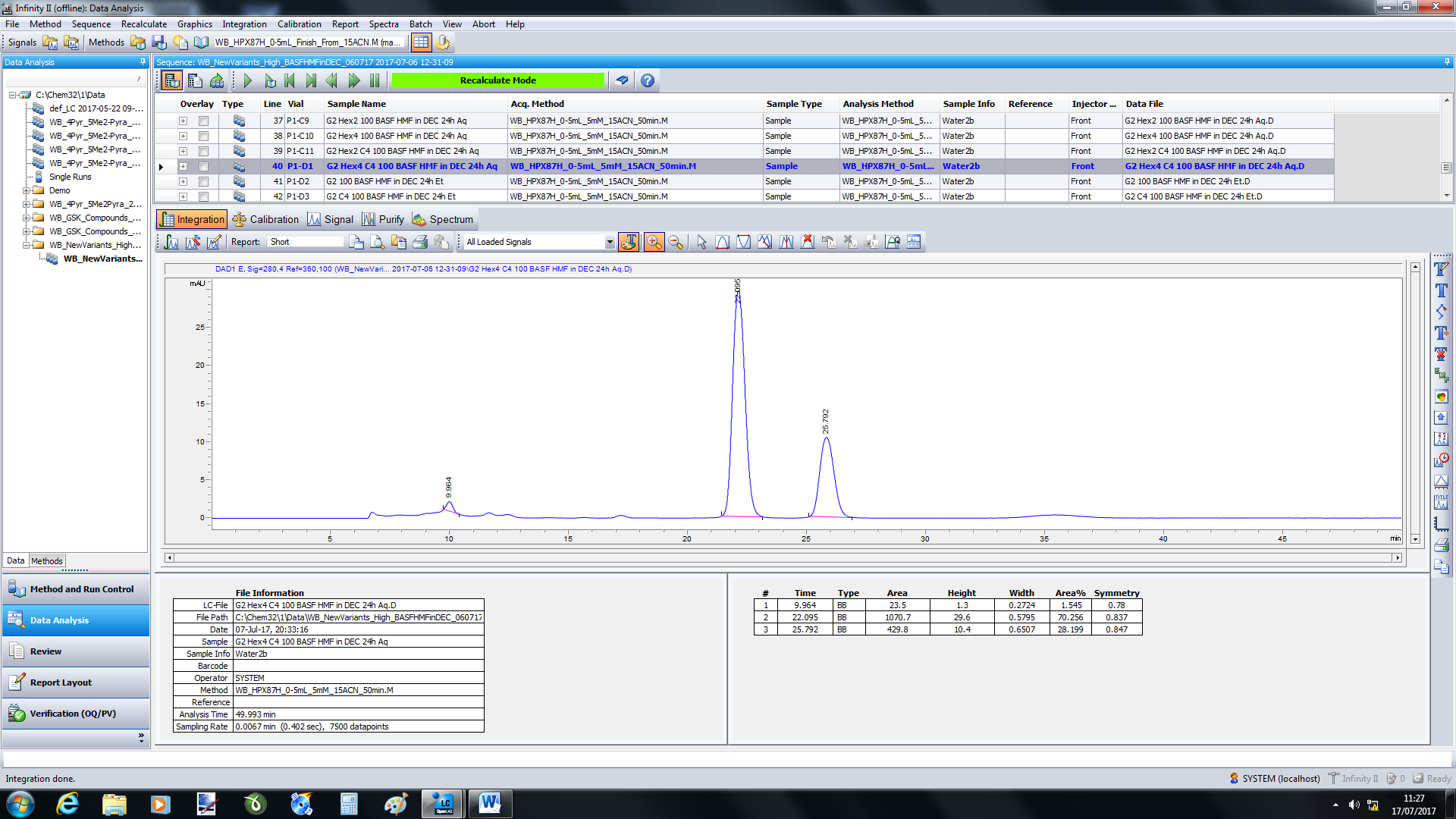


M_7-2A_ 100 BASF HMF in DEC 24h (Aqueous phase)

Table 6 Entry 14


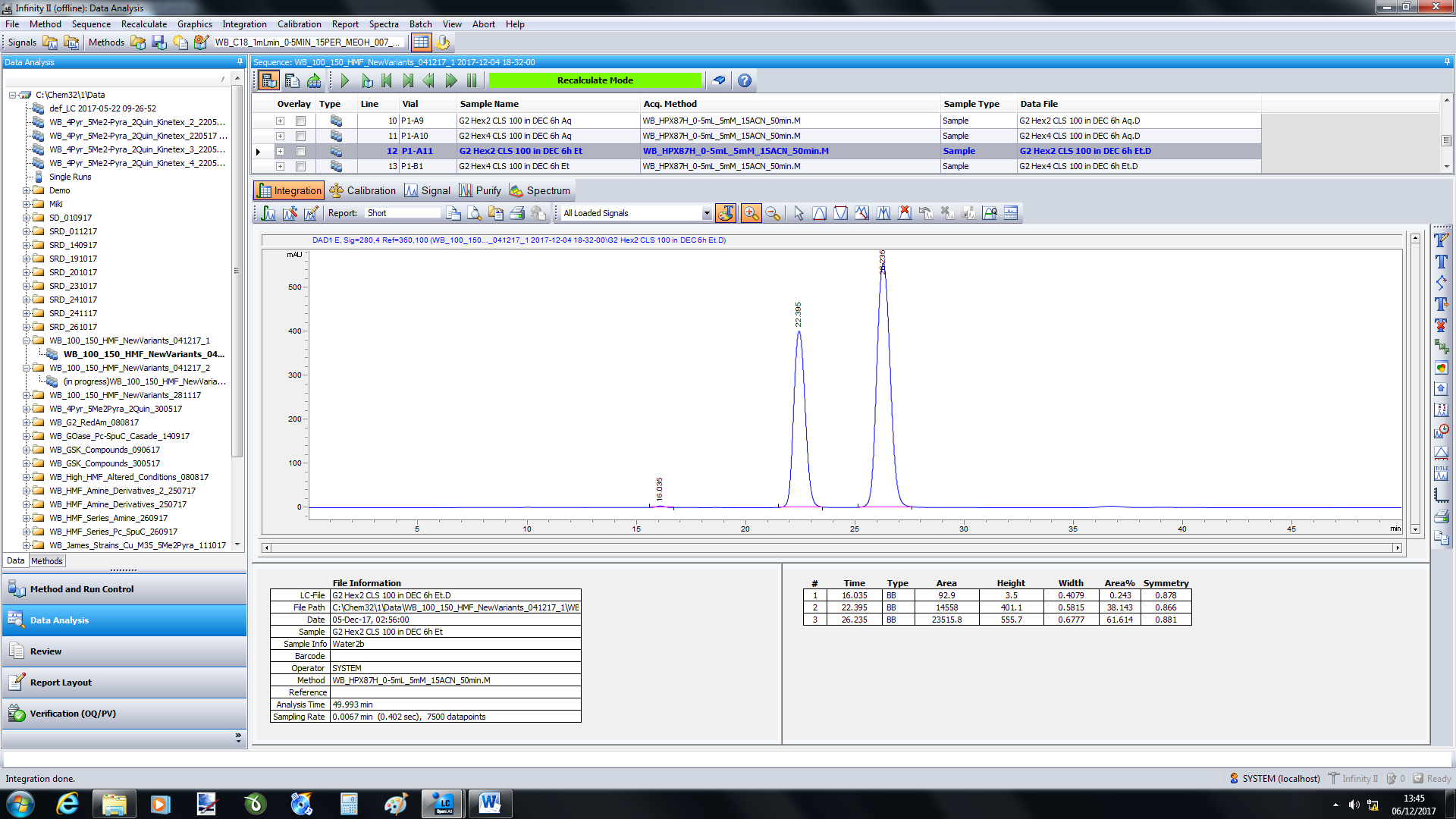


M_7-1B_ 100 BASF HMF in DEC 6h (EtOAc phase)

Table 6 Entry 15


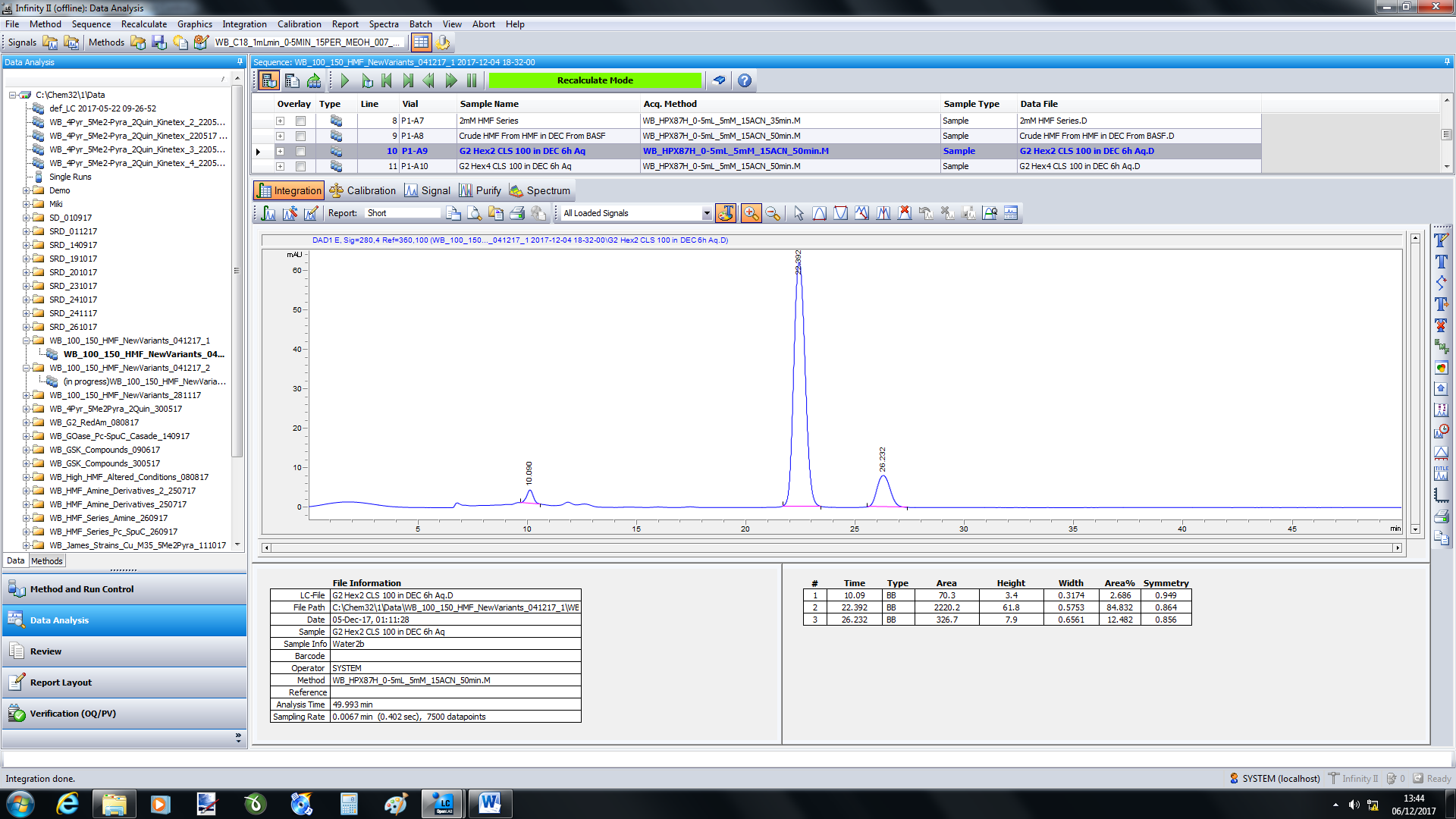


M_7-1B_ 100 BASF HMF in DEC 6h (Aqueous phase)

Table 6 Entry 15


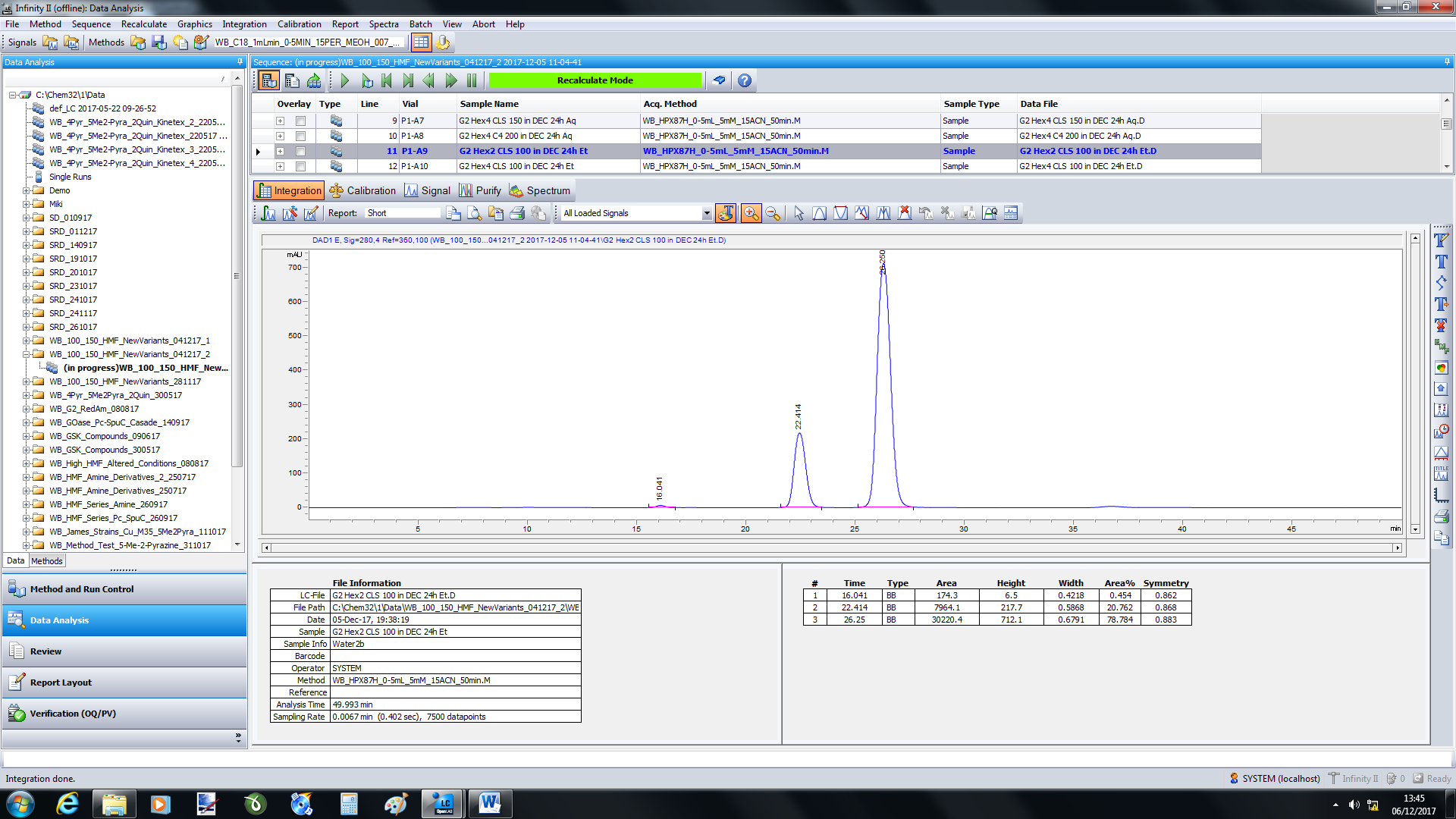


M_7-1B_ 100 BASF HMF in DEC 24h (EtOAc phase)

Table 6 Entry 16


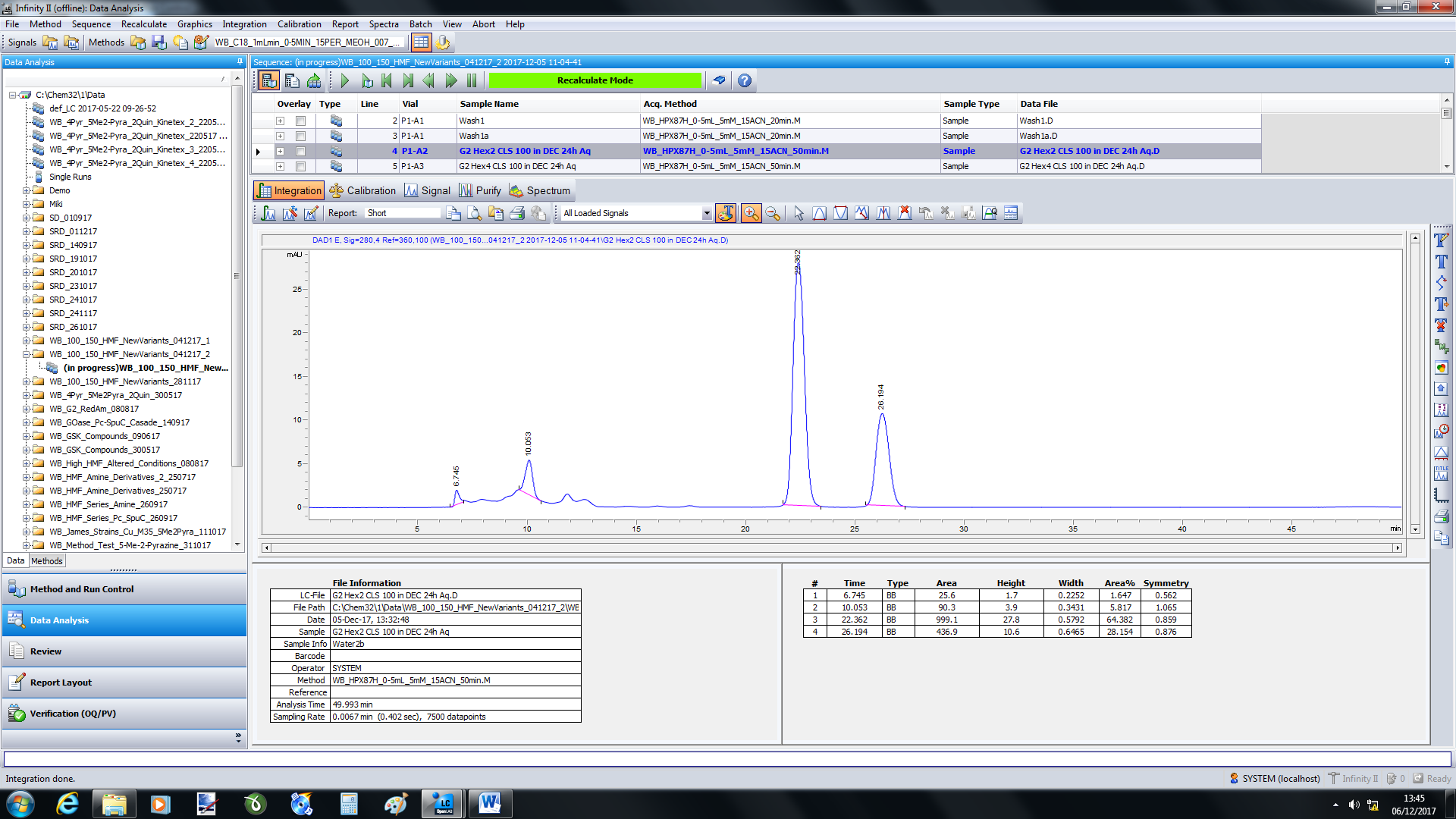


M_7-1B_ 100 BASF HMF in DEC 24h (Aqueous phase)

Table 6 Entry 16


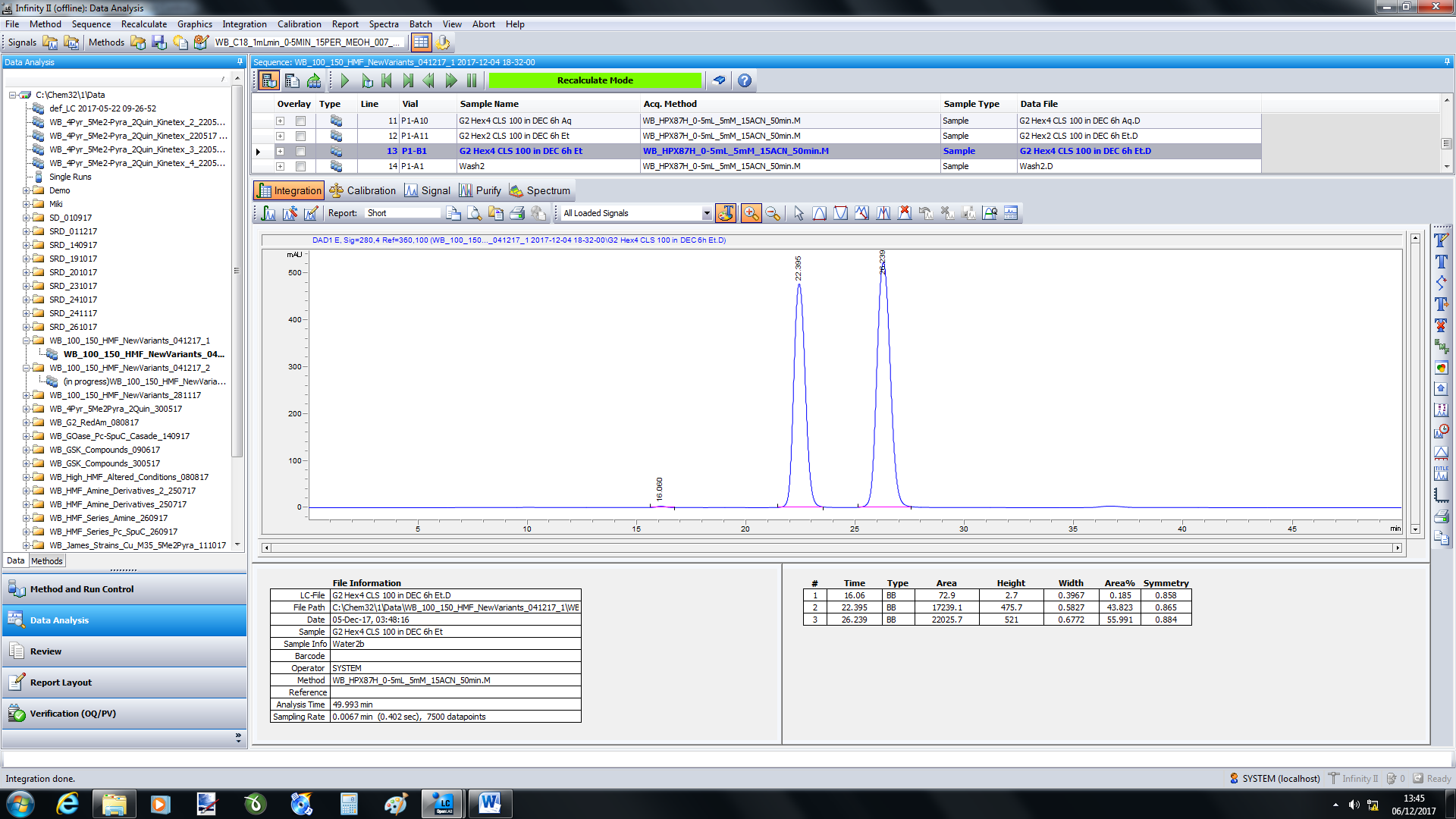


M_7-2B_ 100 BASF HMF in DEC 6h (EtOAc phase)

Table 6 Entry 17


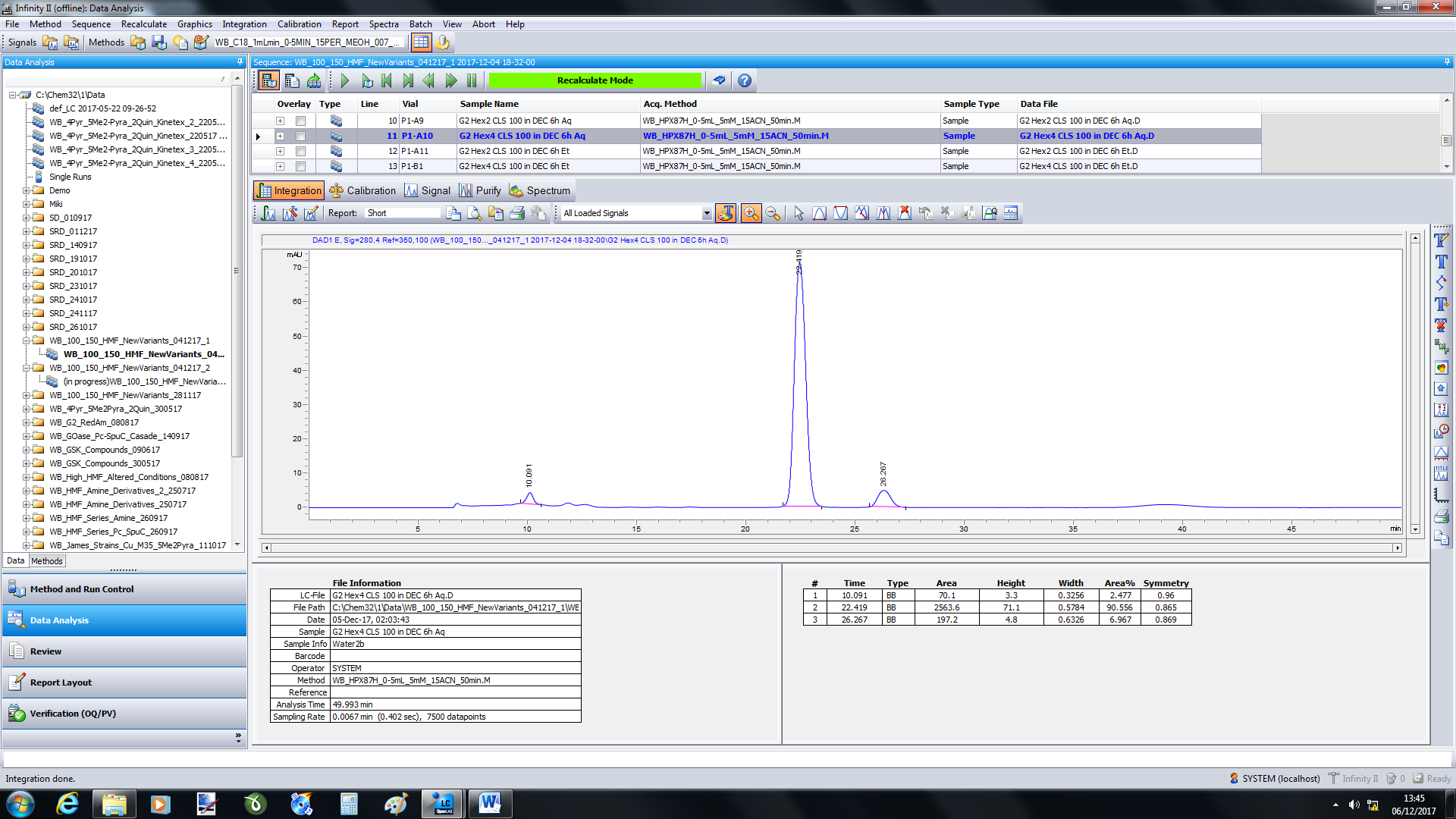


M_7-2B_ 100 BASF HMF in DEC 6h (Aqueous phase)

Table 6 Entry 17


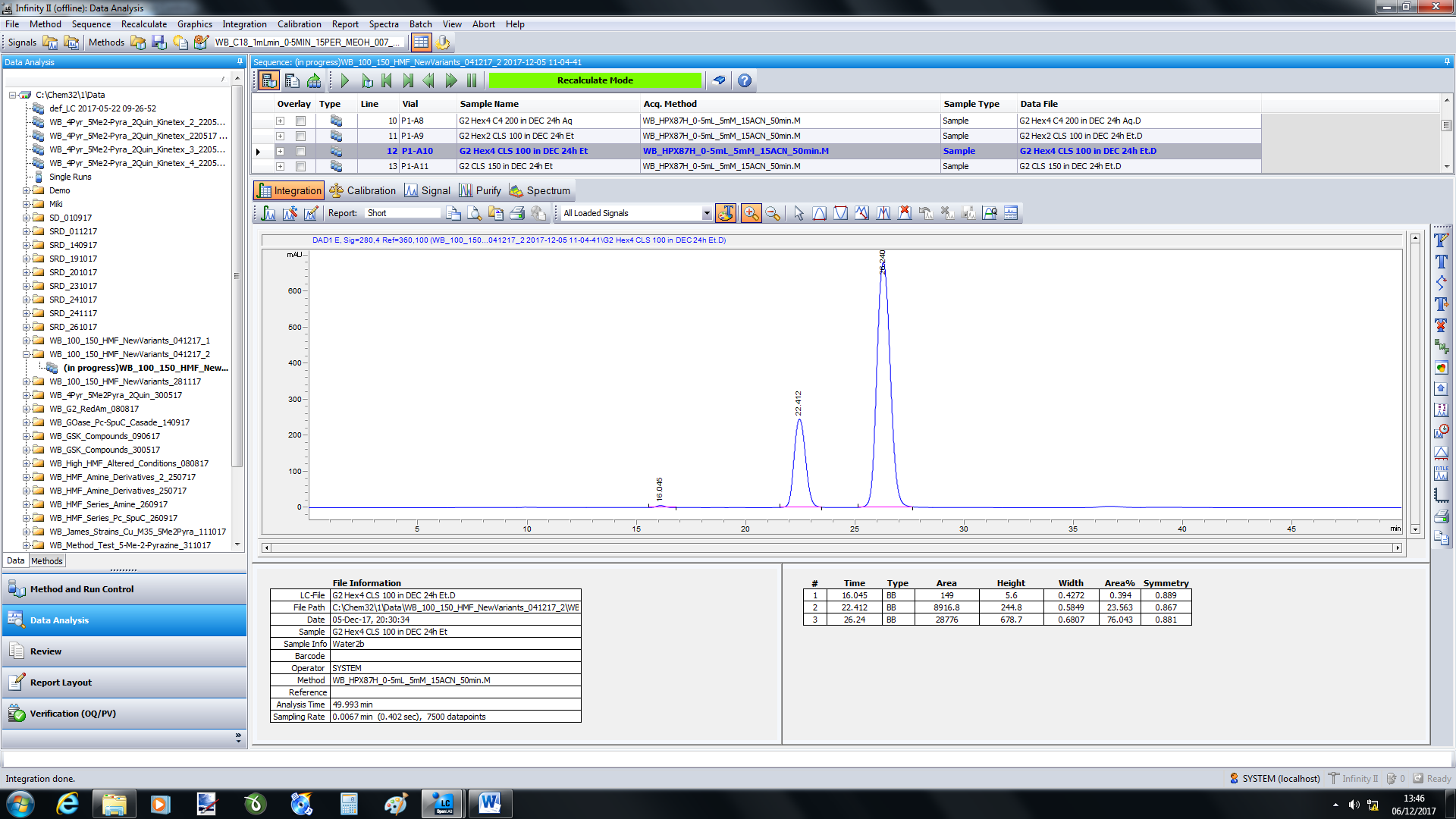


M_7-2B_ 100 BASF HMF in DEC 24h (EtOAc phase)

Table 6 Entry 18


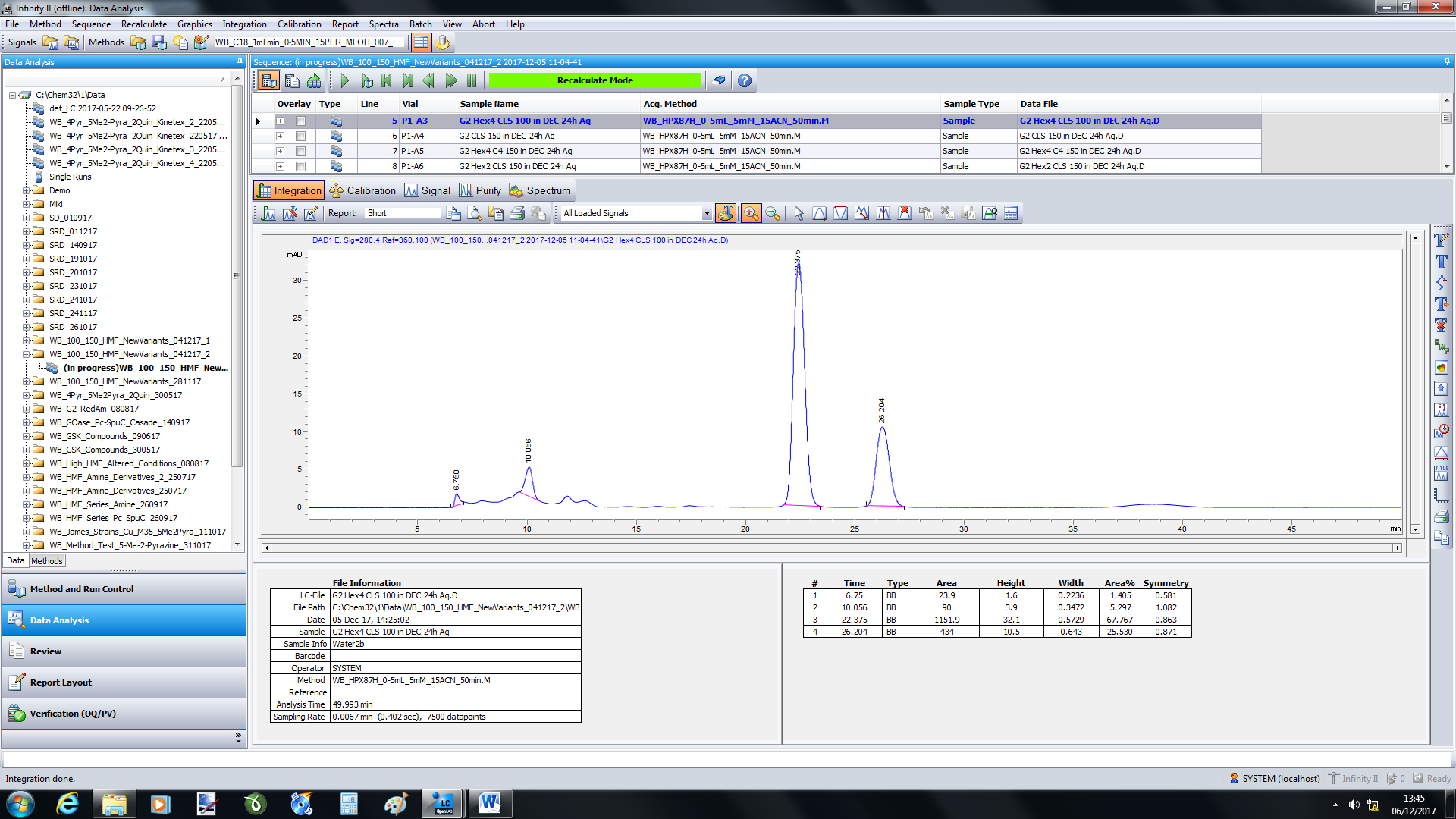


M_7-2B_ 100 BASF HMF in DEC 24h (Aqueous phase)

Table 6 Entry 18

The following data is presented in Figure 3:


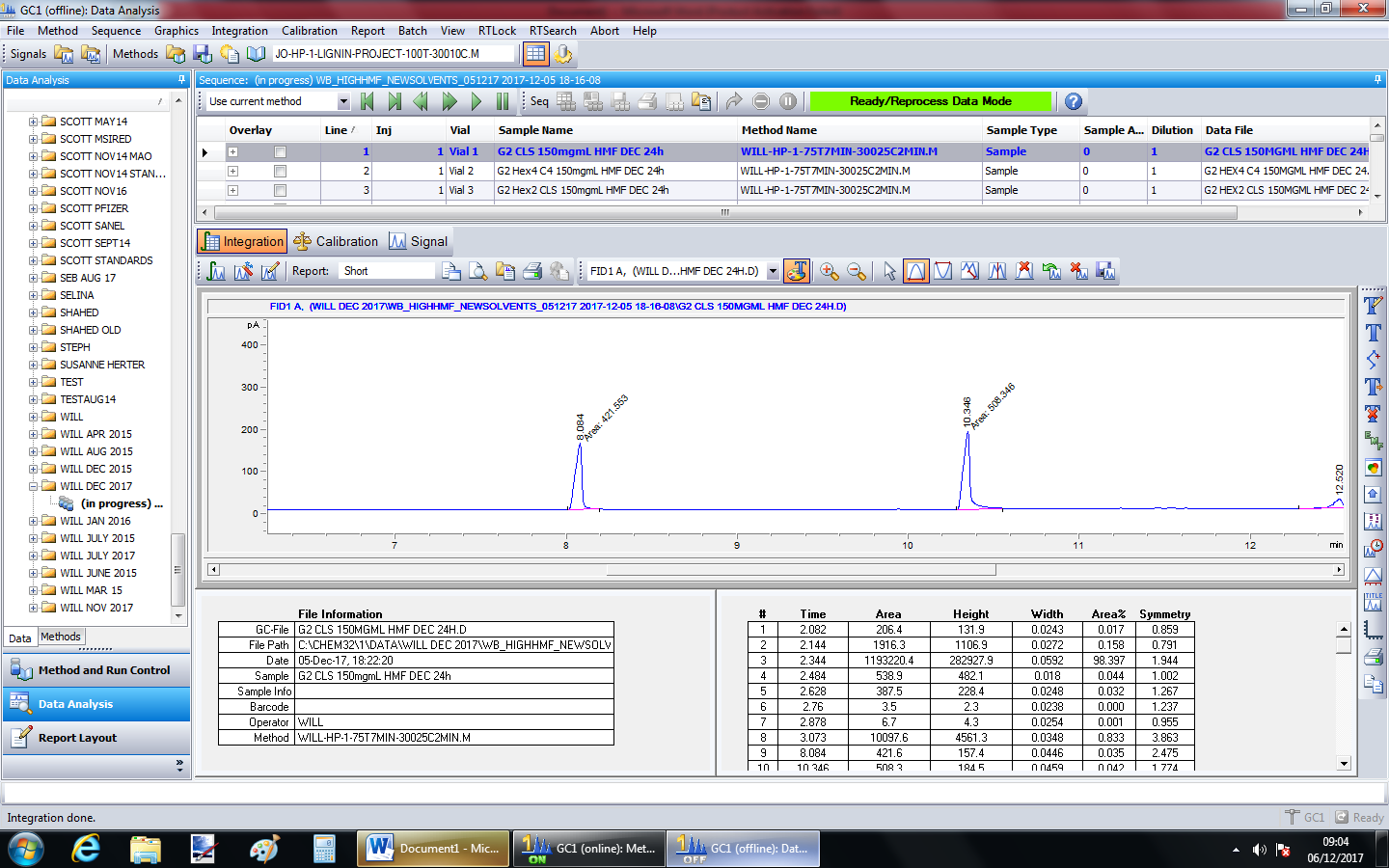


**DFF**

**HMF**

M_6-B_ 150 g/L HMF DEC 24h (EtOAc phase, GC)


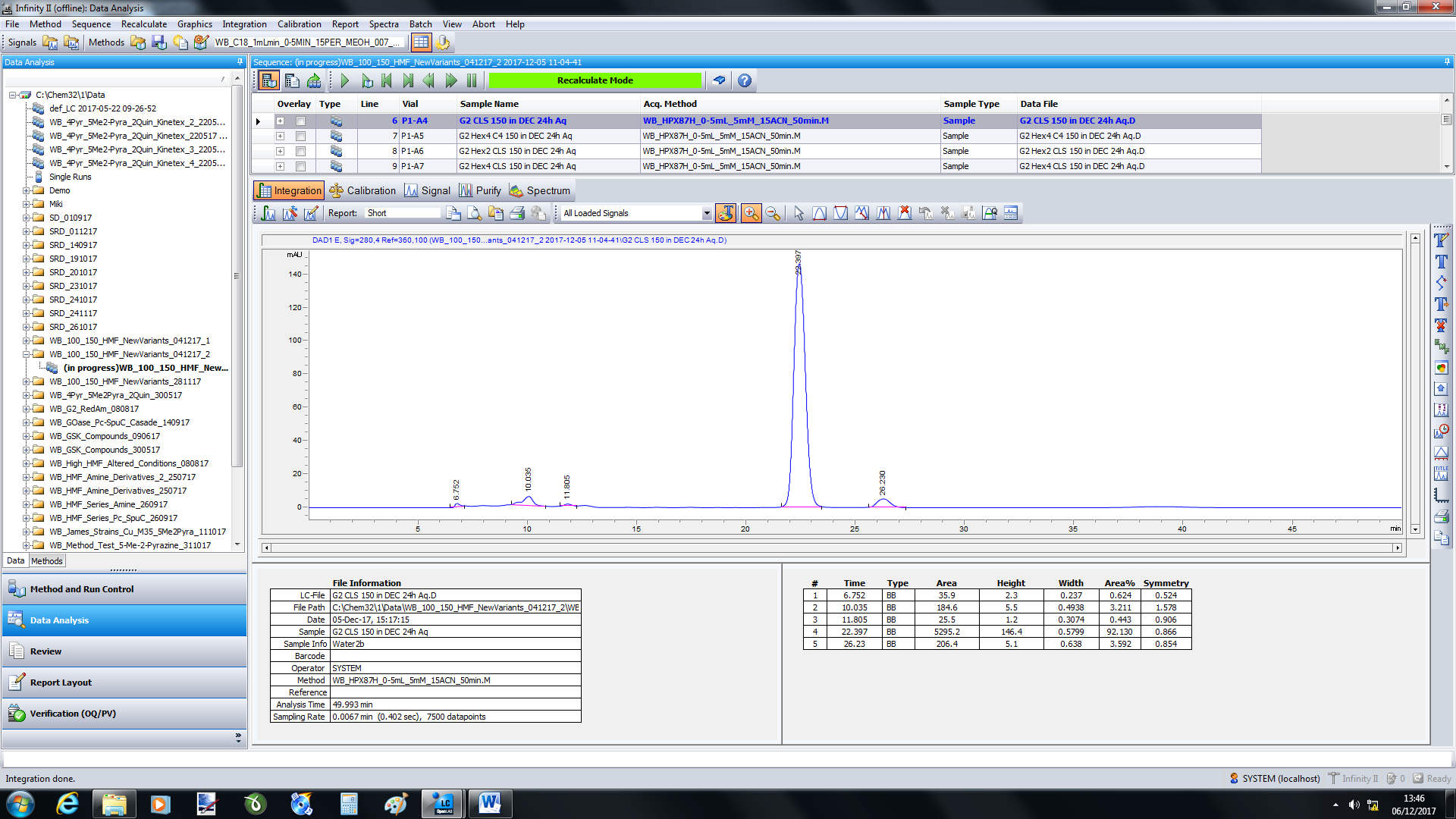


**DFF**

**HMF**

M_6-B_ 150 g/L HMF DEC 24h (Aqueous phase, HPLC)


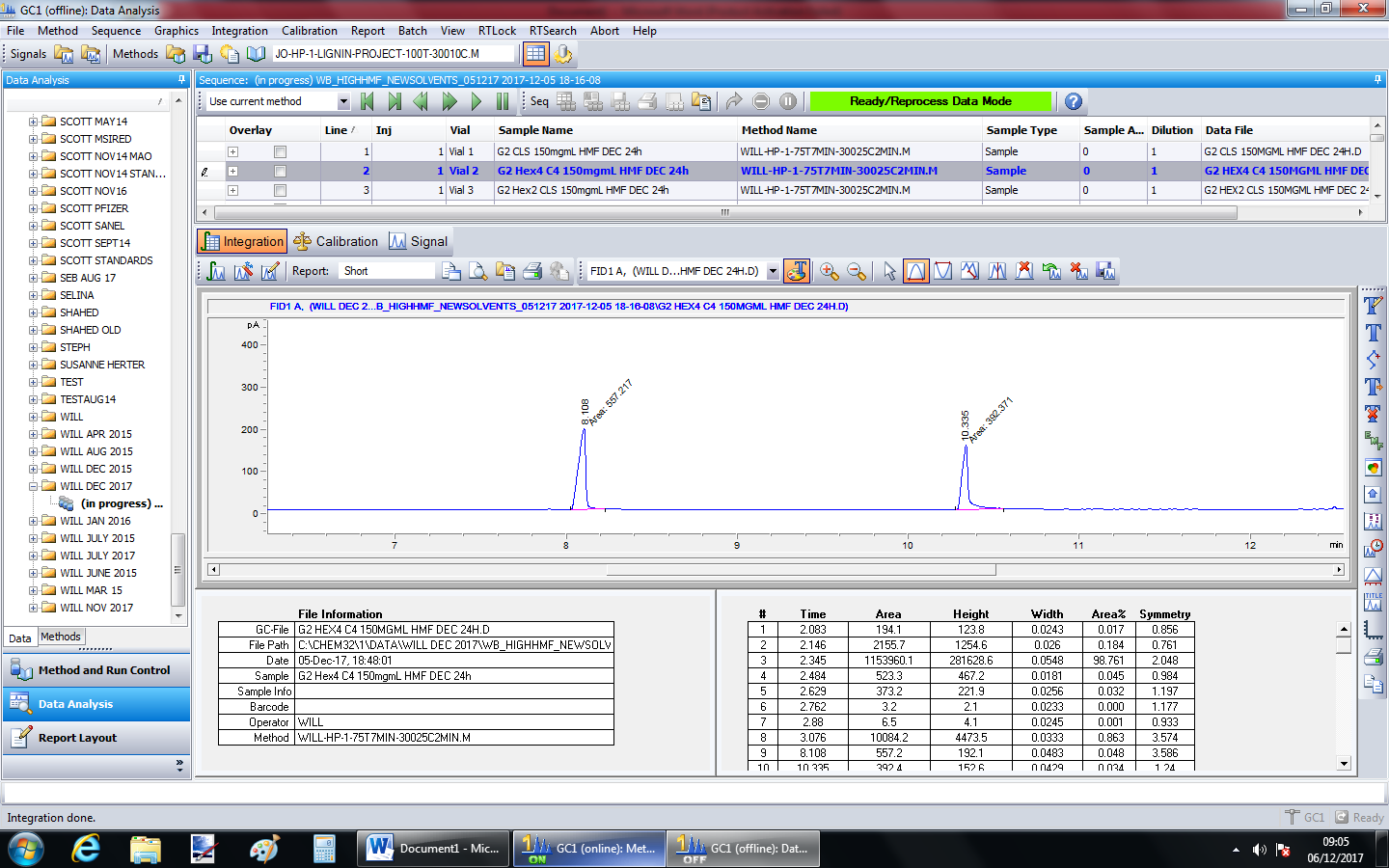


M_7-2A_ 150 g/L HMF DEC 24h (EtOAc phase, GC)


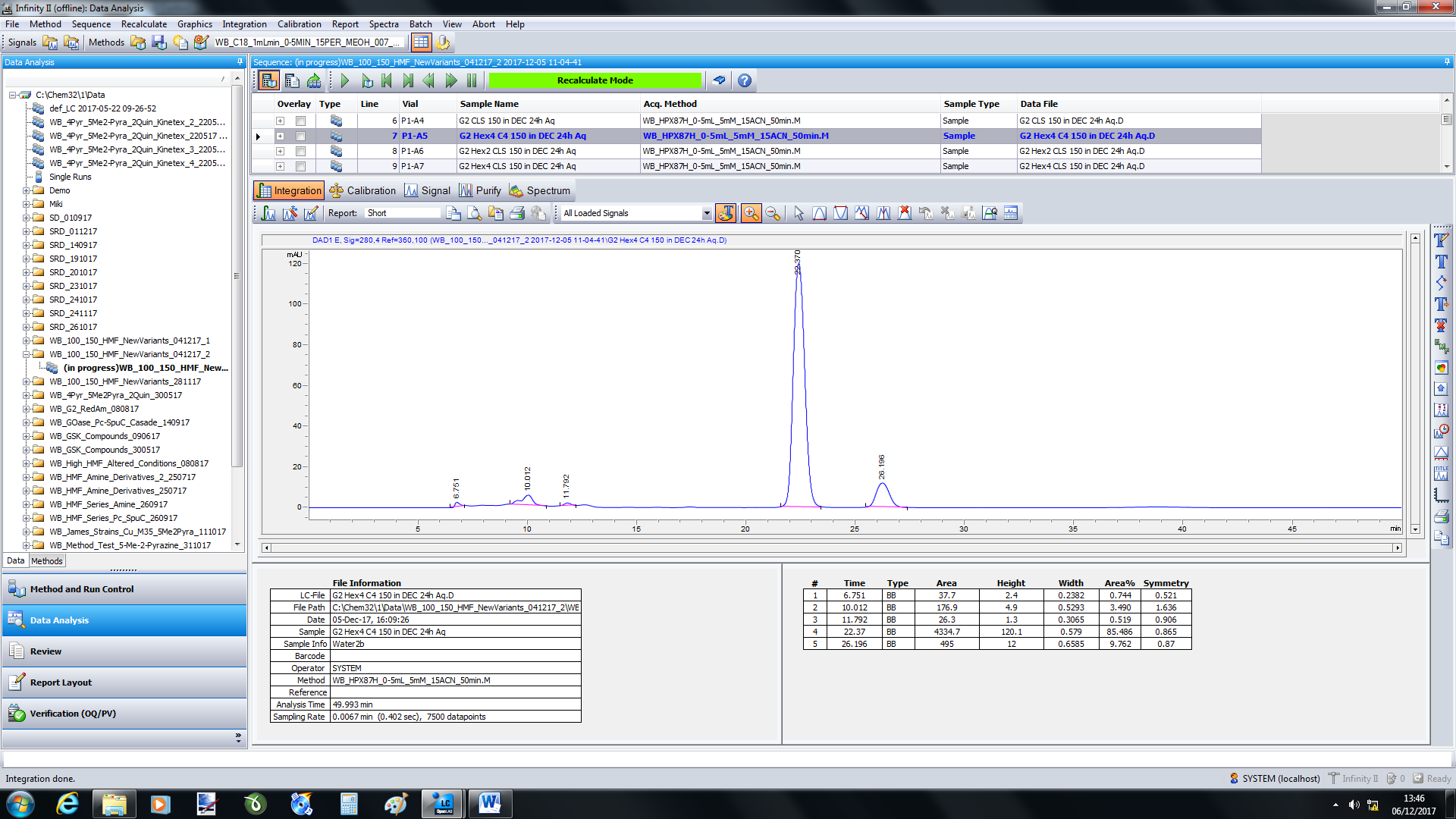


M_7-2A_ 150 g/L HMF DEC 24h (Aqueous phase, HPLC)


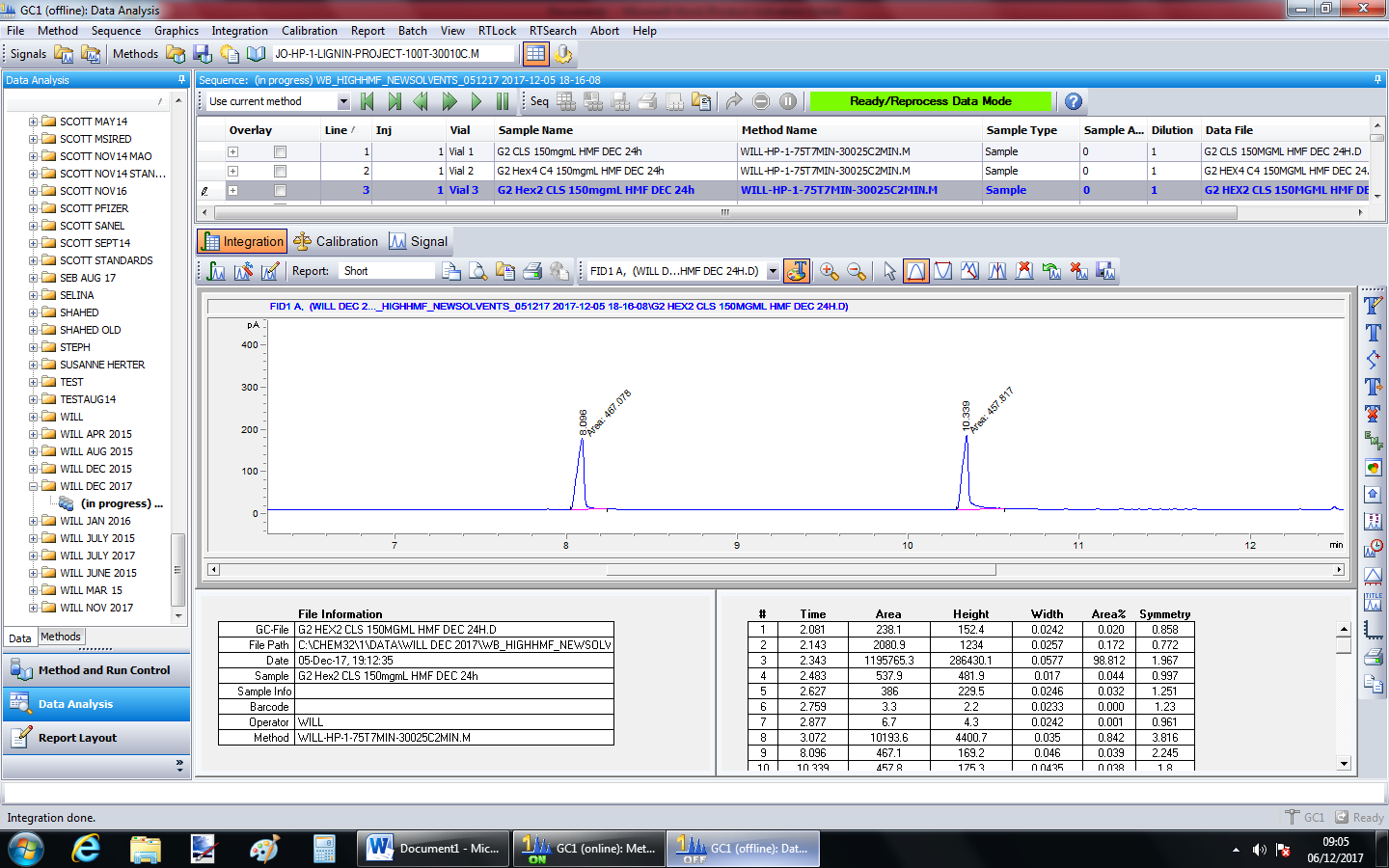


M_7-1B_ 150 g/L HMF DEC 24h (EtOAc phase, GC)


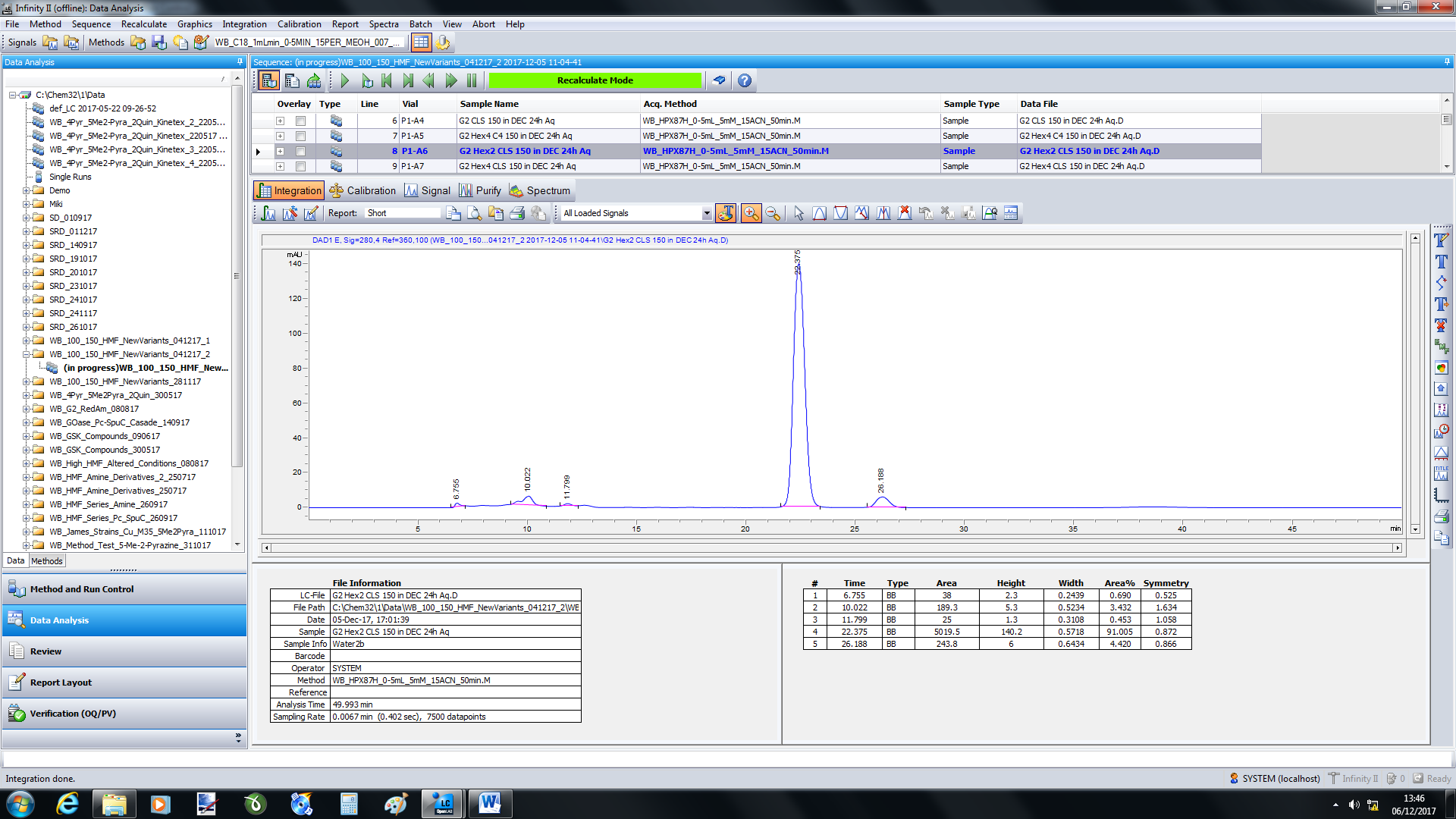


M_7-1B_ 150 g/L HMF DEC 24h (Aqueous phase, HPLC)


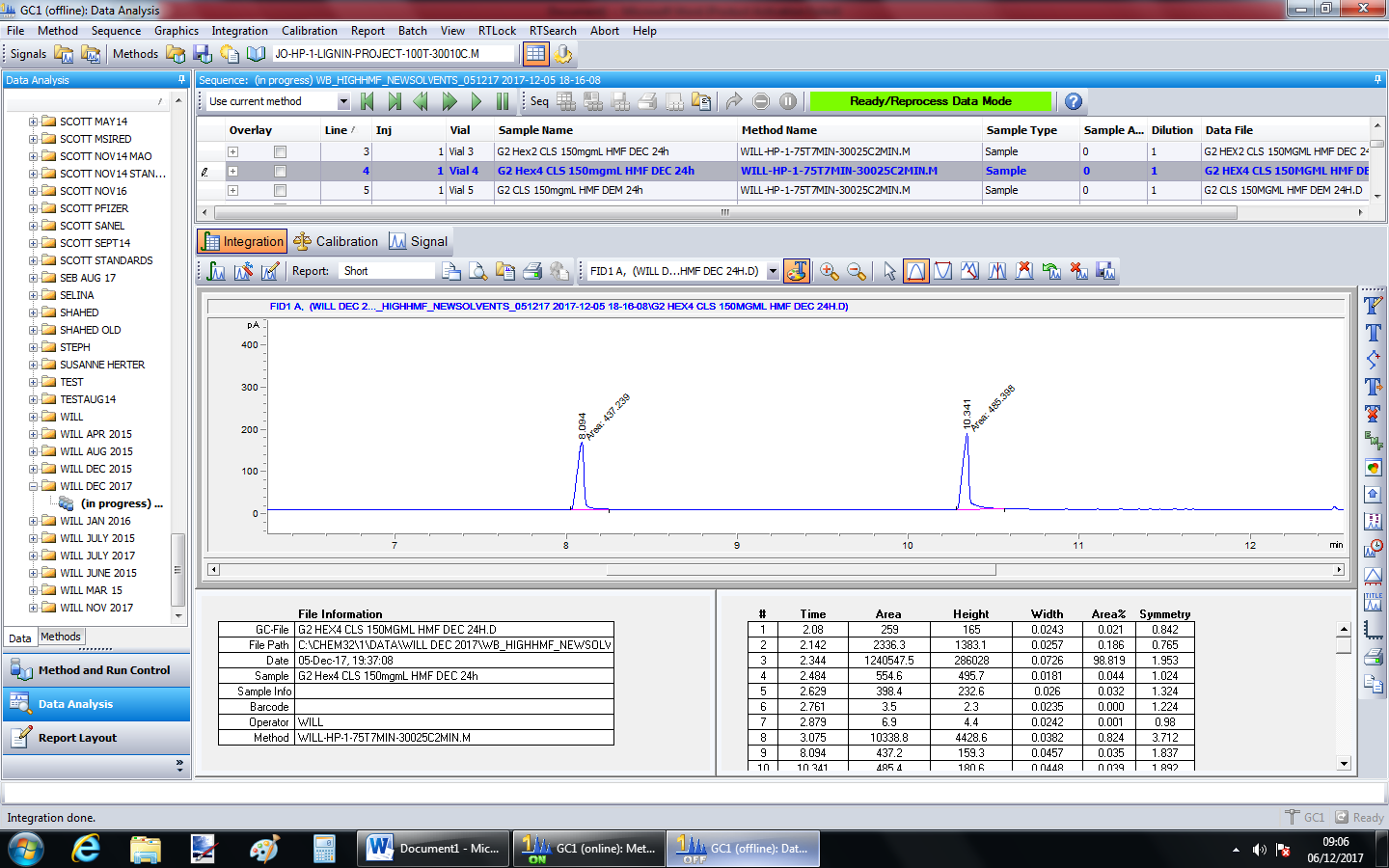


M_7-2B_ 150 g/L HMF DEC 24h (EtOAc phase, GC)


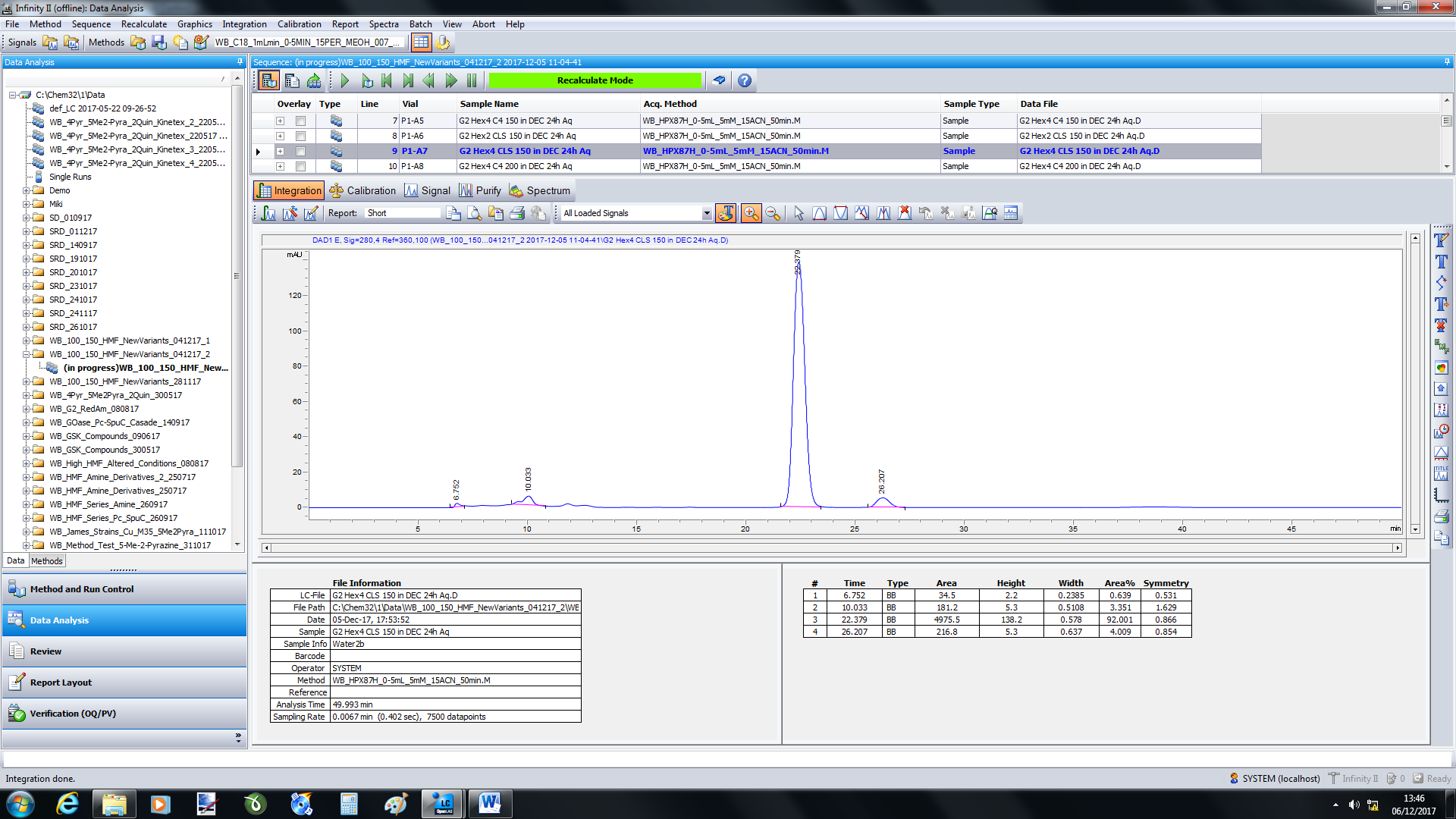


M_7-2B_ 150 g/L HMF DEC 24h (Aqueous phase, HPLC)


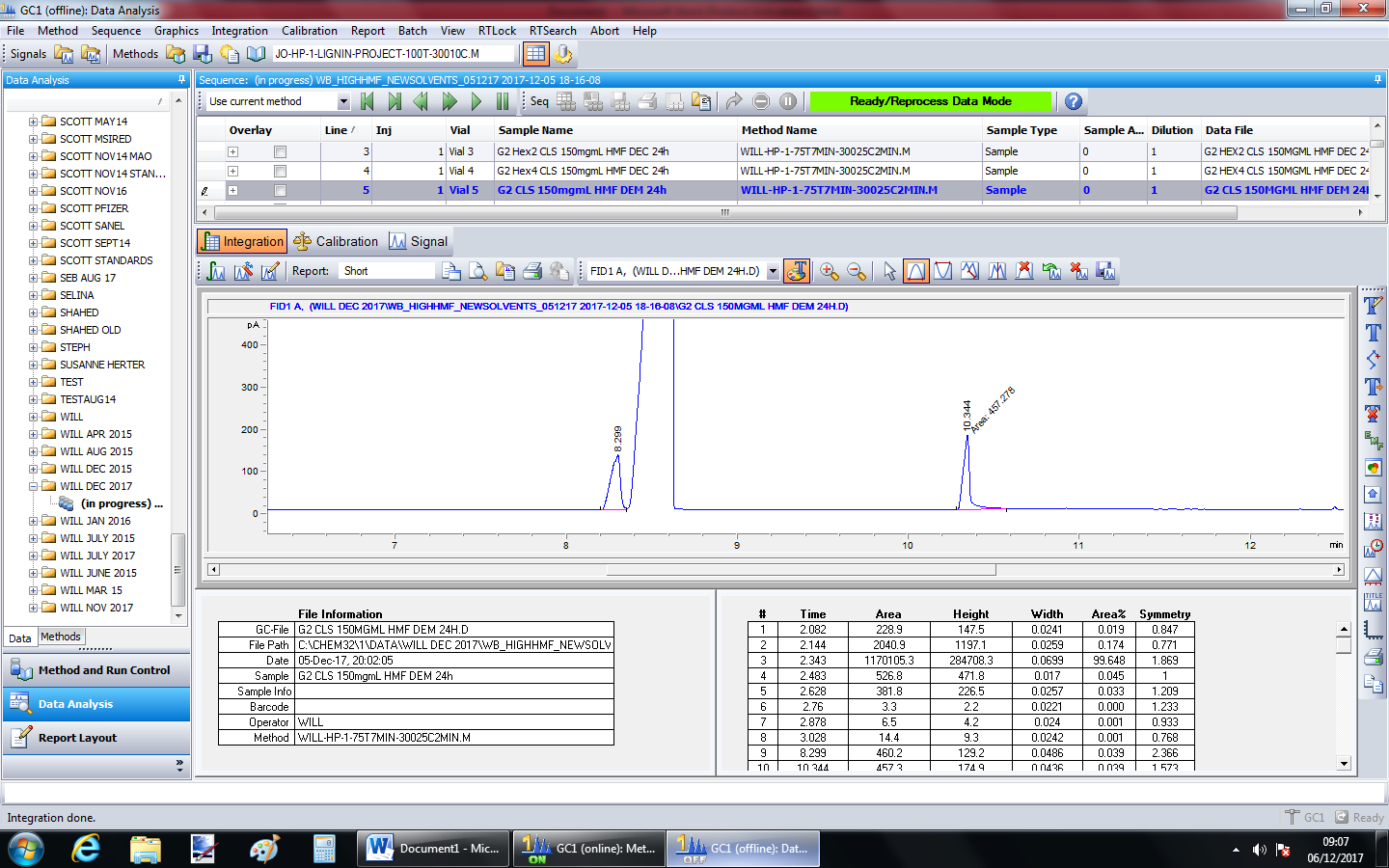


M_6-B_ 150 g/L HMF DEM 24h (EtOAc phase, GC)


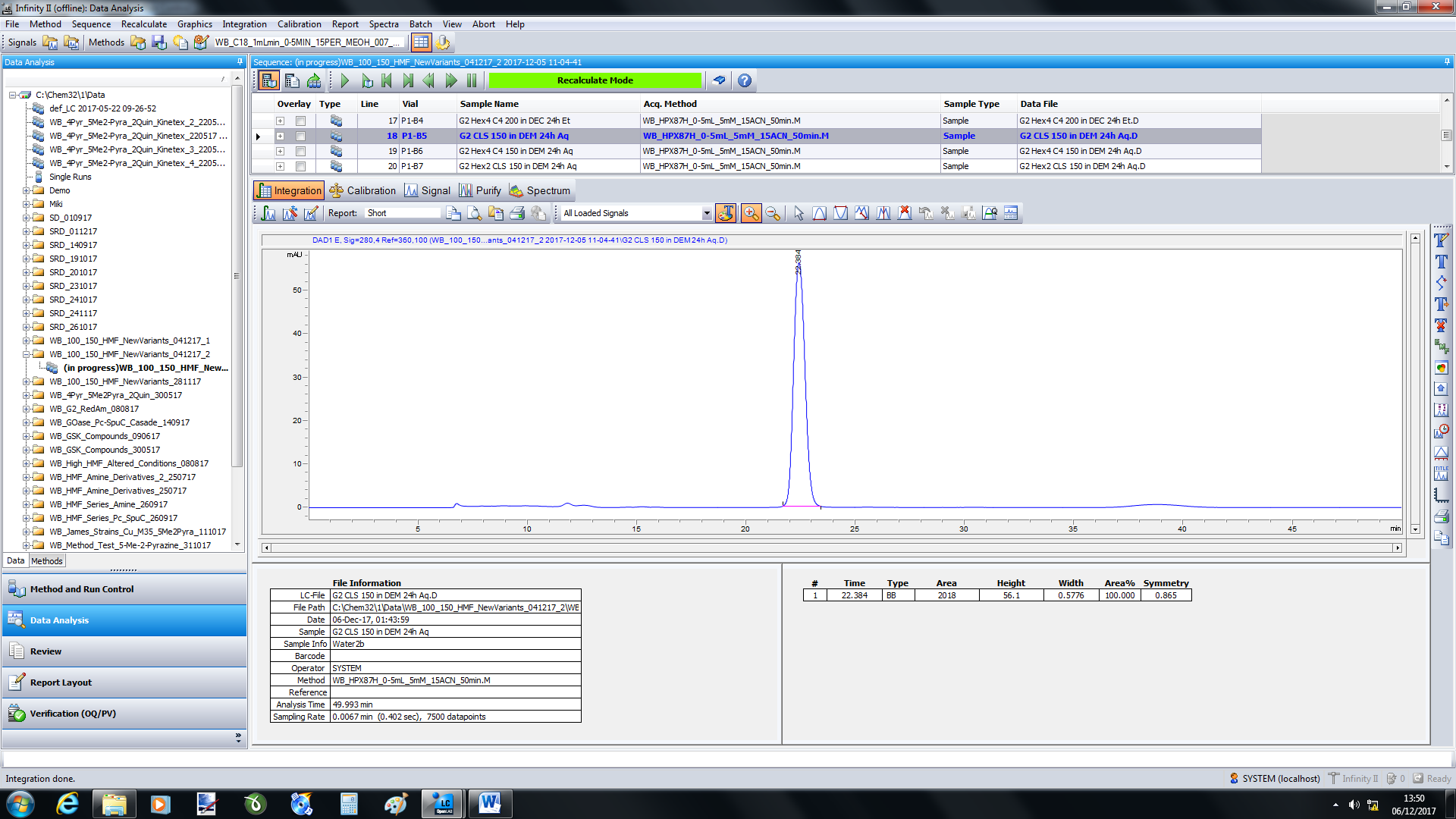


M_6-B_ 150 g/L HMF DEM 24h (Aqueous phase, HPLC)


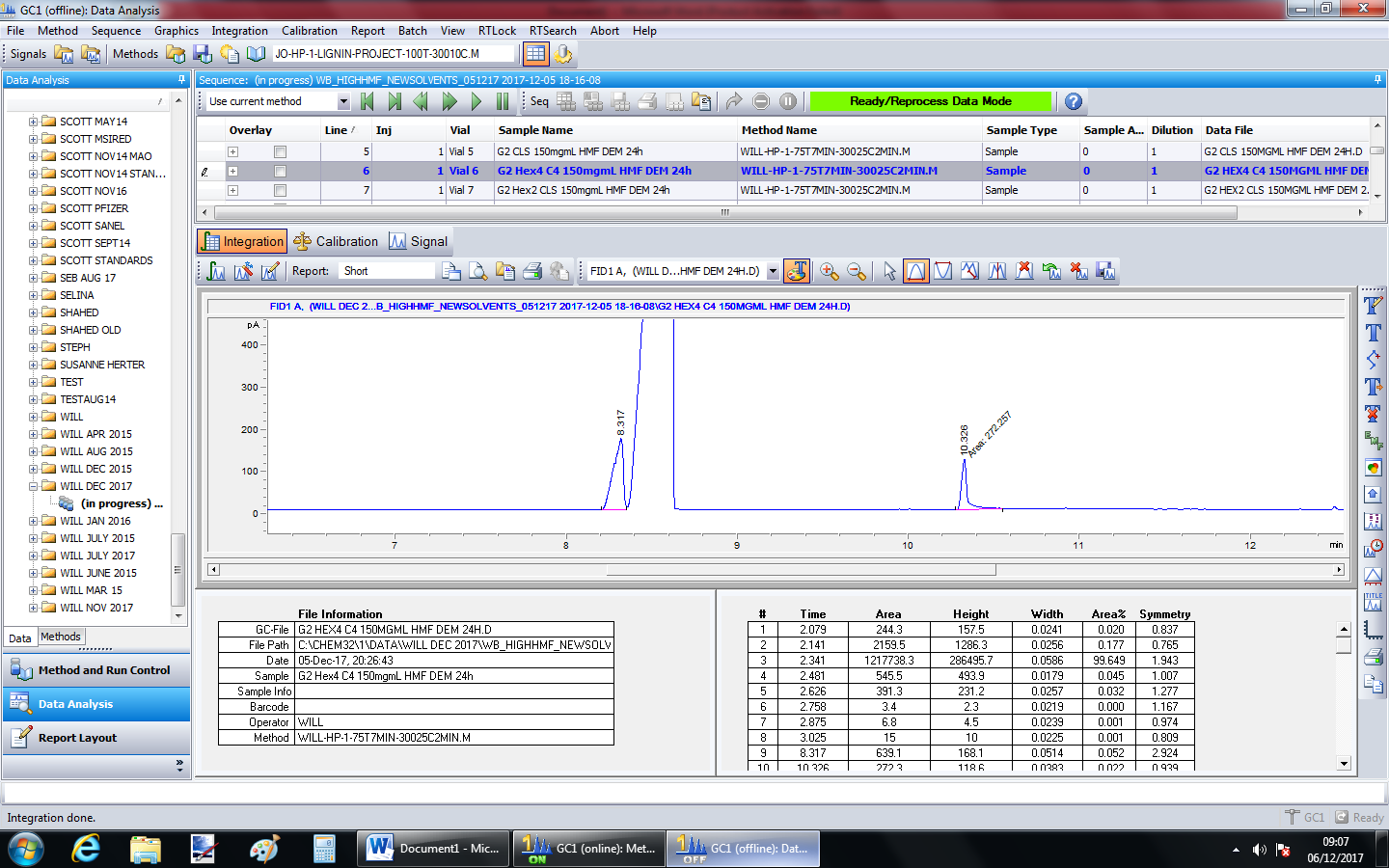


M_7-2A_ 150 g/L HMF DEM 24h (EtOAc phase, GC)


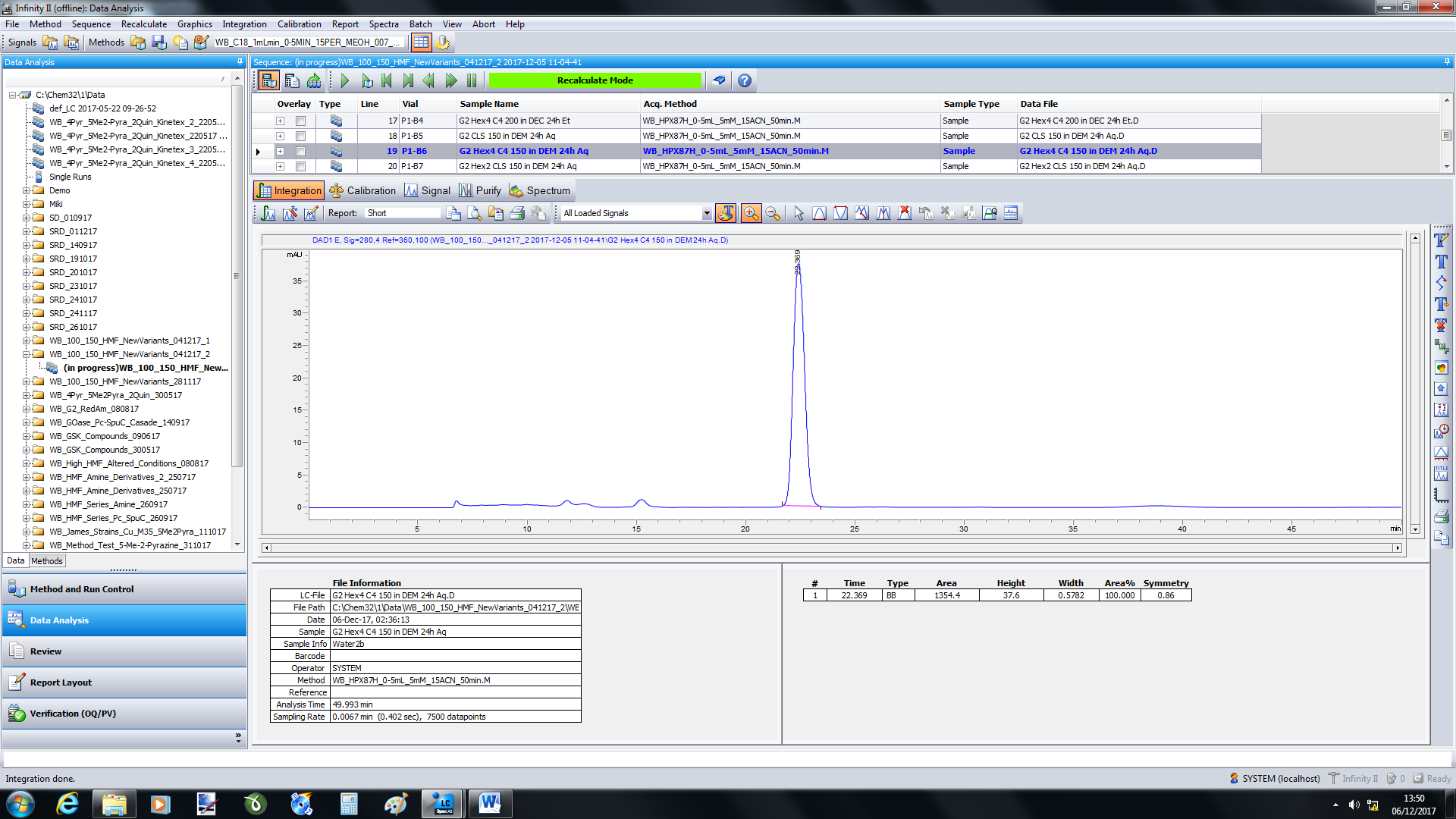


M_7-2A_ 150 g/L HMF DEM 24h (Aqueous phase, HPLC)


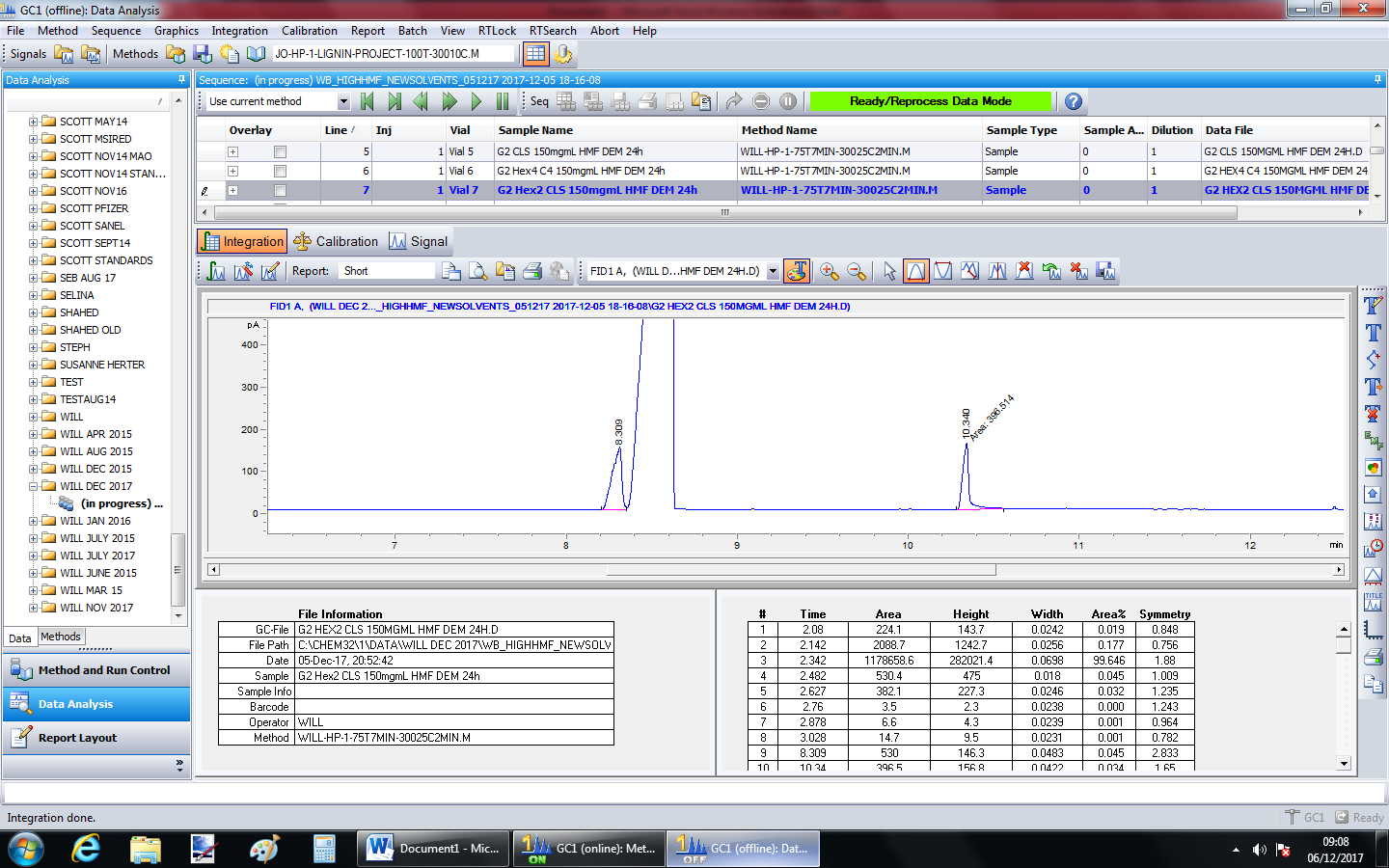


M_7-1B_ 150 g/L HMF DEM 24h (EtOAc phase, GC)


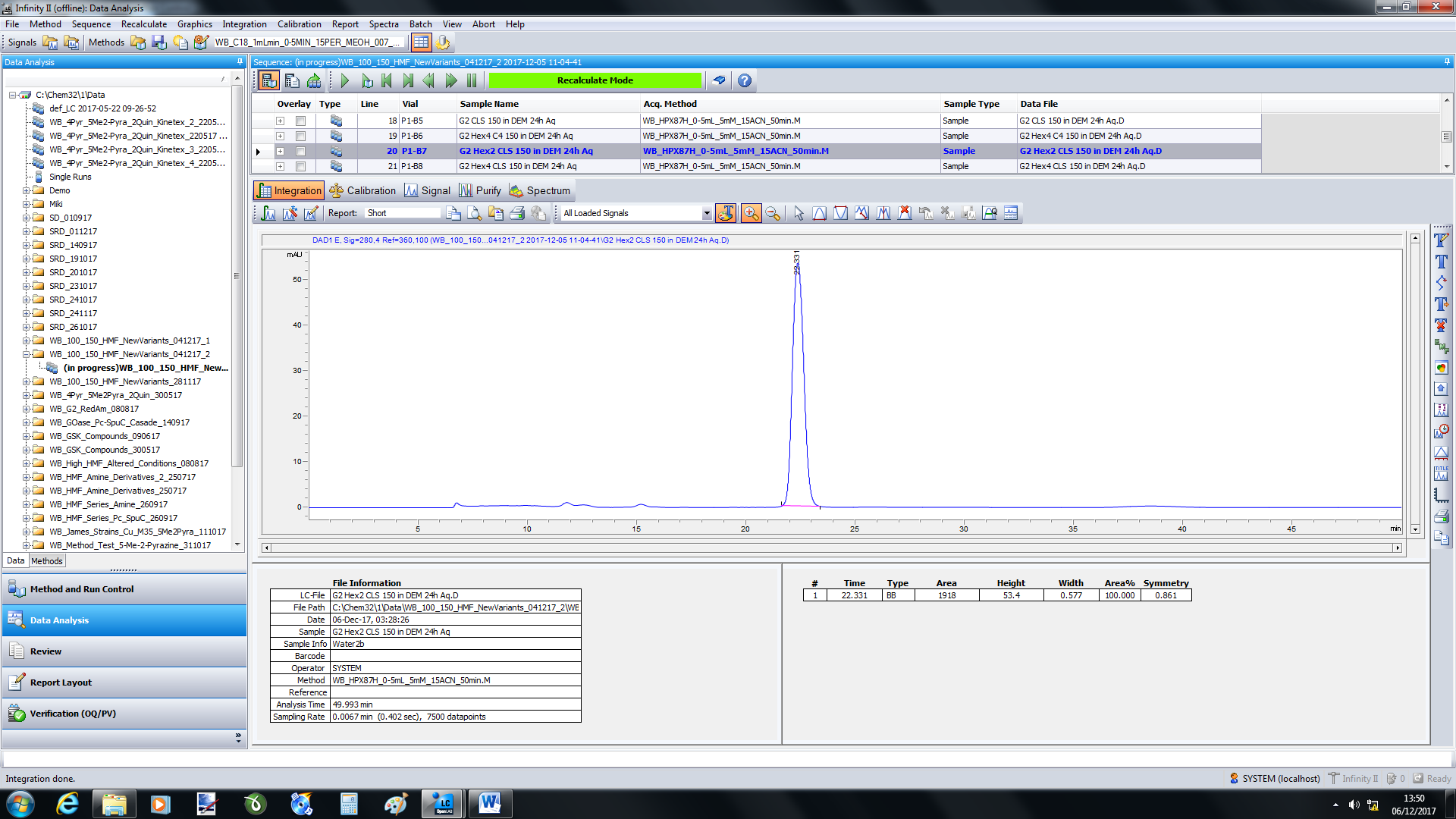


M_7-1B_ 150 g/L HMF DEM 24h (Aqueous phase, HPLC)


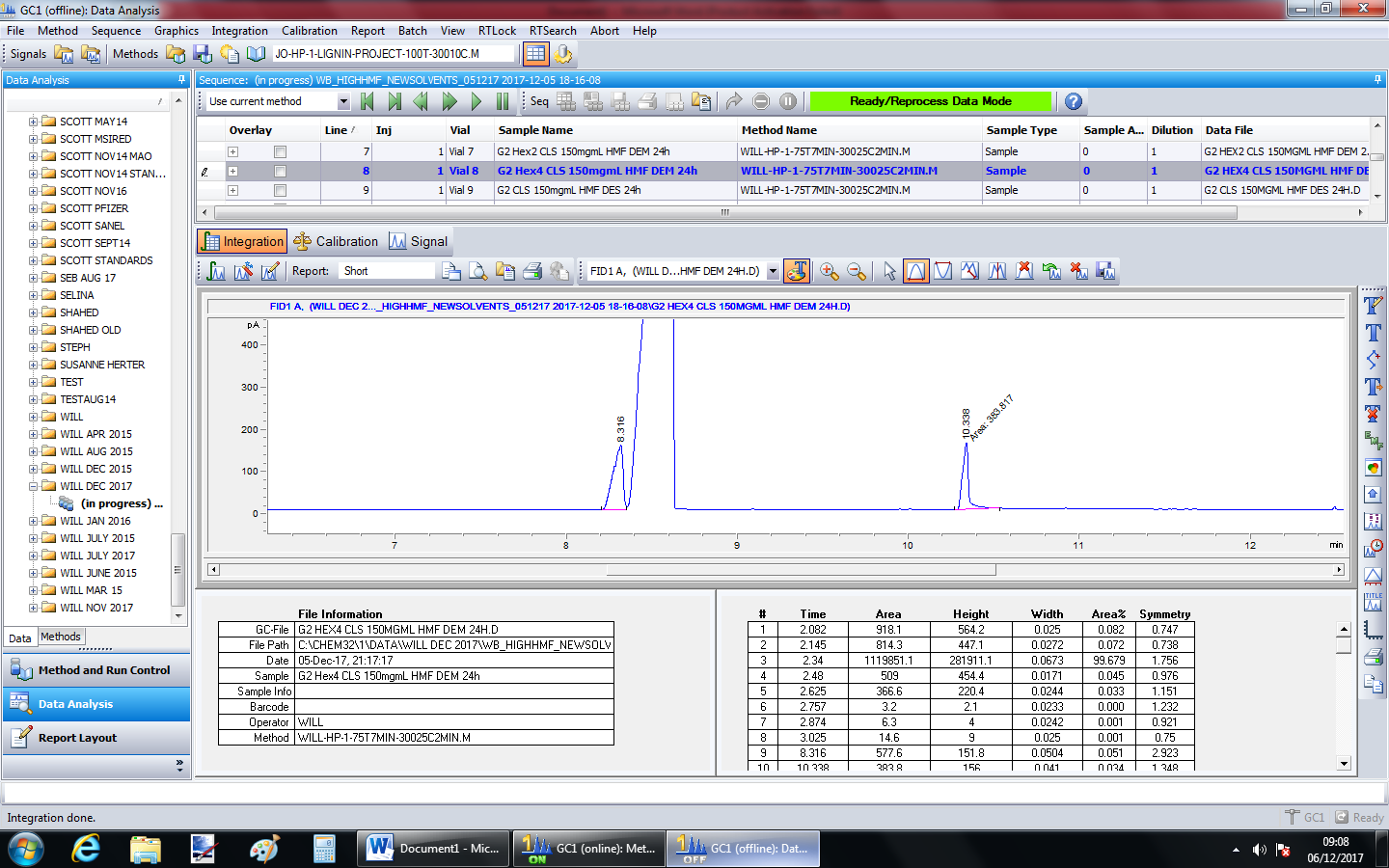


M_7-2B_ 150 g/L HMF DEM 24h (EtOAc phase, GC)


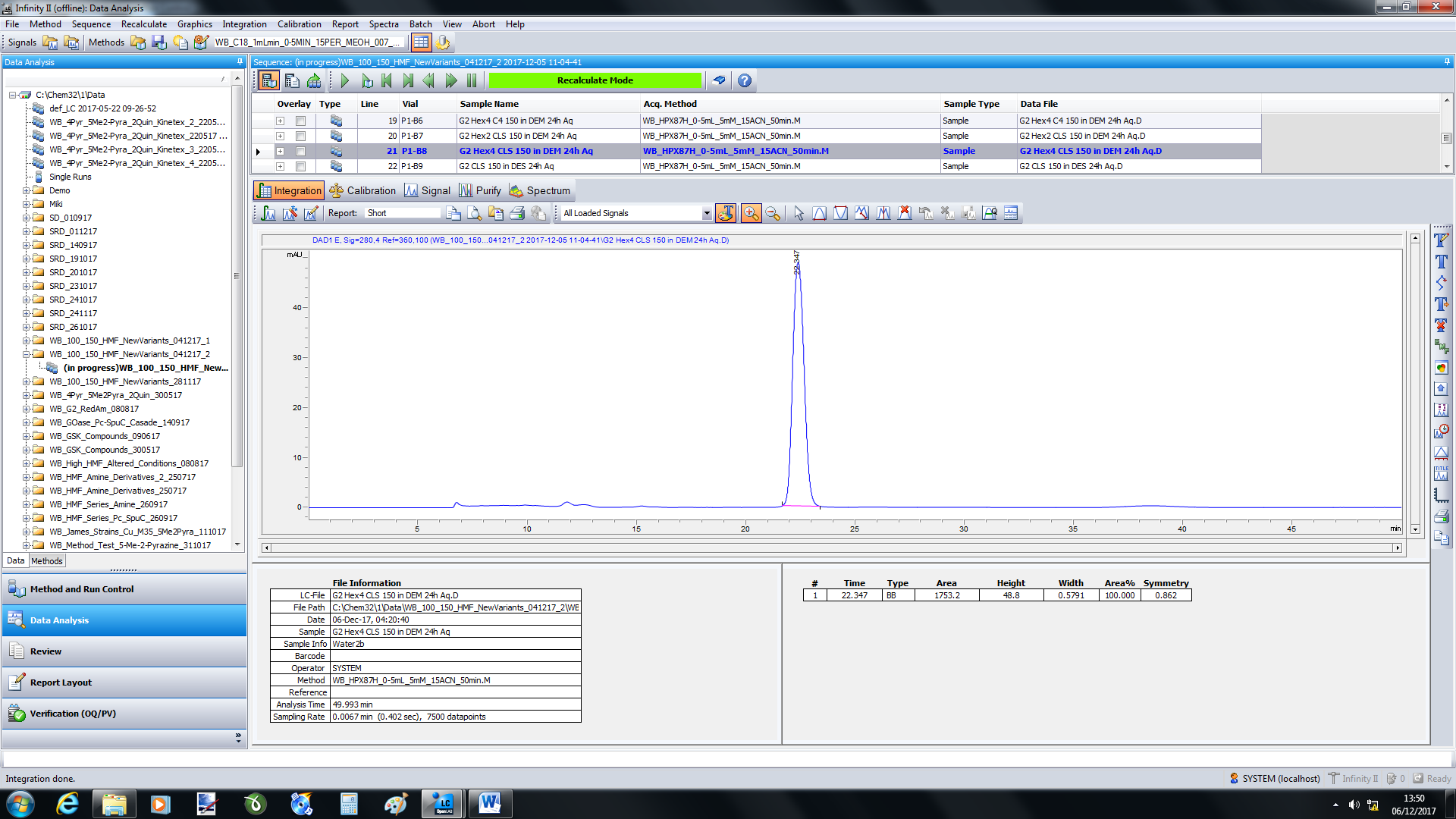


M_7-2B_ 150 g/L HMF DEM 24h (Aqueous phase, HPLC)


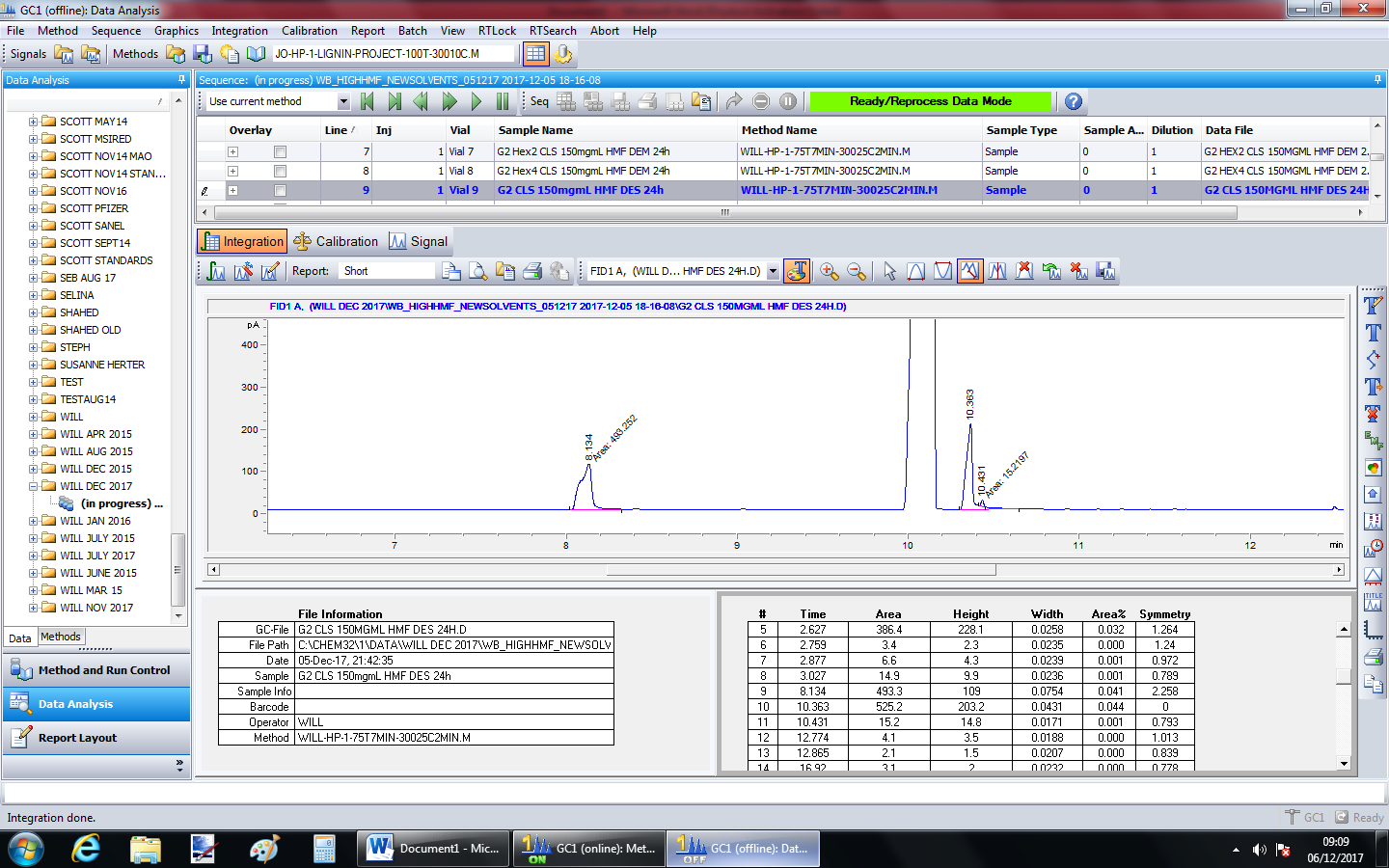


M_6-B_ 150 g/L HMF DES 24h (EtOAc phase, GC)


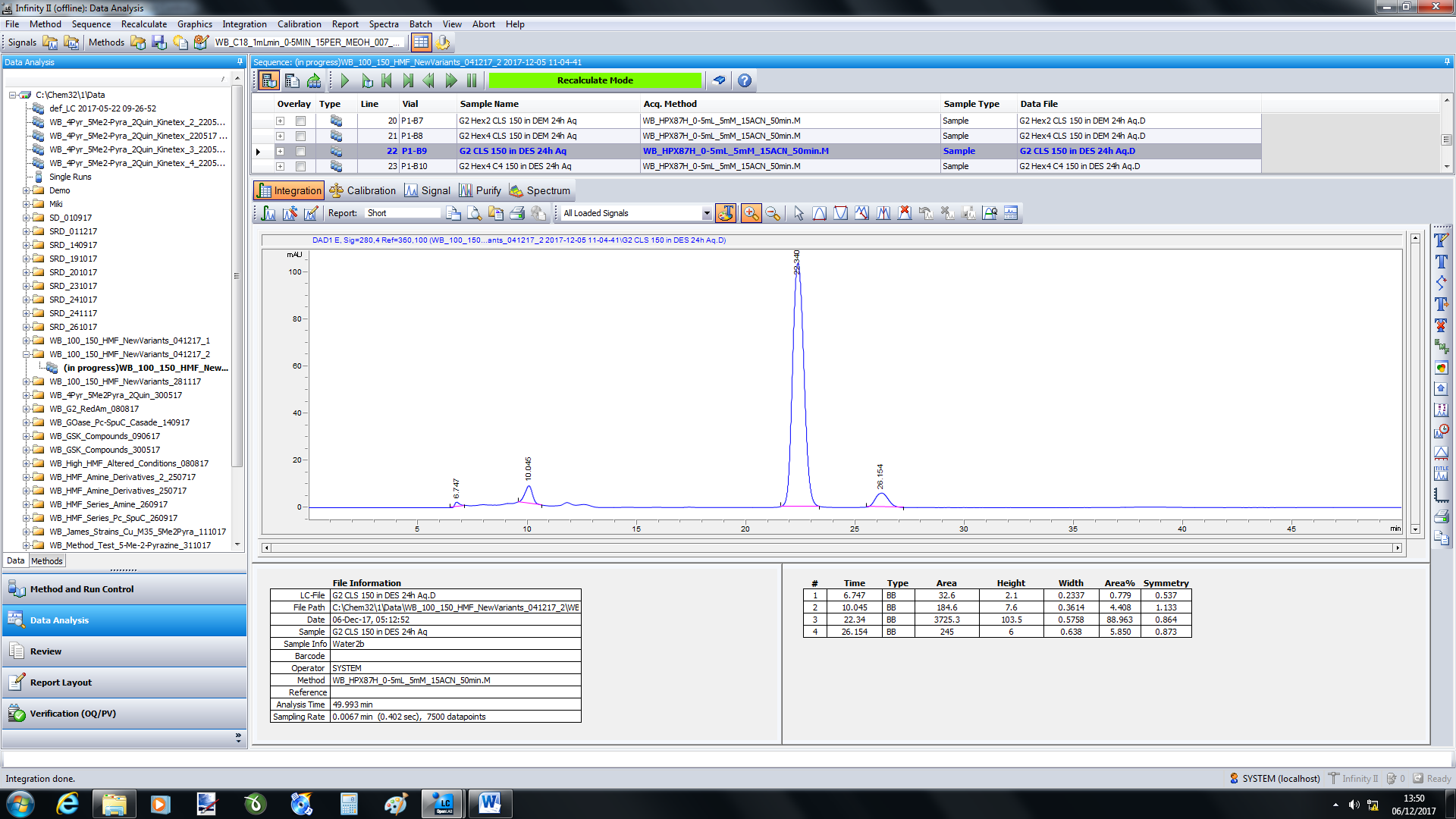


M_6-B_ 150 g/L HMF DES 24h (Aqueous phase, HPLC)


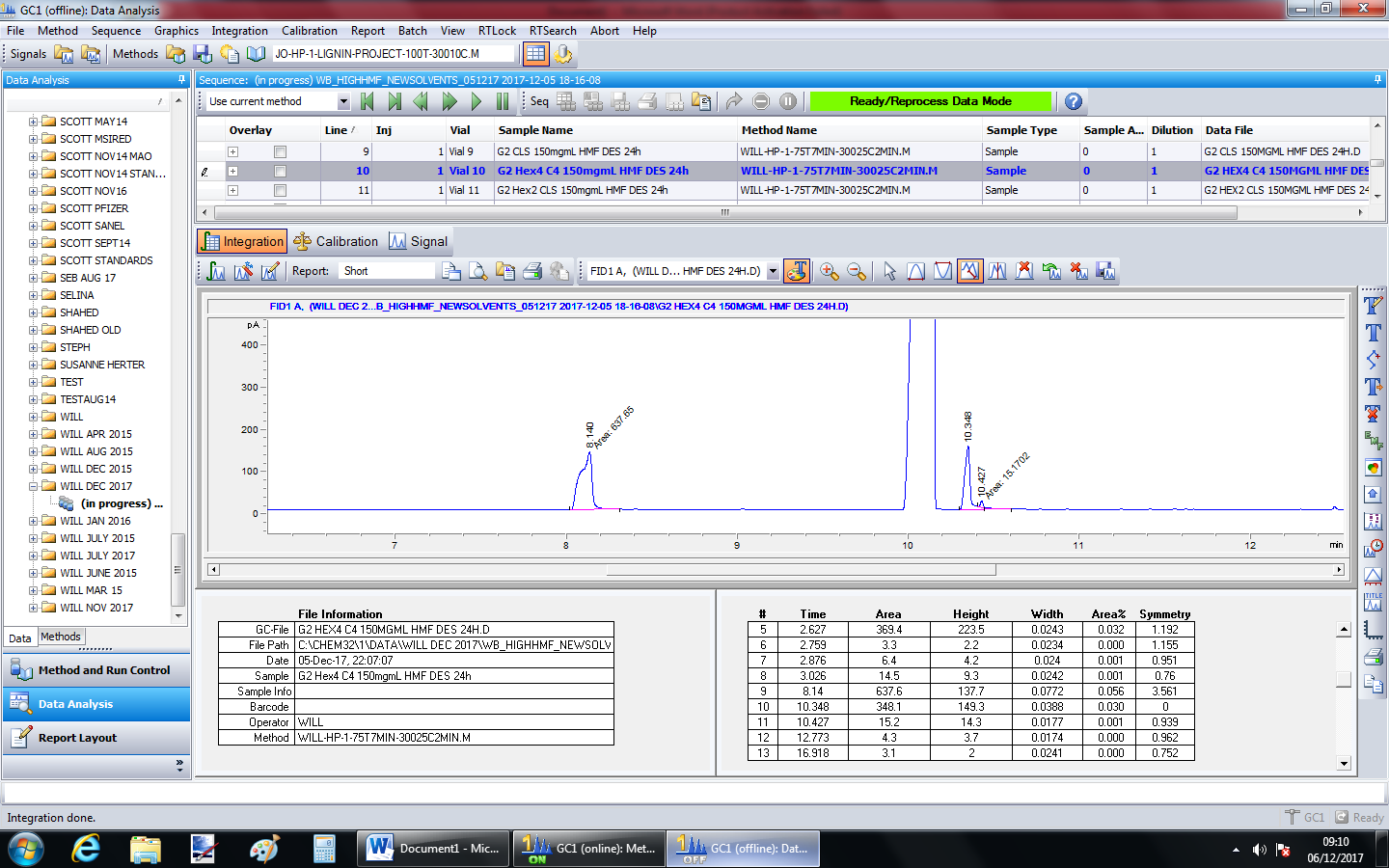


M_7-2A_ 150 g/L HMF DES 24h (EtOAc phase, GC)


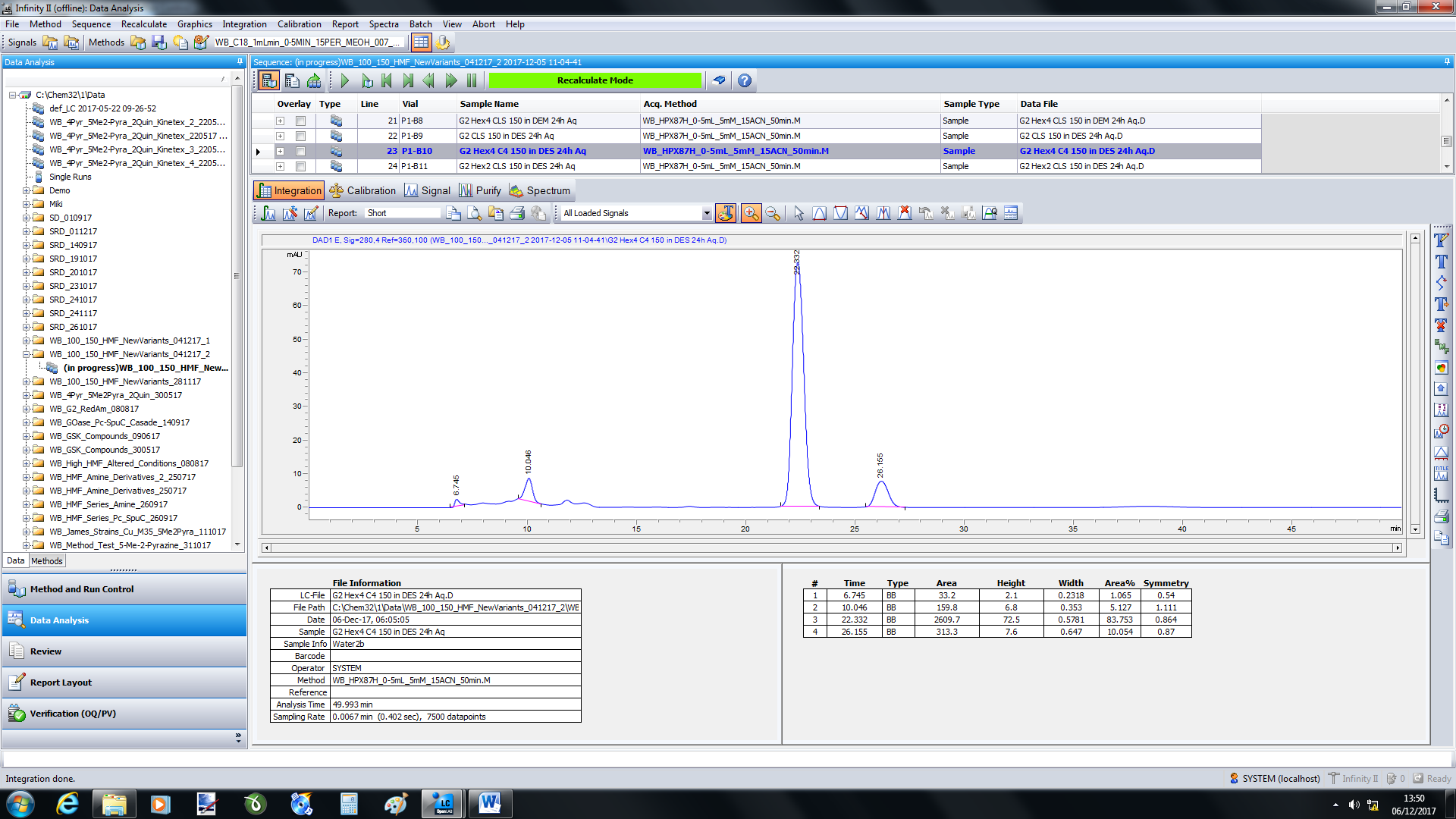


M_7-2A_ 150 g/L HMF DES 24h (Aqueous phase, HPLC)


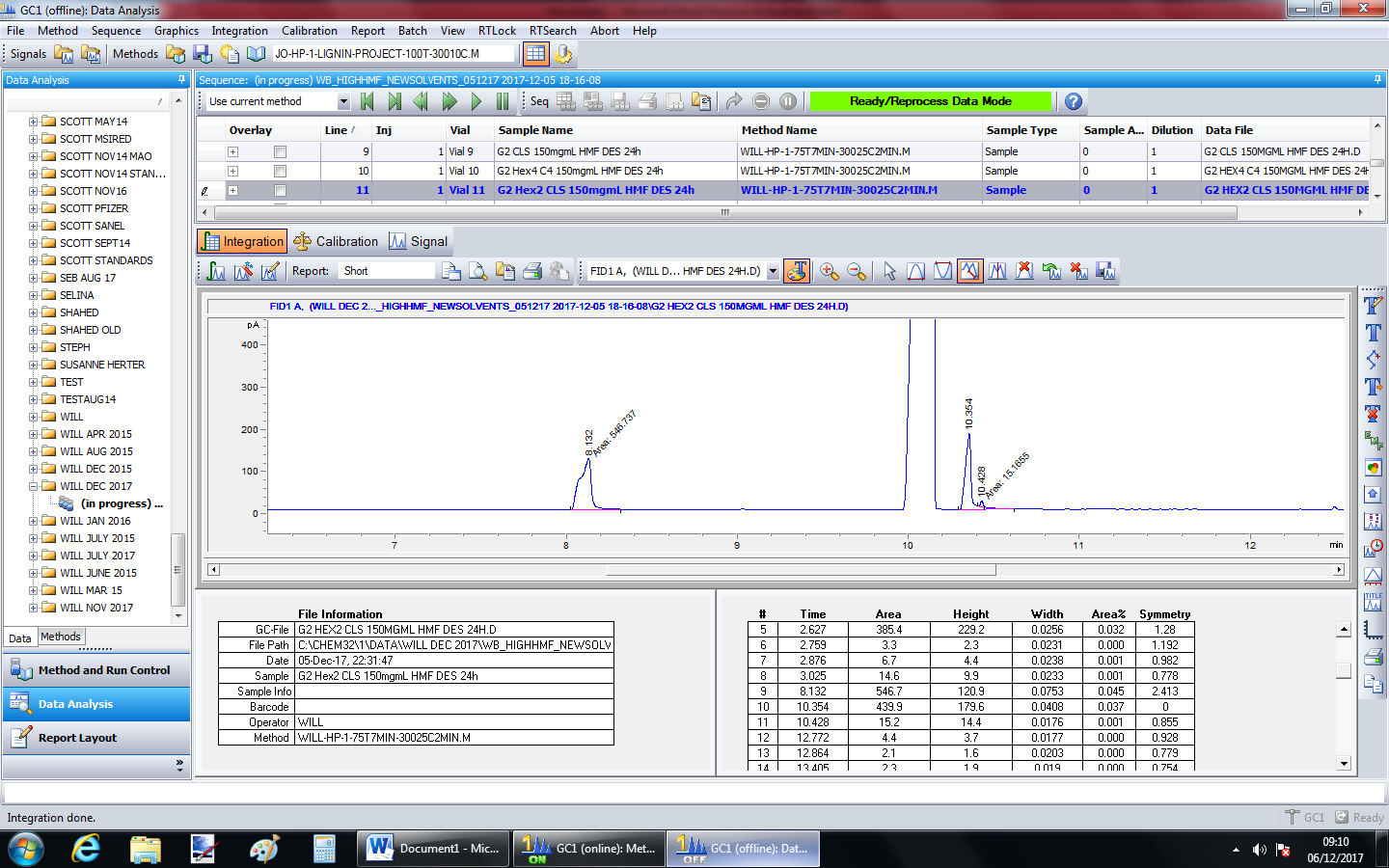


M_7-1B_ 150 g/L HMF DES 24h (EtOAc phase, GC)


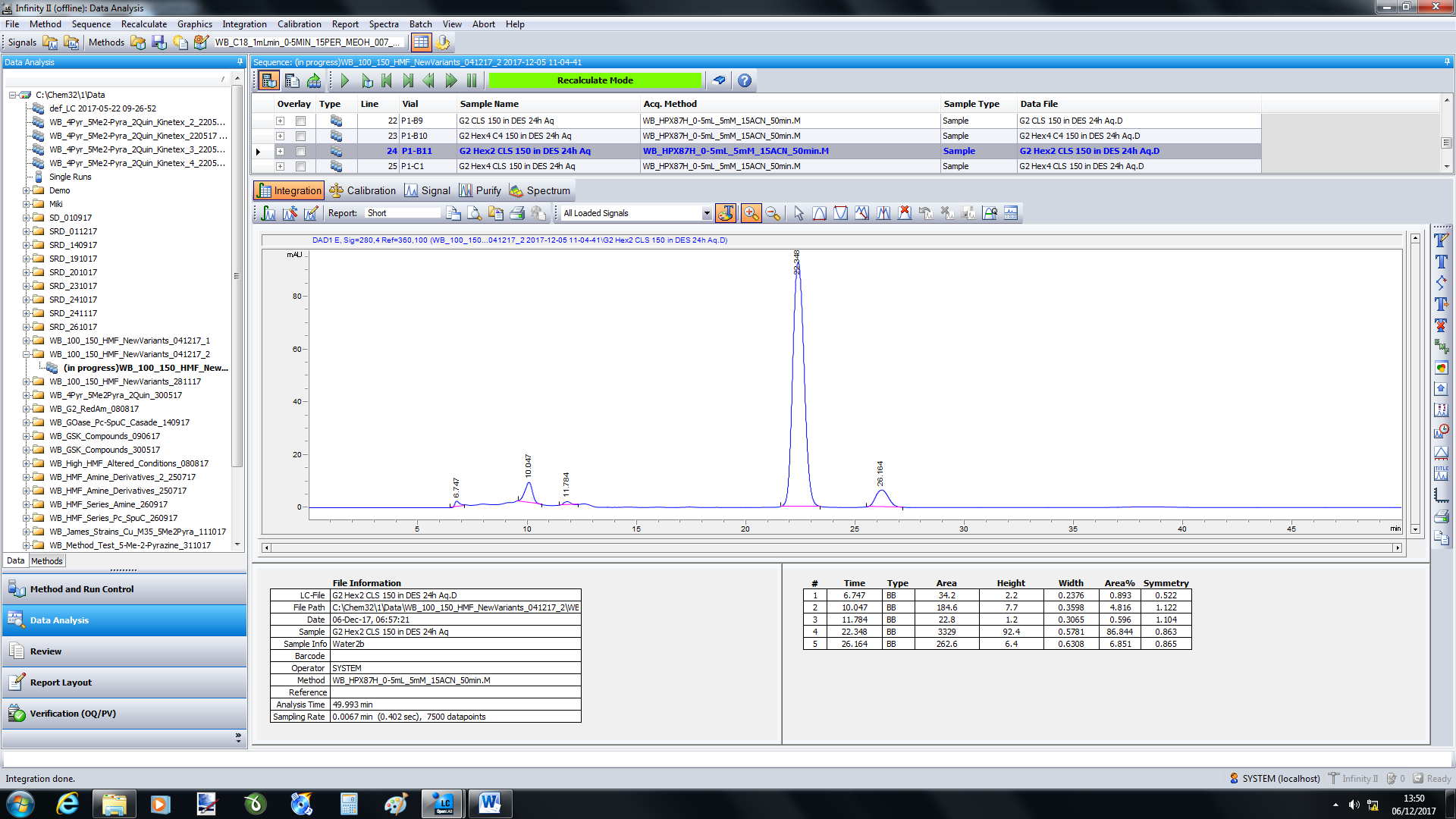


M_7-1B_ 150 g/L HMF DES 24h (Aqueous phase, HPLC)


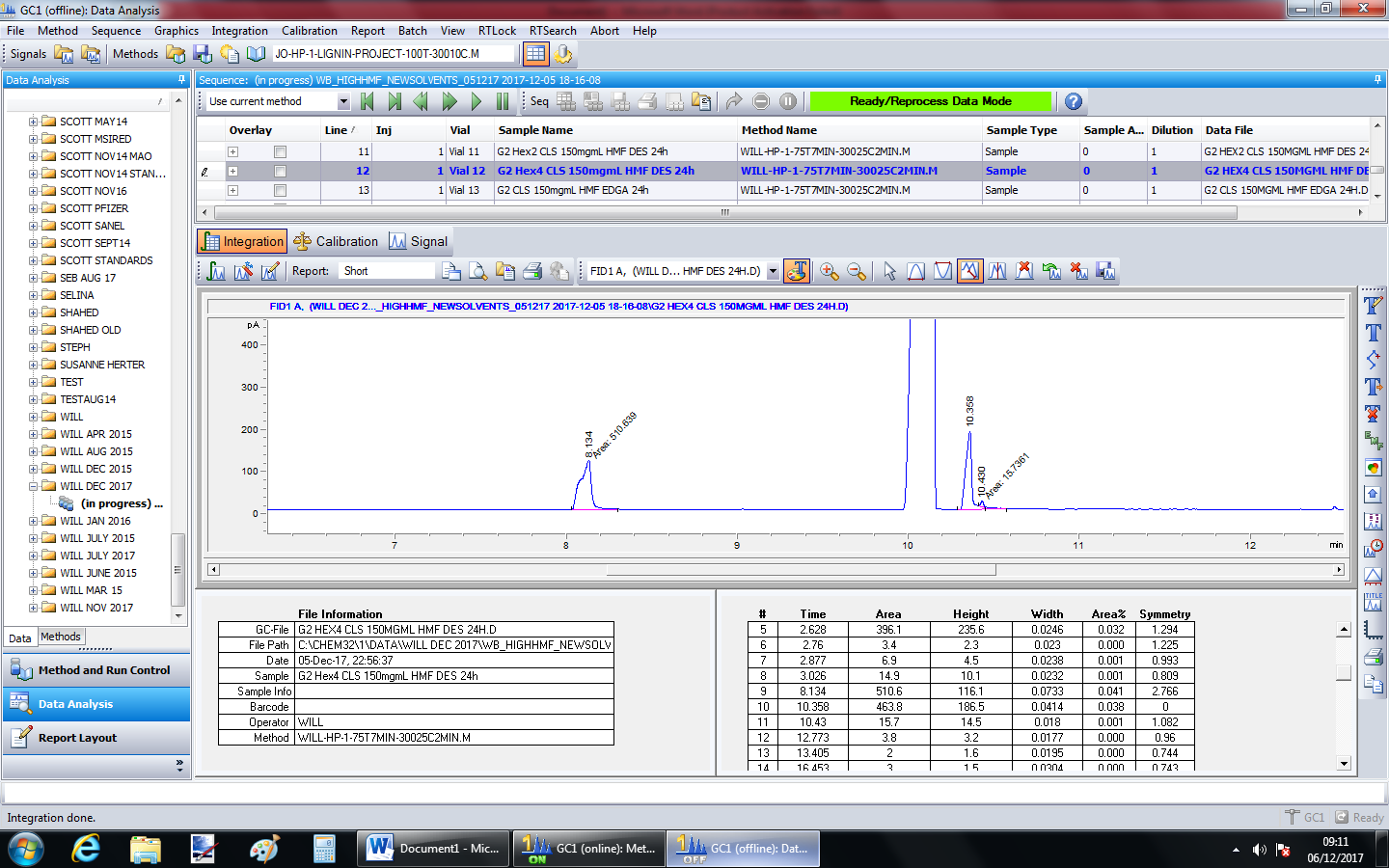


M_7-2B_ 150 g/L HMF DES 24h (EtOAc phase, GC)


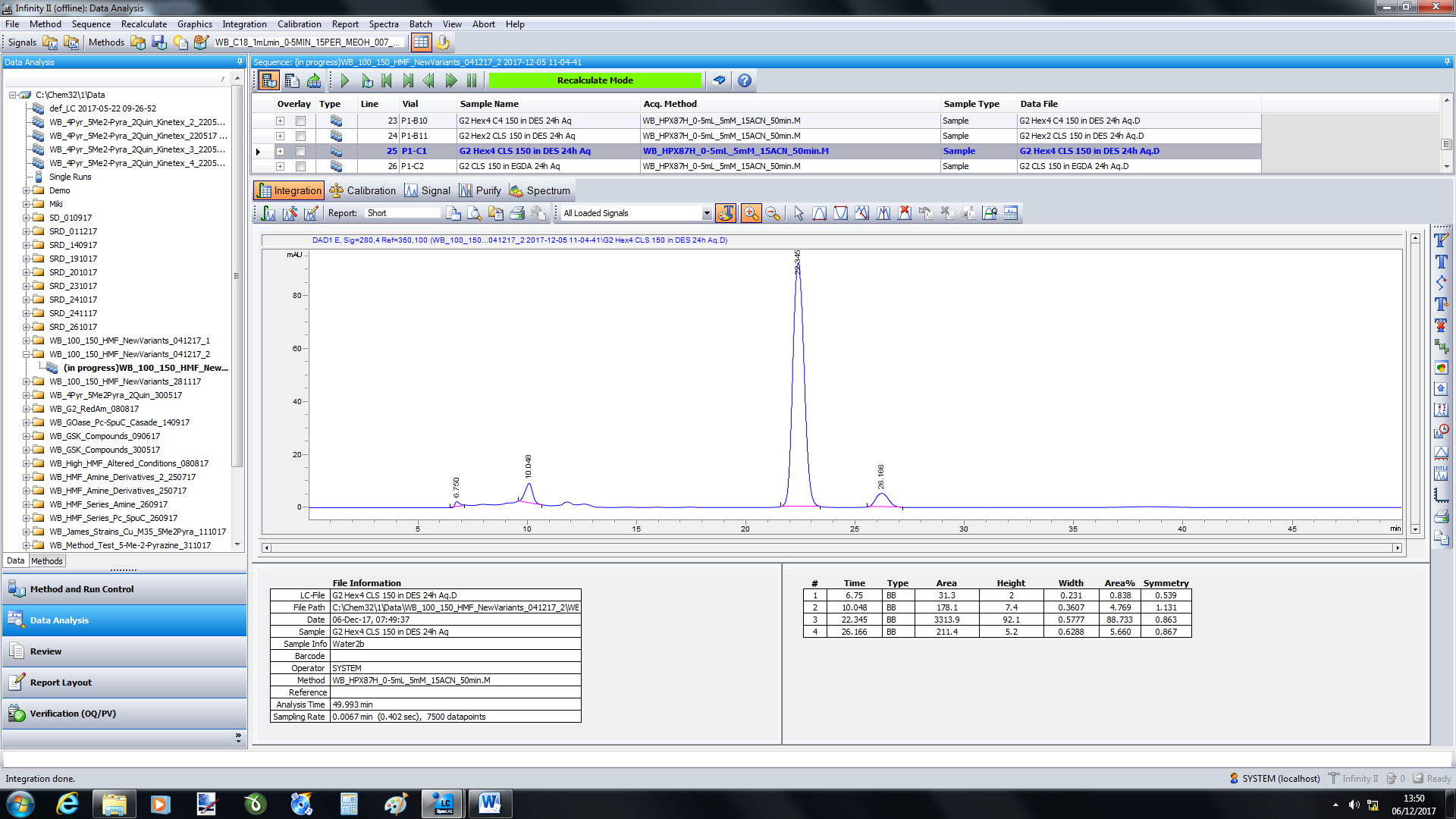


M_7-2B_ 150 g/L HMF DES 24h (Aqueous phase, HPLC)


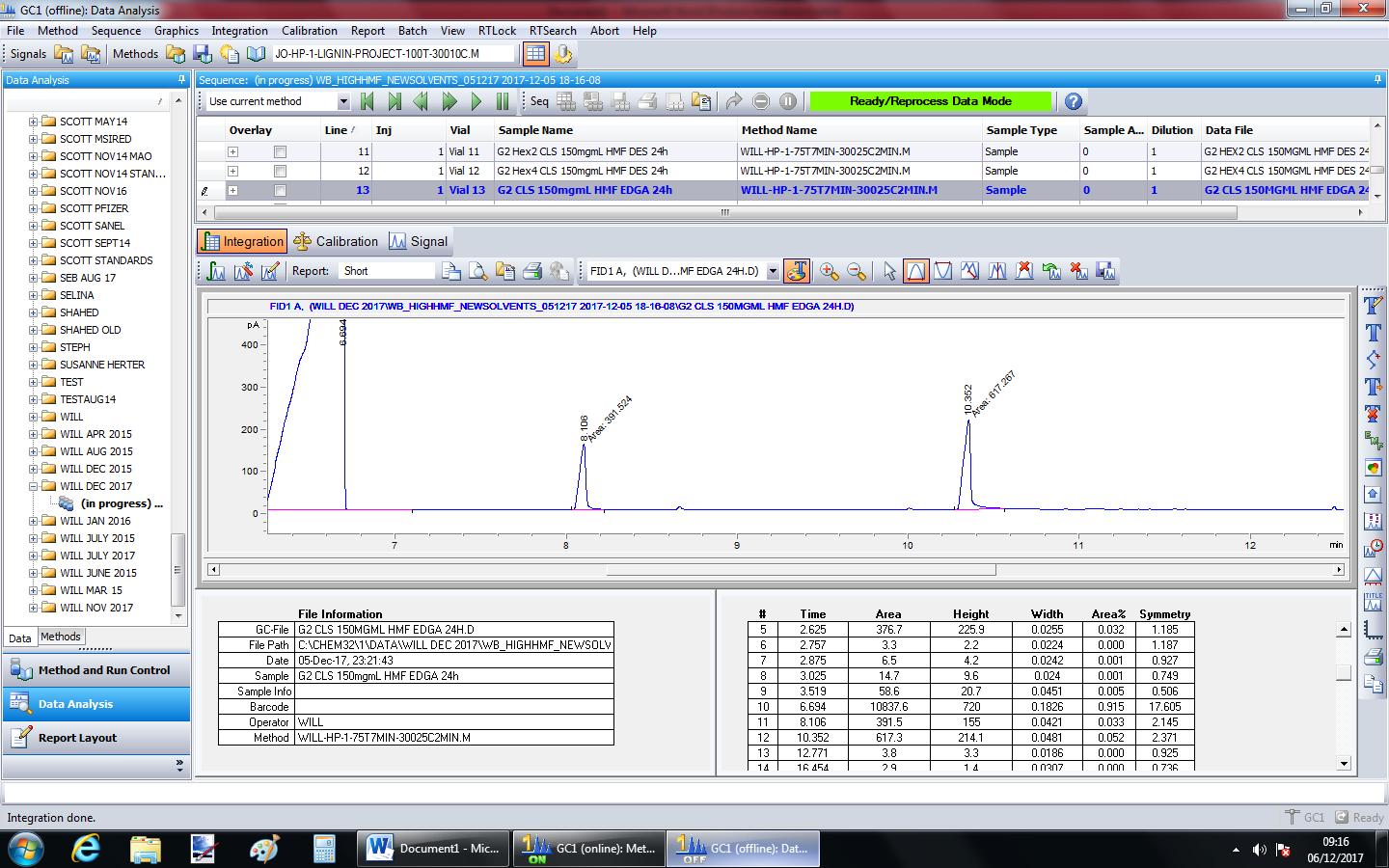


M_6-B_ 150 g/L HMF EGDA 24h (EtOAc phase, GC)


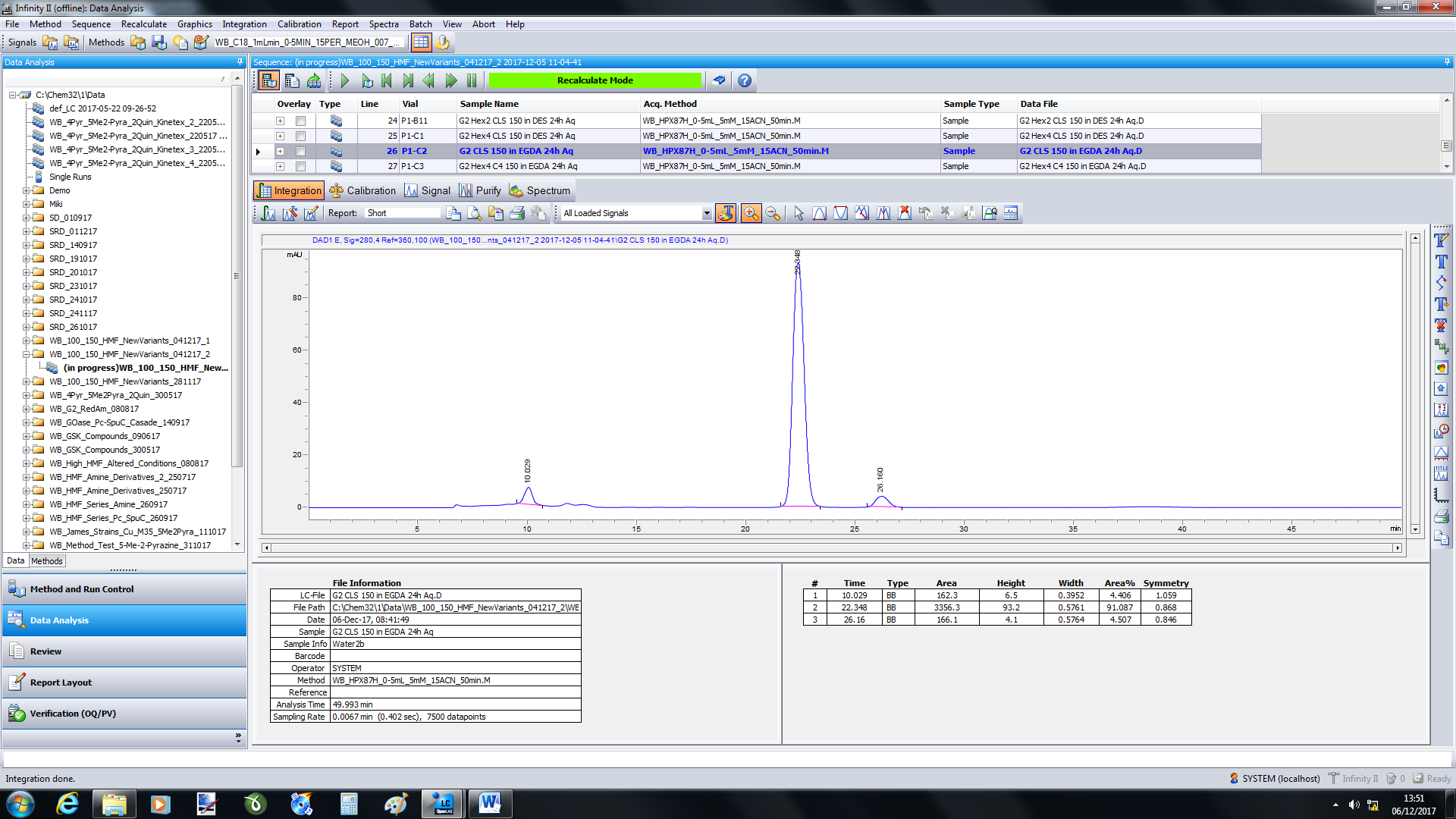


M_6-B_ 150 g/L HMF EGDA 24h (Aqueous phase, HPLC)


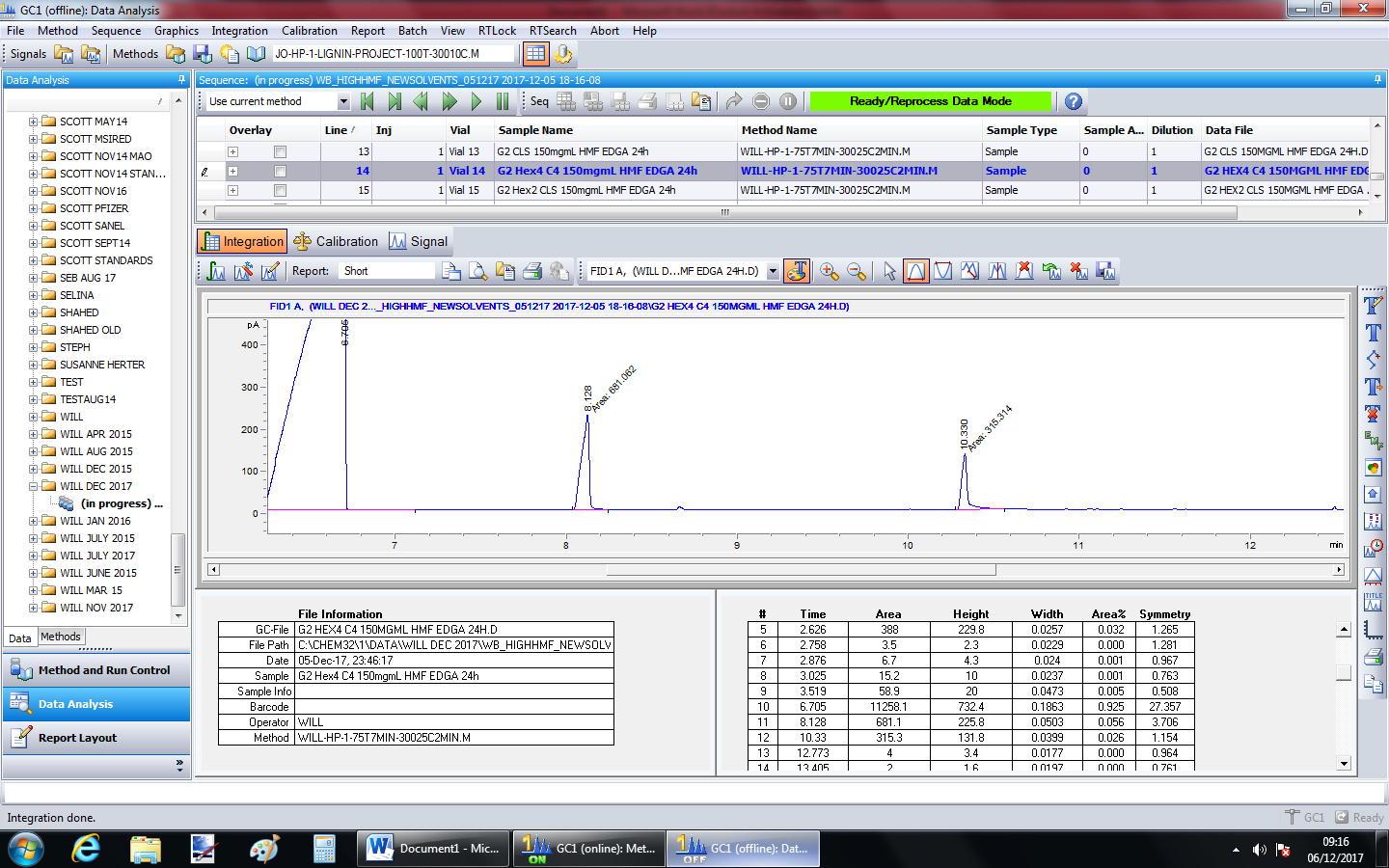


M_7-2A_ 150 g/L HMF EGDA 24h (EtOAc phase, GC)


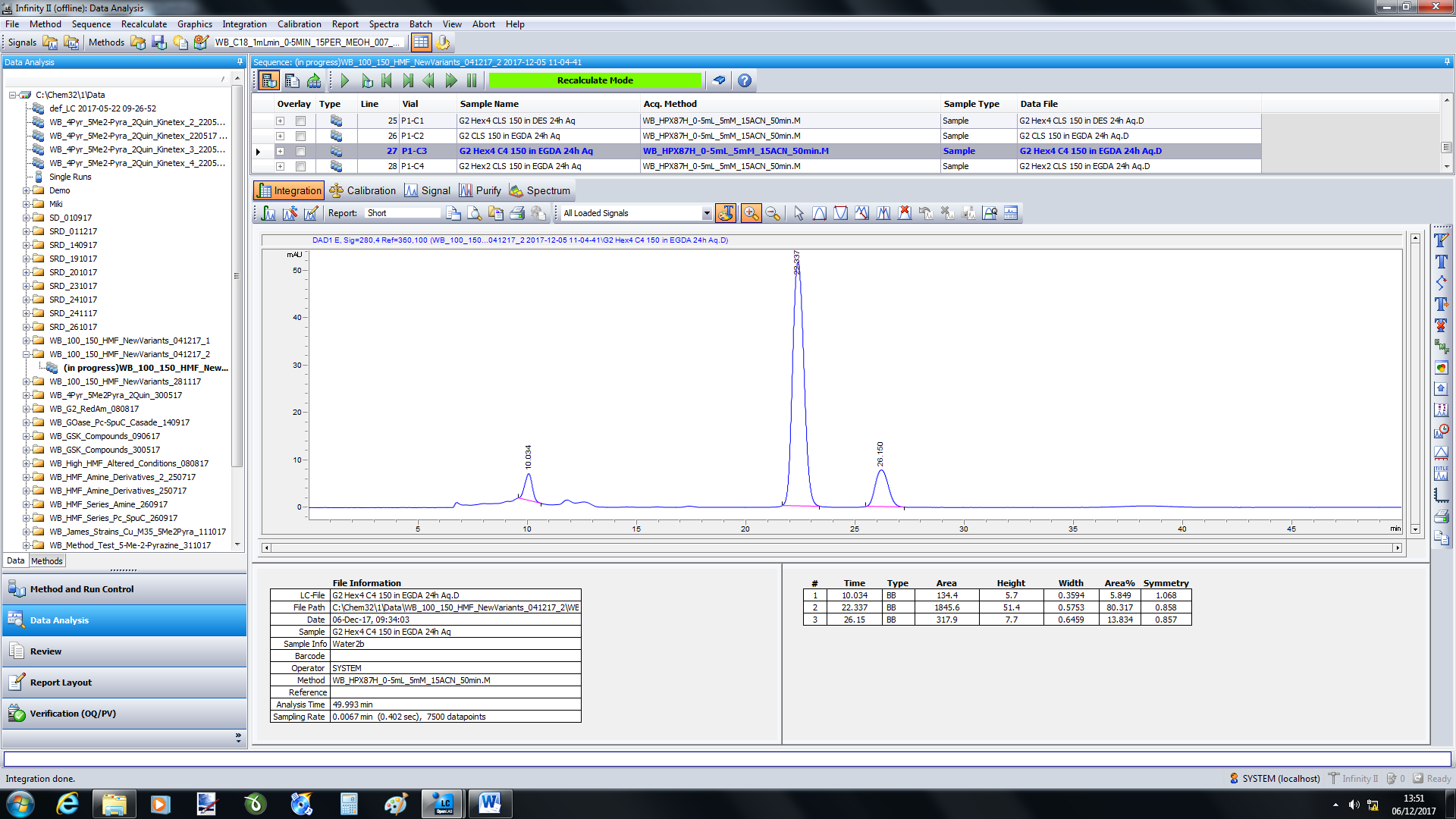


M_7-2A_ 150 g/L HMF EGDA 24h (Aqueous phase, HPLC)


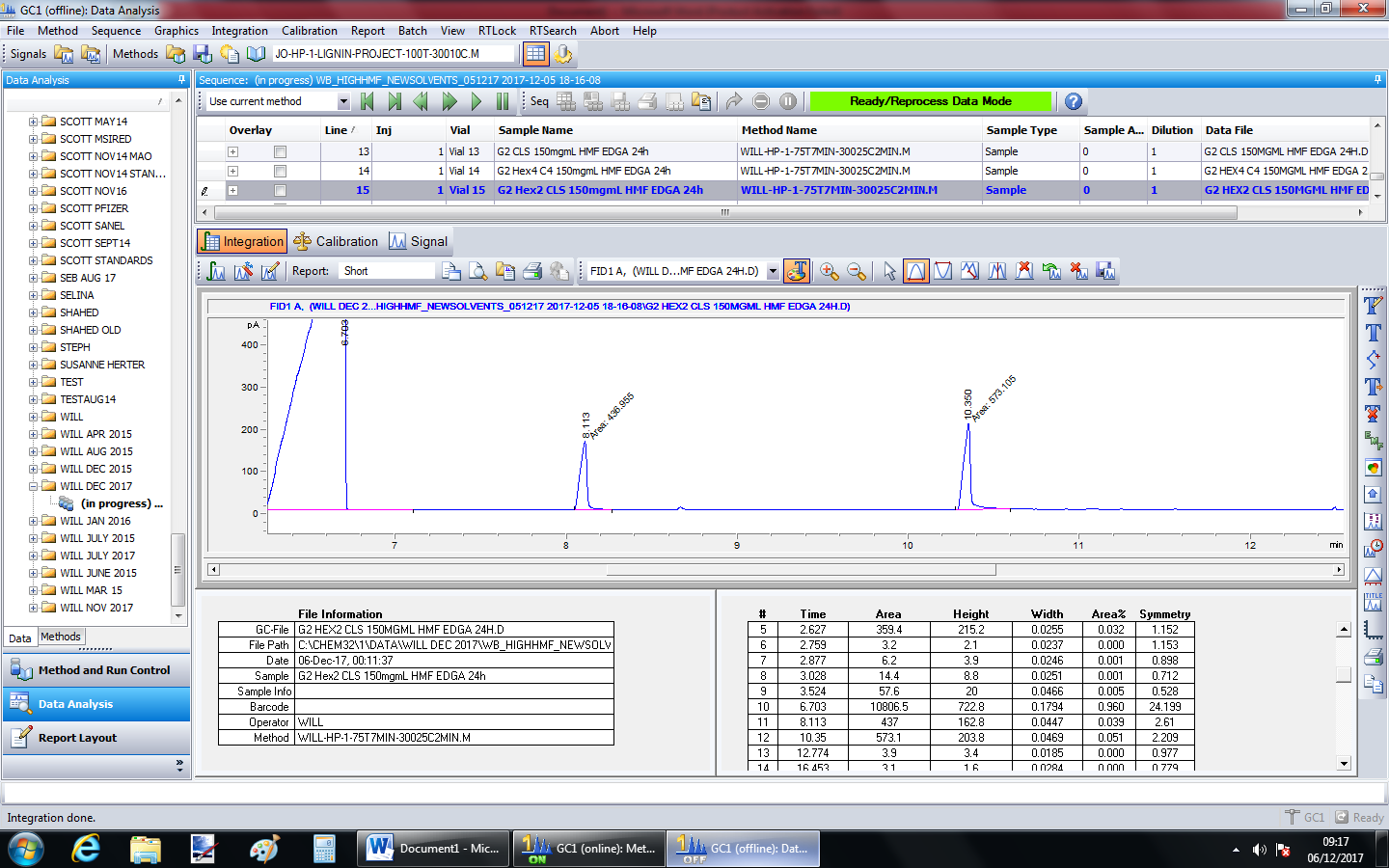


M_7-1B_ 150 g/L HMF EGDA 24h (EtOAc phase, GC)


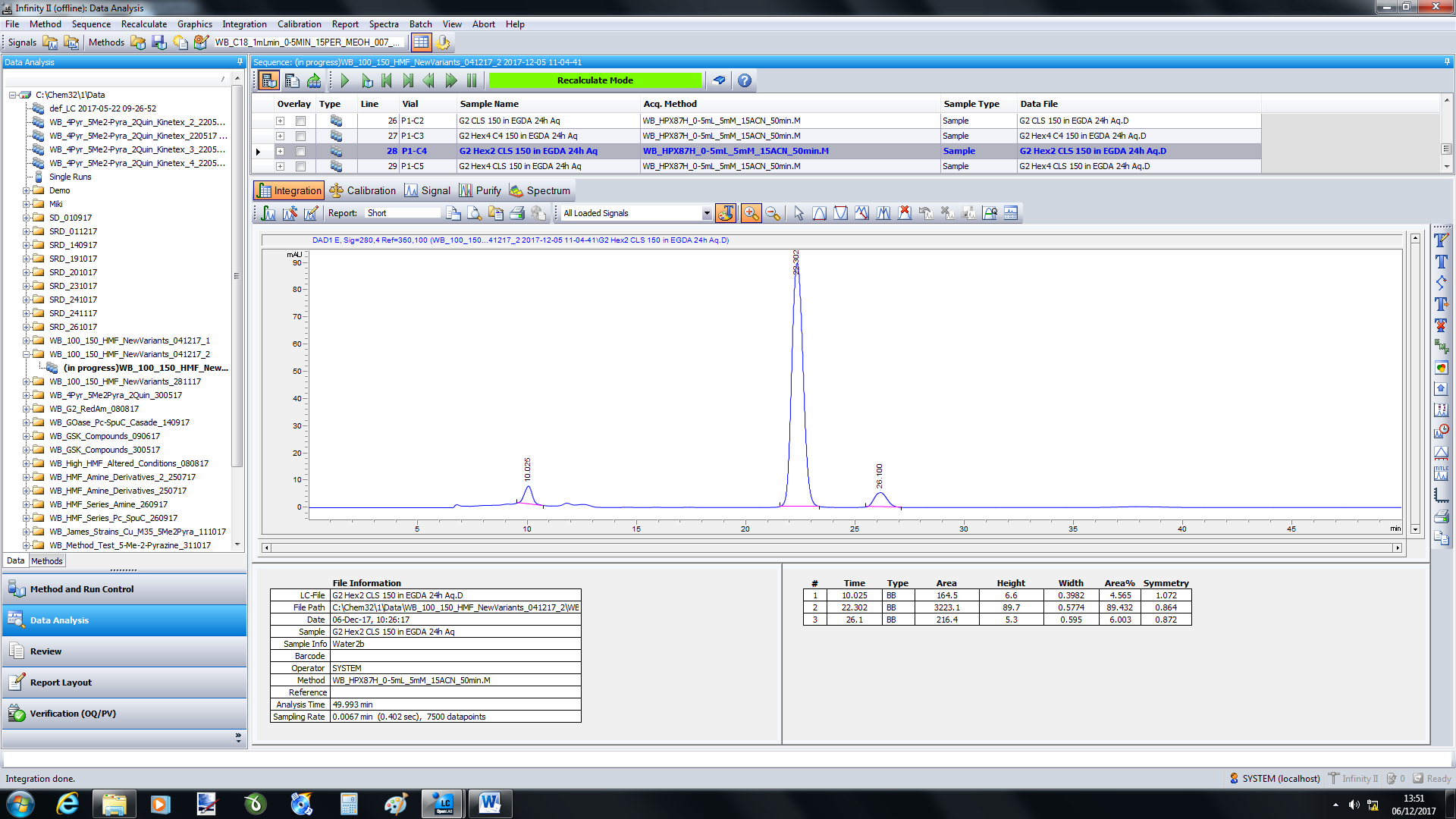


M_7-1B_ 150 g/L HMF EGDA 24h (Aqueous phase, HPLC)


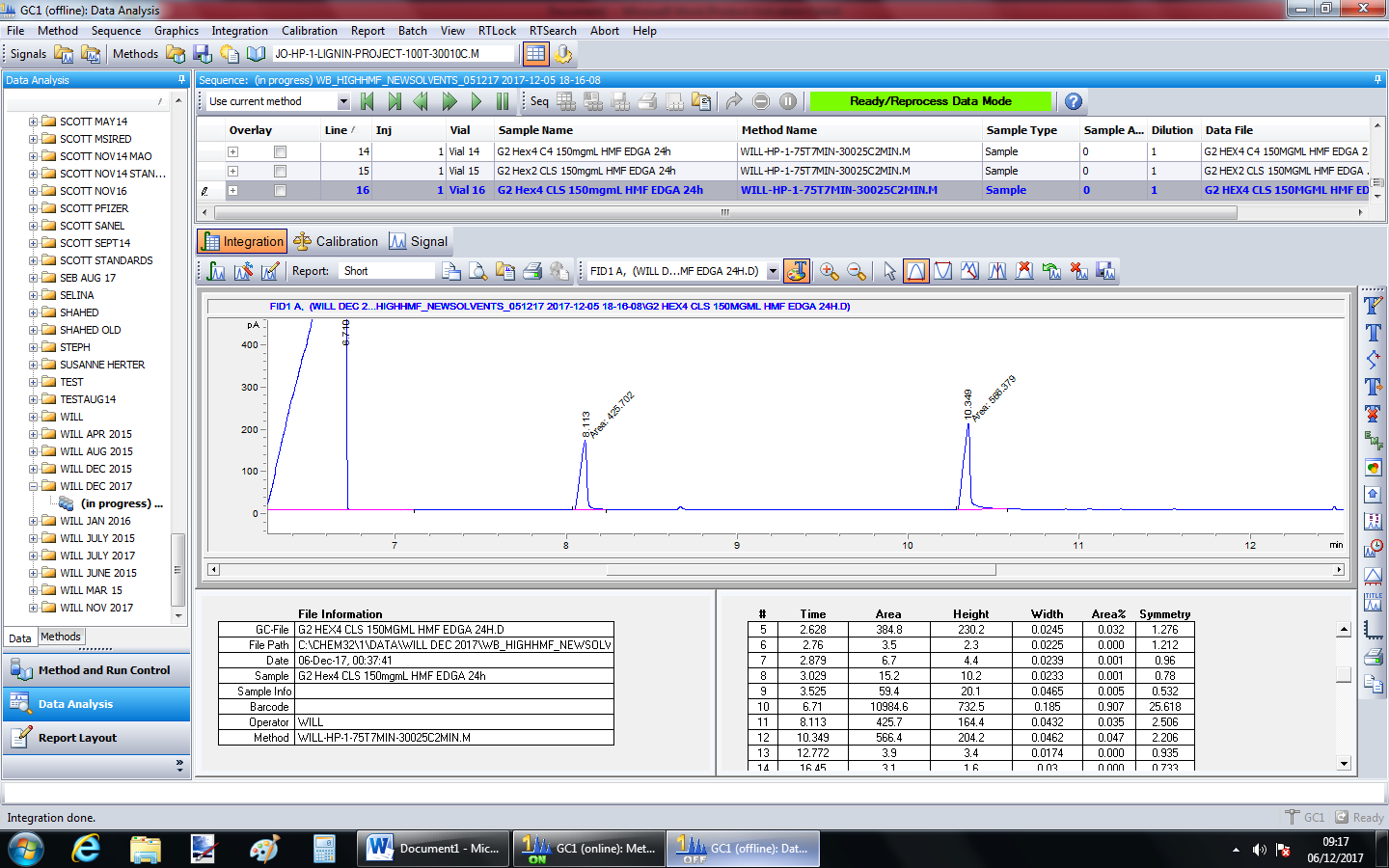


M_7-2B_ 150 g/L HMF EGDA 24h (EtOAc phase, GC)


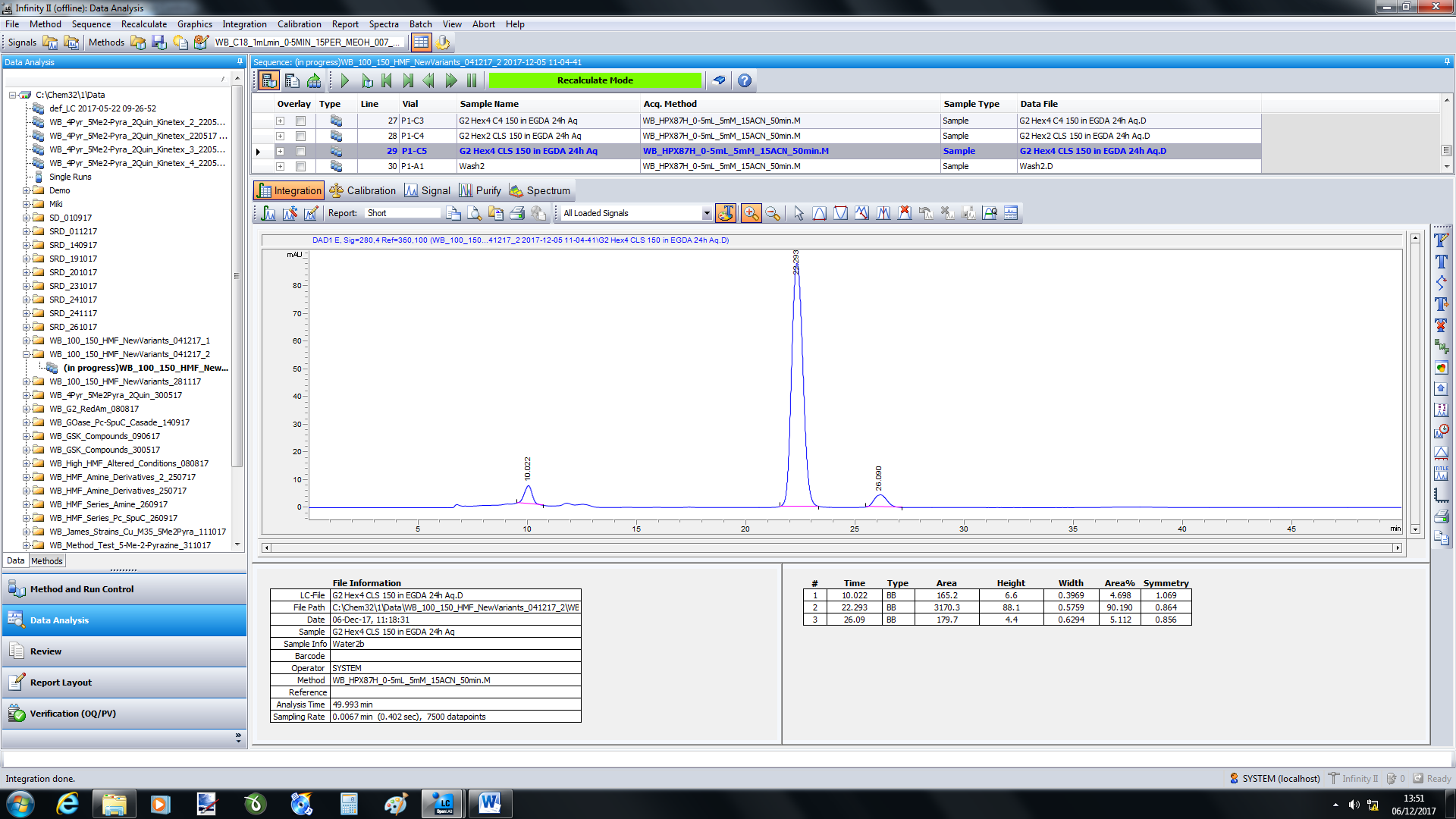


M_7-2B_ 150 g/L HMF EGDA 24h (Aqueous phase, HPLC)

The following data is presented in Supplementary Table 15, Entry 1:


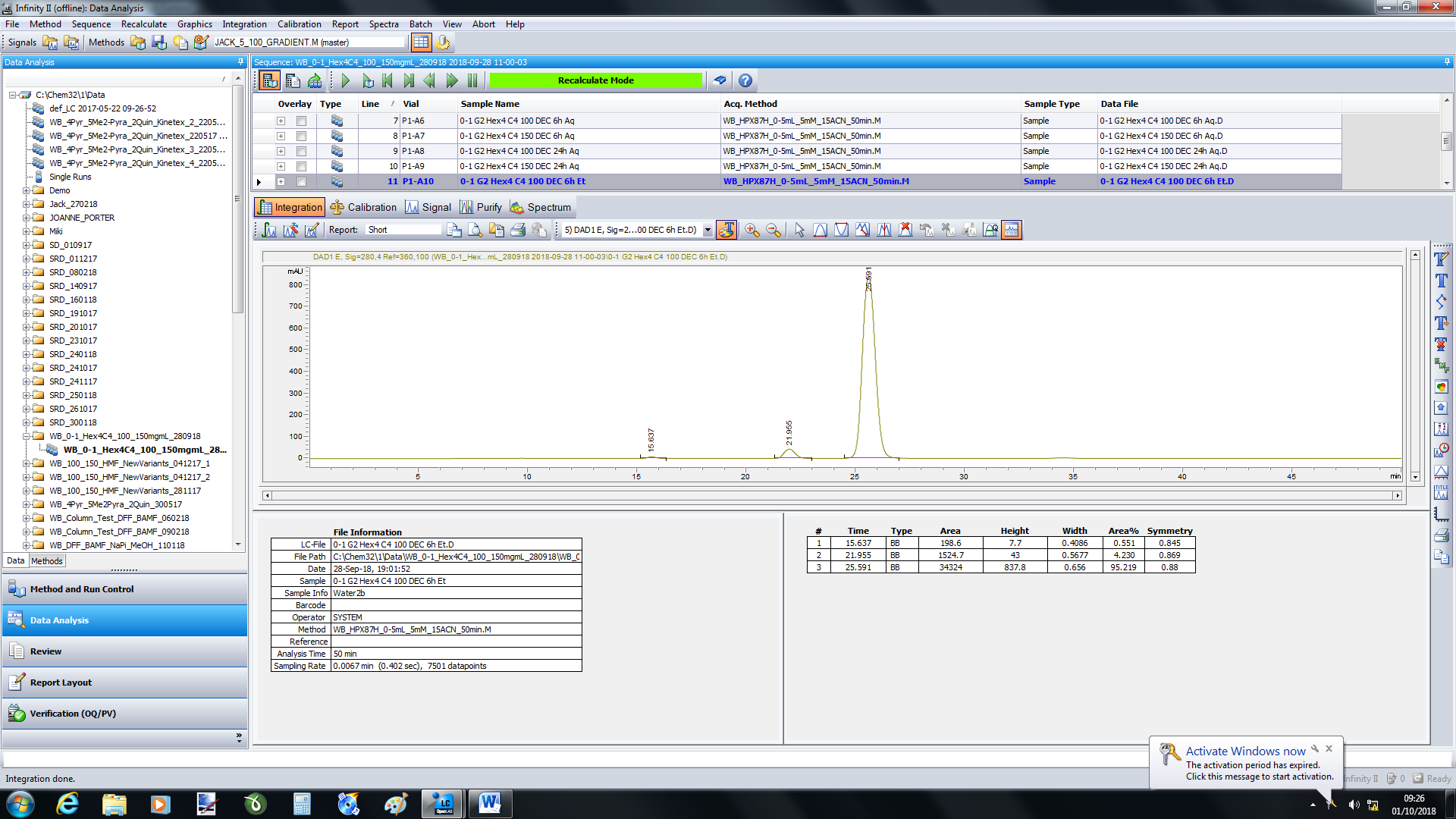


0.1 g/L M_7-2A_ 100 g/L HMF in DEC from BASF 6h (EtOAc phase)


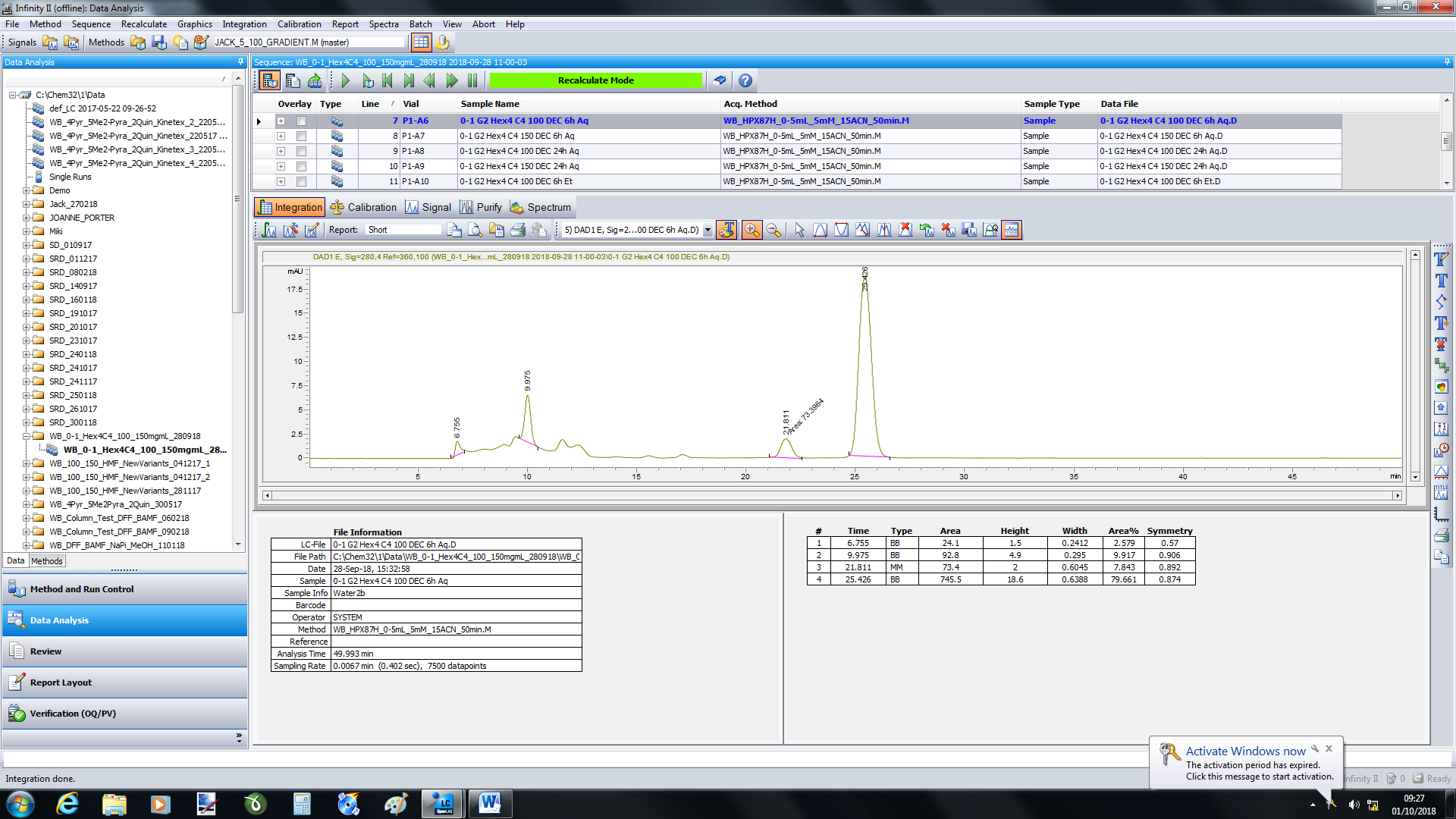


0.1 g/L M_7-2A_ 100 g/L HMF in DEC from BASF 6h (Aqueous phase)

# Supplementary References

1 Escalettes, F. & Turner, N. J. Directed evolution of galactose oxidase: generation of enantioselective secondary alcohol oxidases. *ChemBioChem* **9**, 857-860, (2008).

2 Rannes, J. B. *et al.* Glycoprotein labeling using engineered variants of galactose oxidase obtained by directed evolution. *J. Am. Chem. Soc.* **133**, 8436-8439, (2011).

3 Birmingham, W. R. & Turner, N. J. A single enzyme oxidative “cascade” via a dual-functional galactose oxidase. *ACS Catal.* **8**, 4025-4032, (2018).

4 Toftgaard Pedersen, A. *et al.* Process requirements of galactose oxidase catalyzed oxidation of alcohols. *Org. Process Res. Dev.* **19**, 1580-1589, (2015).

5 Deacon, S. E. & McPherson, M. J. Enhanced expression and purification of fungal galactose oxidase in *Escherichia coli* and use for analysis of a saturation mutagenesis library. *ChemBioChem* **12**, 593-601, (2011).

6 Delagrave, S. *et al.* Application of a very high-throughput digital imaging screen to evolve the enzyme galactose oxidase. *Protein Eng.* **14**, 261-267, (2001).

7 Wilkinson, D. *et al.* Structural and kinetic studies of a series of mutants of galactose oxidase identified by directed evolution. *Protein Eng. Des. Sel.* **17**, 141-148, (2004).

8 Bøje Madsen, M. *Enzymatic Synthesis of DFF from HMF using Galactose Oxidase* BSc thesis, Technical University of Denmark, (2019).

9 Toftgaard Pedersen, A. *Oxygen Dependent Biocatalytic Processes* PhD thesis, Technical University of Denmark, (2017).

10 Sun, L. H., Petrounia, I. P., Yagasaki, M., Bandara, G. & Arnold, F. H. Expression and stabilization of galactose oxidase in *Escherichia coli* by directed evolution. *Protein Eng.* **14**, 699-704, (2001).

11 Sun, L. H., Bulter, T., Alcalde, M., Petrounia, I. P. & Arnold, F. H. Modification of galactose oxidase to introduce glucose 6-oxidase activity. *ChemBioChem* **3**, 781-783, (2002).
